# scCOSMIX: A Mixed‐Effects Framework for Differential Coexpression and Transcriptional Interactions Modeling in Single‐Cell RNA‐Seq

**DOI:** 10.1002/sim.70213

**Published:** 2025-08-07

**Authors:** Anderson Bussing, Giampiero Marra, Daping Fan, Russell Shinohara, Danni Tu, Yen‐Yi Ho

**Affiliations:** ^1^ Department of Statistics University of South Carolina Columbia South Carolina USA; ^2^ Department of Statistical Science University College London London UK; ^3^ Department of Cell Biology and Anatomy University of South Carolina Columbia South Carolina USA; ^4^ Department of Biostatistics University of Pennsylvania Philadelphia Pennsylvania USA; ^5^ Regeneron Pharmaceuticals Tarrytown New York USA

**Keywords:** Differential co‐expression, hierarchical study design, mixed effects, single‐cell RNA‐seq, zero‐inflated copula model

## Abstract

Advancements in single‐cell RNA‐sequencing (scRNA‐seq) technologies generate a wealth of gene expression data that provide exciting opportunities for studying gene‐gene interactions systematically at individual cell resolution. Genetic interactions within a cell are tightly regulated and often highly dynamic in response to internal cellular signals and external stimuli. Evidence of these dynamic interactions can often be observed in scRNA‐seq data by examining conditional co‐expression changes. Existing approaches for studying these dynamic interaction changes in scRNA‐seq data do not address the multi‐subject hierarchical design commonly considered in single‐cell experiments. In this paper, we propose a Mixed‐effects framework for differential Coexpression and transcriptional interaction modeling in Single‐Cell RNA‐seq (scCOSMiX) to account for the cell‐cell correlation from the same individual. The proposed copula‐based approach allows the zero‐inflation, marginal, and association parameters to be modeled as functions of covariates with subject‐level random effects, to enable analyses to be tailored to the data under consideration. A series of simulation analyses were conducted to evaluate and compare the performance of scCOSMiX to other existing approaches. We applied the proposed method to both droplet and plate‐based scRNA‐seq data sets GSE266919 and GSE108989 to illustrate its applicability across distinct scRNA‐seq experimental protocols.

## 1 Introduction

The genetic system responsible for cellular development and function is regulated by intricate networks of thousands of genetic molecules [1]. Understanding how these genetic molecules interact with each other in biological systems is an ongoing focus in many biomedical studies [2, 3, 4]. Popular approaches for studying genetic interactions using gene expression measurements such as WGCNA [5], scLink [6], and graphical LASSO [7] assume static interactions between genes and do not seek to determine the dynamics of changes in genetic interactions in living cells. However, interactions between cellular molecules are likely to be tightly regulated and highly dynamic, varying across cell types, over cell age, or in response to perturbations such as drug treatment [8, 9]. The advent of single‐cell RNA sequencing (scRNA‐seq) has provided new opportunities to study these dynamic interaction changes with single‐cell resolution. To study such variation in gene‐gene interactions, researchers use differential co‐expression analysis, a framework for characterizing how gene‐gene relationships vary across different biological states or experimental conditions.

Effective estimation of differential co‐expression requires careful attention to the distributional properties of the gene expression data being modeled. In scRNA‐seq, gene expression measurements are often generated using one of two main experimental protocols: droplet‐based and plate‐based methods. Although both protocols produce non‐negative integer counts, the statistical distribution of these counts differs substantially between the two [10]. Specifically, droplet‐based methods, such as 10× Genomics Chromium [11], Drop‐seq [12], and inDrop [13], produce gene expression measurements characterized by high sparsity, shallow sequencing depth per cell, and lower amplification bias [10, 14]. In contrast, plate‐based methods, such as SMART‐seq2 [15], yield gene expression measurements with broader dynamic range, deeper sequencing depth per cell, and a greater prevalence of technical zeros arising from inefficient transcript capture and amplification biases [10, 14]. Extensive efforts have been made to identify flexible distributions for modeling the gene expression data generated by various protocols, with particular attention to whether zero‐inflated distributions are required [16]. Using real scRNA‐seq data sets, researchers applied AIC comparisons [17], Bayes factor comparisons [18], likelihood ratio tests [19], and zeros in excess of a negative binomial fit [20] to assess whether the additional complexity of a zero‐inflated model is warranted. Across these studies, support for zero‐inflated models was observed for 1%−10% of genes in droplet‐based data sets and 19%−50% of genes in plate‐based data sets [17, 18, 19, 20].

Beyond the choice of an appropriate modeling distribution, another major challenge in scRNA‐seq analysis is the proper handling of data sets containing cells from multiple patients. Such multipatient studies are becoming increasingly common, reflecting a shift toward designs intended to improve biomarker discovery and facilitate broader characterization of disease and treatment‐related effects [21, 22]. However, the hierarchical structure inherent in the resulting data presents unique statistical challenges. In particular, when multiple cells are sampled from the same individual, their expression profiles can be considered statistically dependent, as cells from the same subject tend to be more similar to each other than to cells from different individuals [23, 24]. Failure to account for this dependence can lead to inflated type I error rates and spurious discoveries, since treating dependent observations as independent exaggerates the effective sample size and undermines the validity of statistical inference [25, 26, 27]. Researchers have utilized GLMM [25], GEE [26], and pseudobulk approaches [23, 28] to address this within‐patient correlation in the context of differential expression, but such approaches have not yet been extended to differential coexpression. Most existing differential coexpression methods assume independence between all observations, making them unsuitable for multipatient studies [8, 29, 30, 31, 32, 33].

Together, these characteristics of scRNA‐seq data give rise to a distinct set of challenges for differential coexpression analysis in multipatient settings. Methods must address the dependence of gene‐gene coexpression on covariates, the within‐subject correlation among cells, and the count‐based nature of gene expression measurements, including the potential for excess zeros. Existing approaches differ in how fully they account for these challenges. The Liquid Association (LA) framework introduced by Li [8, 29] provided one of the first formal approaches to modeling dynamic changes in gene‐gene relationships, but its normality assumptions and permutation‐based inference make it poorly suited to the count‐based characteristics and hierarchical structure of scRNA‐seq data. Ho et al. (2010) [30] proposed a likelihood‐based extension of LA called CNM, which modeled the expression of two genes as conditionally bivariate Gaussian. Tu et al. (2022) [31] employed a similar bivariate Gaussian model, named CoCoA, with estimation carried out using restricted maximum likelihood. While both models boast fast computation times and enjoy the computational advantages of the Gaussian family, they do not accommodate the count nature of the data or the within‐subject correlation.

To address the restrictive normality assumptions of earlier frameworks, Yang and Ho [32] introduced a model called ZENCO, which models coexpression in count data using a zero‐inflated Poisson‐Gamma mixture with Gaussian copula dependence. Building on this work, Ma et al. [34] proposed a flexible copula model accommodating a range of marginal distributions and allowing for covariate effects in the zero‐inflation parameters. These approaches, implemented in the R package scDECO [35], adopt a Bayesian framework with Markov chain Monte Carlo (MCMC) estimation, enabling robust uncertainty quantification. However, they do not account for the hierarchical structure of multipatient data, and their reliance on MCMC introduces substantial computational costs, limiting scalability to larger datasets.

Motivated by the need for faster and more scalable inference, Su et al. [33] proposed CS‐CORE, a model‐based approach capable of efficiently estimating co‐expression structures for potentially thousands of genes. However, the method does not allow for covariates in the mean or in the dependence structure, and it relies on permutation‐based strategies to assess differential co‐expression between conditions. As a result, CS‐CORE is not readily extendable to settings in which co‐expression evolves smoothly with continuous covariates such as pseudotime, treatment dosage, or patient characteristics, and it also does not account for within‐subject correlation.

Thus far, none of the methods discussed simultaneously account for all three of the core challenges described above. Seeking a unified solution, we build upon the flexible bivariate copula regression framework introduced by Marra and Radice (2017) [36] and extended by van der Wurp et al. (2020) [37]. The methods by Marra and van der Wurp, implemented in popular R package GJRM [38], link the marginal parameters as well as the copula association parameter to additive predictors through one‐to‐one transformations and estimate all model components jointly through penalized maximum likelihood. To adapt this framework for differential co‐expression analysis of multi‐patient scRNA‐seq data, we incorporate joint‐level covariate‐dependent zero‐inflation, introduce patient‐specific random effects through the additive predictor structure, and include offsets in the marginal means to adjust for sequencing depth variation.

The structure of the paper is as follows. First, we introduce the droplet‐based and plate‐based data sets which motivate the design of our simulation studies and serve as the focus of our real data analyses. Second, we formally define the scCOSMiX model and outline the nested optimization approach used for penalized maximum likelihood estimation. Next, through a series of simulation studies, we assess the statistical power, coverage, robustness, FDR control, precision‐recall, and computation time of our method alongside existing methods. Finally, we present the results of our real data analyses, where we apply scCOSMiX to the aforementioned data sets and identify significant differentially co‐expressed gene pairs.

## 2 Materials and Methods

### 2.1 Data Sets

To demonstrate the flexibility of scCOSMiX across different scRNA‐seq protocols, we conduct all analyses using a droplet‐based and a plate‐based data set. The droplet‐based data set comes from a triple‐negative breast cancer (TNBC) study by Zhang et al. [39] (GEO accession GSE266919). In this study, tumor biopsies were collected from patients before and after treatment with paclitaxel, nab‐paclitaxel, or their combinations with the anti‐PD‐L1 antibody atezolizumab. Single‐cell RNA sequencing was performed using the 10× Genomics Chromium platform. Immune cell dynamics were profiled across treatment regimens, revealing treatment‐associated shifts in the abundance and transcriptional states of T cells, B cells, myeloid cells, and natural killer cells. Myeloid cells were found to undergo transcriptional reprogramming in response to treatment with nab‐paclitaxel, and this finding serves as the motivation for our real data analysis of this data set.

We investigate changes in gene‐gene co‐expression between pre‐treatment and post‐treatment myeloid cells from patients classified as responders to paclitaxel‐based therapies. The data set includes pre‐ and post‐treatment samples from 44 patients, annotated with treatment regime, cell type, and responder/non‐responder status. For our analysis, we filter the data set to include the myeloid cells of patients who had their biopsies taken from breast tissue; had at least 30 myeloid cells in both their pre‐ and post‐treatment samples; were treated with nab‐paclitaxel or nab‐paclitaxel plus atezolizumab, and were classified as responders. This resulted in a final set of 8 patients and 5018 cells (2215 cells pre‐treatment and 2803 cells post‐treatment).

The plate‐based data set comes from a colorectal cancer (CRC) study by Zhang et al. [40] (GEO accession GSE108989). In this study, samples from tumor tissue, adjacent normal tissue, and peripheral blood were collected from 12 CRC patients who had not received chemotherapy or radiation therapy. T cells were isolated and profiled using the Smart‐seq2 protocol, and clonal expansion and transcriptional diversity across the T cell compartment were profiled, with particular focus given to the CD4 and CD8 subsets. The CD4 subset was found to be characterized by transcriptional profiles associated with immune regulation and helper function, while the CD8 subset showed evidence of clonal expansion and expressed genes linked to cytotoxicity and exhaustion. In our real data analysis of this dataset, we examine how these subset‐specific characteristics are differentially reflected in patterns of gene‐gene co‐expression. To this end, we filter the data to just CD4 and CD8 T cells derived from tumor tissue, and we retain only patients with at least one cell from each lineage. This results in a final analysis set of 11 patients and 4032 cells (2466 CD4 cells and 1566 CD8 cells).

### 2.2 Model

We start by considering the expression measurements for a gene pair. Let $Y_{1}={\left[\begin{array}{llll} Y_{11} & Y_{21} & \ldots & Y_{n1} \end{array}\right]}^{⊤}$, $Y_{2}={\left[\begin{array}{llll} Y_{12} & Y_{22} & \ldots & Y_{n2} \end{array}\right]}^{⊤}$ represent the expression levels of two genes in n cells. Thus, $Y_{ij}$ represents the expression level in gene j in cell i, where i∈{1,…,n}, j∈{1,2}. Let X and Z be design matrices for fixed effects and random effects, respectively. In the scCOSMiX framework, the joint expression of $Y_{i1},Y_{i2}$ is modeled using a zero‐inflated bivariate copula. Every parameter in the model can be modeled as a linear combination of relevant fixed and random effects via an appropriate one‐to‐one transformation. We first outline the joint pmf of $Y_{i1},Y_{i2}$ in a single cell, before expanding it to the joint pmf of $Y_{1},Y_{2}$.

The flexibility of the copula model allows arbitrary marginals to be considered in the scCOSMiX framework. In this paper, we apply the negative binomial marginal distributions for scRNA‐seq count data. Let $F_{\text{NB}}$ be the negative binomial cdf under the NBII parameterization from Greene [41]. This distribution has two parameters: a mean parameter μ and an over‐dispersion parameter σ and has expectation μ and variance $\mu +\mu^{2}\sigma$. The σ is referred to as the over‐dispersion parameter because it controls how much larger the variance is than the mean. As σ moves toward 0, the variance becomes equal to the mean and the distribution becomes a Poisson (μ) distribution, but as σ moves away from 0, the variance becomes increasingly larger than the mean.

Dependence between $Y_{i1}$ and $Y_{i2}$ is imparted through a bivariate copula. While various copulas, including the Clayton [42], Joe [43], Gumbel [44], and Frank [45], are compatible with our proposed framework, in this paper we focus on the Gaussian copula. The Gaussian copula is a popular choice in the literature for analyzing scRNA‐seq data [34, 46, 47, 48], and its association parameter ρ, being the correlation coefficient of a multivariate Gaussian distribution, is readily interpretable. The cdf of a bivariate Gaussian copula with negative binomial marginals takes the form:

$$\begin{array}{ll} & G\left(y_{i1},y_{i2};\;\mu_{i1},\sigma_{i1},\mu_{i2},\sigma_{i2},\rho_{i}\right)\\ & \;=P\left(Y_{i1}\leq y_{i1},\;Y_{i2}\leq y_{i2};\;\mu_{i1},\sigma_{i1},\mu_{i2},\sigma_{i2},\rho_{i}\right)\\ & \;=\Phi_{2}\left(\Phi^{-1}\left\{F_{\text{NB}}(y_{i1};\;\mu_{i1},\sigma_{i1})\right\},\;\Phi^{-1}\left\{F_{\text{NB}}(y_{i2};\;\mu_{i2},\sigma_{i2})\right\};\;\rho_{i}\right) \end{array}$$

where $\Phi_{2}(\cdot ,\cdot ;\rho_{i})$ denotes the cdf of the bivariate standard normal distribution with correlation $\rho_{i}$, and $\Phi^{-1}$ is the quantile function of the standard normal distribution. Similarly, the joint pmf of $Y_{i1}$ and $Y_{i2}$, before accounting for zero‐inflation, is given by:

(1) $$\begin{array}{cc} & g\left(y_{i1},y_{i2};\;\mu_{i1},\sigma_{i1},\mu_{i2},\sigma_{i2},\rho_{i}\right)\\ & \;=P\left(Y_{i1}=y_{i1},\;Y_{i2}=y_{i2};\;\mu_{i1},\sigma_{i1},\mu_{i2},\sigma_{i2},\rho_{i}\right)\\ & \;=G\left(y_{i1},y_{i2};\;\mu_{i1},\sigma_{i1},\mu_{i2},\sigma_{i2},\rho_{i}\right)\\ & \;-G\left(y_{i1}-1,y_{i2};\;\mu_{i1},\sigma_{i1},\mu_{i2},\sigma_{i2},\rho_{i}\right)\\ & \;-G\left(y_{i1},y_{i2}-1;\;\mu_{i1},\sigma_{i1},\mu_{i2},\sigma_{i2},\rho_{i}\right)\\ & \;+G\left(y_{i1}-1,y_{i2}-1;\;\mu_{i1},\sigma_{i1},\mu_{i2},\sigma_{i2},\rho_{i}\right) \end{array}$$

To incorporate zero‐inflation into this model, we introduce parameters $p_{i1}$ and $p_{i2}$, which represent the probabilities of a dropout event affecting gene 1 and gene 2, respectively, in cell i. The scCOSMiX joint pmf for $Y_{i1},Y_{i2}$ is then defined as a mixture model, where these dropout probabilities act as weights:

(2) $$\begin{array}{cc} & f(y_{i1},y_{i2}\;;\;\mu_{i1},\sigma_{i1},\mu_{i2},\sigma_{i2},\rho_{i},p_{i1},p_{i2})\\ & \;=P\left(Y_{i1}=y_{i1},\;Y_{i2}=y_{i2}\;;\;\mu_{i1},\sigma_{i1},\mu_{i2},\sigma_{i2},\rho_{i},p_{i1},p_{i2}\right)\\ & =(1-p_{i1})(1-p_{i2})\;g(y_{i1},y_{i2}\;;\;\mu_{i1},\sigma_{i1},\mu_{i2},\sigma_{i2},\rho_{i})\\ & \;+\left\{\begin{array}{ll} 0\; & \text{if}\;y_{i1}>0\;\text{and}\;y_{i2}>0\\ (1-p_{i1})\;p_{i2}\;f_{\text{NB}}(y_{i1}\;;\;\mu_{i1},\sigma_{i1})\; & \text{if}\;y_{i1}>0\;\text{and}\;y_{i2}=0\\ p_{i1}(1-p_{i2})\;f_{\text{NB}}(y_{i2}\;;\;\mu_{i2},\sigma_{i2})\; & \text{if}\;y_{i1}=0\;\text{and}\;y_{i2}>0\\ p_{i1}p_{i2}+(1-p_{i1})\;p_{i2}\;f_{\text{NB}}(y_{i1}\;;\;\mu_{i1},\sigma_{i1})\; & \\ \;+p_{i1}(1-p_{i2})\;f_{\text{NB}}(y_{i2}\;;\;\mu_{i2},\sigma_{i2})\; & \text{if}\;y_{i1}=0\;\text{and}\;y_{i2}=0 \end{array}\right. \end{array}$$

The marginal distributions of this joint pmf are given by:

$$f_{j}(y_{ij};\;\mu_{ij},\sigma_{ij},p_{ij})=\left\{\begin{array}{ll} p_{ij}+(1-p_{ij})\;f_{\text{NB}}(y_{ij};\;\mu_{ij},\sigma_{ij})\; & \text{if}\;y_{ij}=0\\ (1-p_{ij})\;f_{\text{NB}}(y_{ij};\;\mu_{ij},\sigma_{ij})\; & \text{if}\;y_{ij}>0 \end{array}\right.$$

which are zero‐inflated negative binomial pmfs.

The parameters $\Theta =\{\mu_{i1},\mu_{i2},\sigma_{i1},\sigma_{i2},\rho_{i},p_{i1},p_{i2}\}$ can be covariate‐dependent in the following way:

(3) $$\begin{array}{cc} \log\left\{\mu_{i1}\right\} & =X_{i,\mu_{1}}\beta_{1}+Z_{i,\mu_{1}}u_{\mu_{1}}+\log\left(S_{i}\right)\\ \log\left\{\mu_{i2}\right\} & =X_{i,\mu_{2}}\beta_{2}+Z_{i,\mu_{2}}u_{\mu_{2}}+\log\left(S_{i}\right)\\ \log\left\{\sigma_{i1}\right\} & =X_{i,\sigma_{1}}\alpha_{1}+Z_{i,\sigma_{1}}u_{\sigma_{1}}\\ \log\left\{\sigma_{i2}\right\} & =X_{i,\sigma_{2}}\alpha_{2}+Z_{i,\sigma_{2}}u_{\sigma_{2}}\\ \text{atanh}\left\{\rho_{i}\right\} & =X_{i,\rho }\tau +Z_{i,\rho }u_{\rho }\\ \text{logit}\left\{p_{i1}\right\} & =X_{i,p_{1}}\kappa_{1}+Z_{i,p_{1}}u_{p_{1}}\\ \text{logit}\left\{p_{i2}\right\} & =X_{i,p_{2}}\kappa_{2}+Z_{i,p_{2}}u_{p_{2}} \end{array}$$

where the log,atanh, and logit transformations on the left‐hand side ensure that each parameter remains within its valid parameter space. $X_{\theta }$ and $Z_{\theta }$ are submatrices of the full design matrices X and Z, containing only the columns relevant to parameter θ. Following the approach taken by Su et al. [33] and Mallick et al. [16], the $\log(S_{i})$ term is an offset equal to the logarithm of the total sequencing depth of cell i. The random effects design matrix $Z_{\theta }$ is structured such that each row is a standard basis vector indicating the category of the grouping factor for that observation. For example, if we are fitting a patient‐level random intercept for θ in a 4‐patient data set, and cell i belongs to patient 2, then $Z_{i,\theta }=\left[\begin{array}{llll} 0 & 1 & 0 & 0 \end{array}\right]$ [49]. The random effects $u_{\theta }$ follow a normal distribution $u_{\theta }∼N\left(0,\;\sum_{\theta }\right)$, where $\sum_{\theta }$ is a diagonal covariance matrix whose entries share a common value for components of $u_{\theta }$ that with the same variance component, and distinct values for components associated with different variance components. So in the 4‐patient random intercept example, the covariance matrix could be $\sum_{\theta }=\text{diag}\left\{\left(c,c,c,c\right)\right\}$.

This random effects structure falls within the class of mixed models laid out by Wood [50], which can be fit using penalized log‐likelihood. Rather than incorporating the distribution of the random effects into the likelihood, they are instead treated as parameters and shrunk toward 0 by a penalty. Gathering all fixed effects coefficients into one vector δ, and all random effects coefficients into one vector u, we arrive at the penalized log‐likelihood for the full data set $y_{1},y_{2}$:

(4) $$\begin{array}{c} l\left(\delta ,u;Y_{1},Y_{2}\right)=\overset{n}{\underset{i=1}{\sum }}\log\left(f(y_{i1},y_{i2};\;\delta ,u)\right)-\frac{1}{2}\underset{\theta \in \Theta }{\sum }u_{\theta }^{⊤}\text{diag}\left\{\lambda_{\theta }\right\}u_{\theta } \end{array}$$

The penalty vector $\lambda_{\theta }$ is structured to align with $\text{diag}\left(\sum_{\theta }\right)$, such that all components of $u_{\theta }$ corresponding to the same variance group receive the same penalty. Note that the normal distribution of the random effects does not appear in the above log‐likelihood. The u from Equation (3) are treated as parameters just like the δ, except that the u are subject to the ridge penalty while the δ are not [51].

### 2.3 Estimation

The estimation of the penalized log‐likelihood Equation (4) is carried out using the nested optimization approach from Marra et al. [52]. In the outer iteration of this procedure, the smoothing parameters $\lambda_{p_{1}},\lambda_{p_{2}},\lambda_{\mu_{1}},\ldots ,\lambda_{\rho }$ are estimated by minimizing an expression analogous to the Akaike information criterion but with effective degrees of freedom used instead of number of parameters. In the inner iteration, the smoothing parameters are kept constant, and Equation (4) is maximized over δ,u.

Maximizing the log‐likelihood in the inner iteration is achieved using trust region optimization [53]. In trust region optimization, a quadratic approximation of the objective function, centered at the current iterate, is formed using the gradient and Hessian of the objective function (derivations of the gradient and Hessian are provided in Appendix 3). Then, a candidate step is chosen as the step that maximizes the quadratic surface within a previously defined trust region around the current iterate. The increase in the actual objective function that results from taking that candidate step is compared to the increase predicted by the quadratic approximation. If the actual increase is significantly smaller than was predicted, then we reject the candidate step, decrease the trust region radius, and try again. Otherwise, the candidate step is taken, and if the step is sufficiently large and the quadratic approximation sufficiently accurate, then the trust region radius is increased. The algorithm terminates when a step is taken that increases either the objective function or the quadratic approximation to the objective function by less than $\sqrt{\text{.Machine}\$\text{double.eps}}$.

The benefit of iteratively expanding and shrinking the trust region radius is that small steps are taken in locations where the quadratic model is a poor approximation to the true objective function, and large steps are taken in locations where it is a good approximation. This allows the algorithm to search carefully in difficult regions and to stride confidently in straightforward ones. Consider an example objective function that has a long flat ridge: while a line‐search algorithm would potentially take a very long step along the ridge, thus bypassing potentially important features and reducing efficiency, the trust region algorithm's step sizes are limited by the trust region radius. Another advantage of trust region optimization is that the quadratic model it uses to approximate the objective function protects against potentially non‐definite evaluations of the actual objective function. This is because steps that lead to non‐definite evaluations of the approximate objective function are never taken and thus the actual objective function is never exposed to such inputs. A comprehensive discussion of trust region optimization can be found in [53].

Note that trust region optimization requires starting values to be provided for each parameter. Starting values for the zero‐inflation parameters $\kappa_{1},\kappa_{2},u_{p_{1}},u_{p_{2}}$ are obtained by fitting $\text{logit}\left\{P(Y_{i1}=0)\right\}=X_{iS_{p_{1}}}\kappa_{1}+Z_{iS_{p_{1}}^{\prime }}u_{p_{1}}$ and $\text{logit}\left\{P(Y_{i2}=0)\right\}=X_{iS_{p_{2}}}\kappa_{2}+Z_{iS_{p_{2}}^{\prime }}u_{p_{2}}$ using logistic regression from R package mgcv [49], with weights of 0.5 assigned to $Y_{ij}=0$ observations and weights of 1 assigned to $Y_{ij}>0$ observations. Starting values for the remaining parameters are obtained using the methods from Marra and Radice [36], again following the same weighting scheme as outlined above.

Once the algorithm has converged, we use the observed Hessian of the penalized log‐likelihood, along with the point estimates, to obtain confidence intervals and carry out hypothesis tests about the different parameters as laid out in [51].

## 3 Simulation Studies

To assess the performance of scCOSMiX and compare it with existing methods, we conduct six simulation studies: power, coverage, robustness, FDR control, precision‐recall, and computation time. Each study is carried out under a droplet‐based and a plate‐based setup, as described in Section 2.1. To evaluate the performance of scCOSMiX in terms of power, coverage, and robustness, we simulate data from our proposed model using the following procedure:

1. Simulate random effect realizations from $u_{\theta }∼N\left(0,\;\sum_{\theta }\right)$. Then for i=1,…,n,
2. Calculate $\mu_{i1},\mu_{i2},\sigma_{i1},\sigma_{i2},\rho_{i},p_{i1},p_{i2}$ as defined in Equation (3), given specified coefficients, random effects, and offsets.
3. Simulate ${\left(z_{i1},z_{i2}\right)}^{⊤}$ from $N_{2}\left(0,1,\rho_{i}\right)$; the standard bivariate Gaussian distribution with correlation coefficient $\rho_{i}$.
4. For j=1,2, set $v_{ij}=F_{\text{NB}}^{-1}\left\{\Phi \left(z_{ij}\right);\mu_{ij},\sigma_{ij}\right\}$.
5. Simulate $\zeta_{ij}∼\text{Bern}(p_{ij})$, where $\zeta_{ij}=1$ indicates that $v_{ij}$ is dropped due to zero‐inflation.
6. Set $y_{ij}=(1-\zeta_{j})v_{ij}$.

The parameters utilized in the power, coverage, and robustness simulation scenarios are chosen based on the analyses in Sections 4.1 and 4.2. We use gene pair (*GPNMB*, *SGK1*) from Table 4 for the droplet case, and gene pair (*BATF*, *GLRX*) from Table S5 for the plate case. These parameter values are:

(5) $$\begin{array}{cc} & {\left[\beta_{01},\beta_{11},\beta_{02},\beta_{12},\alpha_{01},\alpha_{11},\alpha_{02},\alpha_{12},\tau_{0},\tau_{1},p_{01},p_{11},p_{02},p_{12}\right]}^{⊤}\\ & \;=[-7.26,\;0.45,\;-7.10,\;0.20,\;-0.46,\;-0.19,\;-0.44,\\ & \;{-0.04,\;0.06,\;-0.20,\;0.01,\;0.05,\;0.01,\;0.01]}^{⊤} \end{array}$$

and

(6) $$\begin{array}{cc} & {\left[\beta_{01},\beta_{11},\beta_{02},\beta_{12},\alpha_{01},\alpha_{11},\alpha_{02},\alpha_{12},\tau_{0},\tau_{1},p_{01},p_{11},p_{02},p_{12}\right]}^{⊤}=\\ & \;=[-7.43,\;-1.26,\;-8.26,\;-0.65,\;0.51,\;0.65,\;0.85,\;0.26,\;0.35,\\ & \;{-0.34,\;0.08,\;0.14,\;0.11,\;0.21]}^{⊤} \end{array}$$

for the droplet and plate cases, respectively. Offsets are set to $\log(3\times 10^{4})$ for the droplet case and $\log(5\times 10^{5})$ for the plate case, reflecting the mean sequencing depth in cells from their respective data sets.

For the FDR, precision‐recall, and computation time evaluations, we use the statistical simulator named scDesign3 [54] to generate synthetic data sets that mirror the gene expression distributions, sequencing depth, and metadata structure of our template datasets. We then assess the ability of our method, along with competing approaches, to recover the true gene‐gene co‐expression signals. Because these data are simulated from a third‐party mechanism distinct from scCOSMiX, they provide an opportunity to assess the methods' robustness to model misspecification.

For scenarios involving comparisons to existing approaches, we consider CS‐CORE [33], the R package GJRM [38], CoCoA [31], scDECO [34], CNM [30], and sctransform‐rho [33, 55] as competing methods. Both CS‐CORE and sctransform‐rho rely on permutation testing for assessing significance, which requires specifying the number of permutations. We adopt the authors' recommended default of 100 permutations [33]. Similarly, as a Bayesian method estimated via MCMC, scDECO requires specifying the number of burn‐in and sampling iterations. To balance computational cost and estimation accuracy, we set the burn‐in to 1000 and retain 5000 post‐burn‐in samples. Special accommodations must be made for some of these methods to ensure their compatibility with the simulation scenarios being evaluated. Most notably, since none of the approaches other than GJRM account for patient‐level variability while still producing population‐level parameter estimates, we pool the data from different subjects together before applying them. Additionally, because CoCoA and CNM model their response variables as Gaussian, we pre‐process their input data using the probability integral transform as recommended by Tu et al. [31].

**Scenario 1: Power.** In the scCOSMiX model specification from Equations (5) and (6), the parameter $\tau_{1}$ encodes differential co‐expression between the two groups. So to evaluate power to detect differential co‐expression, we simulate data according to the specifications in Equations (5) and (6) with the value of $\tau_{0}$ set at 0, and we vary the value of $\tau_{1}$. At each $\tau_{1}$ value, we simulate B=500 datasets, with number of cells per patient fixed at 250, and we track the proportion of simulations in which the null hypothesis of no differential co‐expression is rejected. For scCOSMiX, this corresponds to testing $H_{0}:\tau_{1}=0$, while the competing methods are evaluated under their own respective null hypotheses.

**FIGURE 1:**
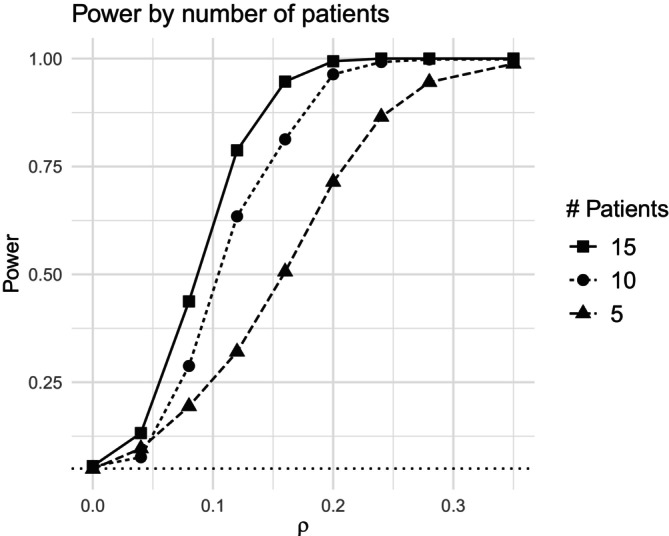
Droplet scenario: Power of scCOSMiX to detect differential co‐expression in data sets with 5,10,15 patients. Data is simulated using parameter values specified in Equation (5) based on the droplet‐based data set GSE266919. The null hypothesis being tested is that there is no differential co‐expression ($H_{0}:\tau_{1}=0$). The number of patients is set to m∈{5,10,15}, $\tau_{0}$ is set to 0, and $\tau_{1}$ is varied between 0 and 0.4. For each combination of $\tau_{1}$ and m, B=500 data sets are simulated, and power is calculated as the proportion of times in which the null hypothesis is rejected.

The first test evaluates the power of scCOSMiX to detect differential co‐expression in data sets containing m∈{5,10,15} patients. The results are displayed in Figures 1 and S1.

As shown in Figures 1 and S1, power increases with the number of patients, with a substantial gain between 5 and 10 patients and a more modest improvement between 10 and 15 patients.

**FIGURE 2:**
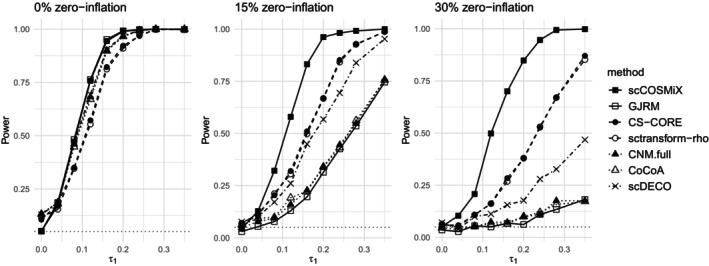
Droplet scenario: Power of scCOSMiX and competing methods to detect differential co‐expression in data sets with 0%,15%,30% zero‐inflation. Data is simulated using parameter values specified in Equation (5) based on the droplet‐based data set GSE266919. The null hypothesis being tested is that there is no differential co‐expression. The number of patients is set at m=10, $\tau_{0}$ is set to 0, $\tau_{1}$ is varied between 0 and 0.55, and $p_{1}=p_{2}\in \{0,0.15,0.30\}$. For each combination of $\tau_{1}$ and $p_{j}$, B=500 data sets are simulated, and power is calculated as the proportion of times in which the null hypothesis is rejected.

**FIGURE 3:**
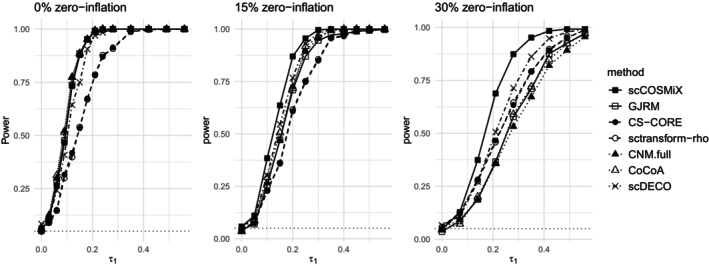
Plate scenario: Power of scCOSMiX and competing methods to detect differential co‐expression in data sets with 0%,15%,30% zero‐inflation. Data is simulated using parameter values specified in Equation (6) based on the plate‐based data set GSE108989. The null hypothesis being tested is that there is no differential co‐expression. The number of patients is set at m=10, $\tau_{0}$ is set to 0, $\tau_{1}$ is varied between 0 and 0.55, and $p_{1}=p_{2}\in \{0,0.15,0.30\}$. For each combination of $\tau_{1}$ and $p_{j}$, B=500 data sets are simulated, and power is calculated as the proportion of times in which the null hypothesis is rejected.

The second analysis evaluates the power of scCOSMiX, alongside competing methods, to detect differential co‐expression in data sets with varying amounts of zero‐inflation. In this setting, the number of patients is fixed at m=10, and the zero‐inflation is varied with $p_{1}=p_{2}\in \{0,0.15,0.30\}$. The results are displayed in Figures 2 and 3.

In the 0% zero‐inflation setting from Figures 2 and 3, we observe inflated type I error rates in all methods that do not account for within‐patient variability, especially in droplet‐based data. This is a well‐documented consequence of treating dependent observations as independent in scRNA‐seq data [25, 26, 27]. As zero‐inflation increases, type I error rates fall back toward the nominal level, as the greater degree of model misspecification resulting from not accounting for zero‐inflation leads to fewer rejections overall and thus fewer false positives. Additionally, it can be seen that as the amount of zero‐inflation in the data increases, scCOSMiX becomes increasingly more powerful than competing methods. The trend is less pronounced in the plate‐based data than in the droplet‐based data because the plate‐based data were generated with higher over‐dispersion values. High over‐dispersion values are typically observed in plate‐based data, and they reduce the impact of zero‐inflation, enabling misspecified distributions to capture the overall shape of the data without explicitly modeling excess zeros. A more detailed analysis of this phenomenon is provided in Appendix 2.

**Scenario 2: Coverage.** To assess the effectiveness of scCOSMiX parameter estimation, we simulate data sets using the parameters specified in (5) and (6) and calculate the average mean squared error (MSE), average mean bias error (MBE), the proportion of times that the true parameter value falls within its confidence interval (coverage), and also the average width of each confidence interval (CI Width). We carry out this study using m∈{5,10,15} patients, with the number of cells per patient set at 200, and the number of iterations at B=500. The results are displayed in Tables 1 and S1.

**TABLE 1:** Droplet scenario: Estimation performance for scCOSMiX, evaluated on data sets simulated with parameter values specified in Equation (5) based on the droplet‐based data set GSE266919. Number of patients is set to m∈{5,10,15}, and for each value of m, B=500 datasets are simulated, and estimates and confidence intervals are obtained for each parameter.

	5 Patients				10 Patients				15 Patients			
Parameter	Coverage	MSE	MBE	CI Width	Coverage	MSE	MBE	CI Width	Coverage	MSE	MBE	CI Width
$\beta_{01}$	0.882	0.008	0.071	0.479	0.926	0.004	0.048	0.241	0.938	0.002	0.039	0.195
$\beta_{11}$	0.918	0.016	0.102	0.632	0.956	0.007	0.068	0.346	0.948	0.005	0.057	0.281
$\beta_{02}$	0.894	0.008	0.071	0.602	0.922	0.004	0.049	0.239	0.934	0.003	0.041	0.202
$\beta_{12}$	0.896	0.016	0.100	0.779	0.924	0.008	0.069	0.342	0.952	0.005	0.057	0.283
$\alpha_{01}$	0.956	0.005	0.056	0.280	0.950	0.002	0.040	0.198	0.946	0.002	0.034	0.162
$\alpha_{11}$	0.968	0.010	0.078	0.411	0.950	0.005	0.058	0.292	0.940	0.004	0.051	0.239
$\alpha_{02}$	0.956	0.005	0.057	0.280	0.938	0.003	0.044	0.198	0.960	0.002	0.033	0.162
$\alpha_{12}$	0.942	0.011	0.084	0.399	0.948	0.005	0.060	0.283	0.976	0.003	0.044	0.231
$\tau_{0}$	0.946	0.003	0.045	0.325	0.932	0.001	0.030	0.154	0.934	0.001	0.026	0.125
$\tau_{1}$	0.958	0.006	0.061	0.458	0.960	0.003	0.042	0.219	0.966	0.002	0.035	0.176
$p_{01}$	0.950	0.000	0.009	0.052	0.972	0.000	0.006	0.033	0.950	0.000	0.005	0.027
$p_{11}$	0.928	0.000	0.006	0.167	0.938	0.000	0.005	0.085	0.942	0.000	0.004	0.041
$p_{02}$	0.958	0.000	0.005	0.084	0.964	0.000	0.004	0.034	0.950	0.000	0.003	0.017
$p_{12}$	0.924	0.000	0.004	0.150	0.940	0.000	0.003	0.048	0.948	0.000	0.002	0.027

As demonstrated in Tables 1 and S1, the parameters are accurately estimated, as reflected by proper coverage rates and low MSE, MBE, and confidence interval widths. Estimation performance improves with the number of patients, with coverage increasing and error metrics decreasing as number of patients increases.

**Scenario 3: Robustness.** To assess the effectiveness of scCOSMiX parameter estimation in a scenario without zero‐inflation, we simulate B=500 samples using the same parameters as in the previous section except this time with zero‐inflation set to zero. Then we calculate the MSE, MBE, coverage, and CI Width as done in Scenario 2. Once again, we use 5,10, and 15 patients, with the number of cells per patient set at n=200, and the number of iterations at B=500. The results are displayed in Tables 2 and S2.

**TABLE 2:** Droplet scenario: Robustness performance for scCOSMiX, evaluated on datasets simulated with parameter values specified in Equation (5) based on the droplet‐based dataset GSE266919, with $p_{1}=p_{2}=0$. Number of patients is set to m∈{5,10,15}, and for each value of m, B=500 datasets are simulated, and estimates and confidence intervals are obtained for each non‐zero‐inflation parameter.

	5 Patients				10 Patients				15 Patients			
Parameter	Coverage	MSE	MBE	CI Width	Coverage	MSE	MBE	CI Width	Coverage	MSE	MBE	CI Width
$\beta_{01}$	0.894	0.010	0.080	0.398	0.918	0.006	0.060	0.285	0.939	0.004	0.050	0.240
$\beta_{11}$	0.906	0.021	0.120	0.591	0.934	0.012	0.085	0.468	0.945	0.008	0.070	0.344
$\beta_{02}$	0.898	0.011	0.086	0.400	0.942	0.005	0.057	0.372	0.947	0.004	0.048	0.243
$\beta_{12}$	0.918	0.023	0.121	0.575	0.938	0.010	0.081	0.493	0.954	0.007	0.069	0.343
$\alpha_{01}$	0.948	0.006	0.062	0.296	0.934	0.003	0.046	0.210	0.937	0.002	0.037	0.172
$\alpha_{11}$	0.956	0.013	0.090	0.446	0.952	0.007	0.064	0.316	0.952	0.004	0.051	0.259
$\alpha_{02}$	0.932	0.006	0.063	0.300	0.936	0.003	0.046	0.215	0.928	0.002	0.037	0.176
$\alpha_{12}$	0.958	0.012	0.088	0.438	0.944	0.006	0.063	0.312	0.958	0.004	0.049	0.256
$\tau_{0}$	0.950	0.003	0.040	0.224	0.962	0.001	0.028	0.150	0.954	0.001	0.023	0.119
$\tau_{1}$	0.966	0.005	0.057	0.320	0.964	0.002	0.039	0.216	0.952	0.002	0.033	0.170

In Tables 2 and S2, we observe that estimation performance remains consistent in the absence of zero inflation. In both droplet‐ and plate‐based settings, coverage is close to nominal, and MSE, MBE, and CI width improve as the number of patients increases. Overall, results are similar to those observed in the zero‐inflated setting, indicating that scCOSMiX is robust even if the data are not zero‐inflated. An additional robustness study is conducted for the case where zero inflation is present in only one marginal. The results of this study, presented in Tables S3 and S4, demonstrate that scCOSMiX maintains robust performance even under asymmetric zero inflation.

**FIGURE 4:**
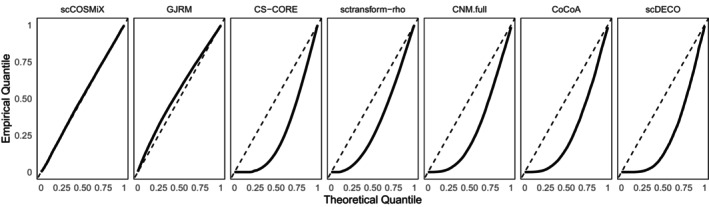
Droplet scenario: Q‐Q plots for the distribution of p‐values for differential co‐expression, based on datasets generated using scDesign3 with the droplet‐based dataset GSE266919 as a template. For each of the B=100 simulated replicates, 1225 gene pairs are simulated under the null hypothesis. Each method is then applied to each replicate dataset, and p‐values are computed for all 1225 gene pairs. The black curves show the empirical distribution of p‐values, averaged across replicates for each method. The dashed line represents the expected Unif(0,1) distribution under the null.

**Scenario 4: FDR.** To evaluate FDR, we use scDesign3 to generate B=100 simulated replicates of our template datasets from Section 2.1. These replicates are designed to mirror the characteristics of the original datasets. A subset of 1225 gene pairs is selected and their differential co‐expression is manually set to 0. Then, for each of the B=100 datasets, we apply scCOSMiX and competing methods to the 1225 gene pairs and record the resulting 1225 differential co‐expression p‐values for each method. Since the data were simulated under the null hypothesis for those gene pairs, the distribution of p‐values under the null should follow Unif(0,1). The resulting Q‐Q plots for each method, averaged over the B=100 replicates, are presented in Figures 4 and S2.

In the droplet‐based setting (Figure 4), scCOSMiX produces well‐calibrated p‐values under the null, and GJRM performs similarly, though with slight conservativeness. The remaining methods exhibit an excess of small p‐values indicating anti‐conservative behavior. This behavior matches the inflated type I errors seen in the 0% zero‐inflation droplet‐based power study, and is a well‐studied consequence of ignoring within‐patient variability. In the plate‐based setting (Figure S2), p‐value calibration improves across all methods, matching the proper type I error rates observed in Figure 3.

**Scenario 5: Precision‐Recall** To evaluate precision‐recall performance, we generate data under the same framework as in the FDR simulation, except we manually set 140 of the 1225 gene pairs to have differential co‐expression of 0.20. The remaining gene pairs have their differential co‐expression parameters kept at 0, and we proceed to simulate the data sets using scDesign3. The precision and recall, averaged over the B=100 data sets, are plotted in Figures 4 and S2.

**FIGURE 5:**
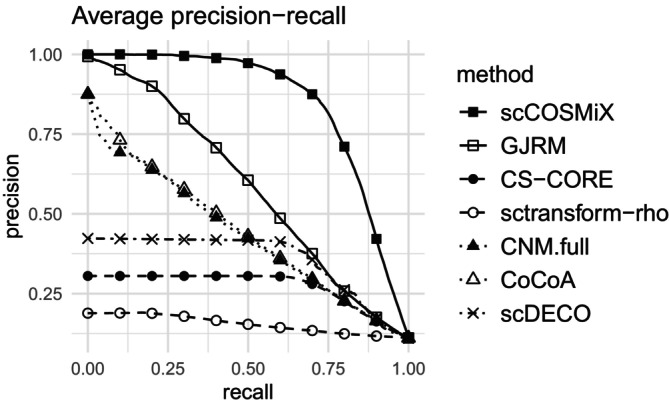
Droplet scenario: Precision‐recall curves for differential co‐expression, evaluated on data simulated based on data sets generated using scDesign3 with the droplet‐based data set GSE266919 as a template. B=100 data sets were simulated using scDesign3 based on a subset of 1225 gene pairs from the template data set. 140 of the gene pairs were randomly selected and manually given a differential co‐expression of 0.20 while the remaining 1085 gene pairs have no differential co‐expression.

In Figures 5 and S3, scCOSMiX achieves the highest precision across the entire recall range. At a certain level of recall, CS‐CORE and sctransform‐rho both reach a plateau where precision cannot increase further. This is due to their permutation‐based approach, where the granularity of their p‐values is constrained by the number of permutations performed. Mirroring the findings from the power study, precision‐recall performance in all methods is higher in the plate‐based setting than in the droplet‐based setting. CoCoA and CNM show particular improvement between the two settings, as their Gaussian pre‐processing transformation is more effective on the high over‐dispersion, high mean expressions that characterize plate‐based data. GJRM performs well in the droplet‐based setting and moderately well in the plate‐based setting. This mirrors the findings of the power and FDR studies, where accounting for within‐patient variability proved more important in the low over‐dispersion, low mean environment of droplet‐based data.

**Scenario 6: Computation Time** We recorded the average computation time (in seconds) required to fit a single gene pair from the data simulated under the scenario from the previous section. To calculate this, we simulate a data set from the droplet‐based and plate‐based settings using scDesign3, and we select 1225 gene pairs to use in the evaluation. We then fit each method, and divide their total computation time by 1225 to obtain the average computation time to fit a single gene pair. Computations are performed on an Intel Xeon E5‐2680 v4 CPU (2.40 GHz, 8 cores) with 128 GB RAM. Results are presented in Table 3.

**TABLE 3:** Computation time (in seconds) for fitting a single gene pair. Times are averages over 1225 gene pairs from data simulated using scDesign3 with droplet‐based data set GSE266919 and plate‐based data set GSE108989 as templates. Computations performed on an Intel Xeon E5‐2680 v4 CPU (2.40 GHz, 8 cores) with 128 GB RAM.

	scCOSMiX	CS‐CORE*	sctransform‐rho*	CoCoA	CNM	GJRM	scDECO
Droplet	163.03	0.02	0.15	0.47	0.54	140.63	477.43
Plate	271.58	0.02	0.12	0.39	0.38	329.14	348.28

*Note:* * 100 permutations.

The computation time required for CS‐CORE, sctransform‐rho, and scDECO depends on the number of permutation iterations or the number of MCMC iterations. As shown in Table 3, computational time varies considerably across the different methods. The Bayesian method scDECO proves to be the most computationally intensive, followed by the methods that account for within‐patient variability (scCOSMiX and GJRM). Methods that assume normality (CoCoA and CNM) rank next in speed, while methods that do not rely on likelihood‐based estimation (CS‐CORE and sctransform‐rho) seem to be fast with 100 permutation iterations. However, the time required for CS‐CORE and sctransform‐rho depends on the number of permutation iterations specified, and the number of permutation iterations influences the precision of the p‐value calculation. The computational efficiency of these seemingly faster methods comes at the cost of estimation bias and modeling restrictions. The Gaussian transformation used by CoCoA and CNM is ineffective when applied to sparse or over‐dispersed count data, while the assumption of identically distributed cells within each cell type renders CS‐CORE and sctransform‐rho incapable of modeling covariate effects in the mean, over‐dispersion, and co‐expression parameters. Furthermore, none of the fast methods can be directly applied to multi‐patient data sets without discarding patient information or aggregating cells across individuals. In contrast, scCOSMiX accommodates covariate effects, patient‐level variation, and potential zero‐inflation in a unified likelihood‐based framework, enabling flexible and interpretable modeling of gene expression and co‐expression across complex experimental designs.

## 4 Experimental Data Analyses

### 4.1 Application to the TNBC Dataset

We apply scCOSMiX to the droplet‐based TNBC data set GSE266919 described in Section 2.1, focusing on myeloid cells from 8 TNBC patients who responded to treatment with nab‐paclitaxel or nab‐paclitaxel plus atezolizumab. We investigate the changes in gene‐gene co‐expression patterns in myeloid cells between pre‐ and post‐treatment. This analysis aims to uncover changes in transcriptional interactions associated with the treatments.

To carry out the analysis, we define the fixed‐ and random‐effect structures for each parameter $\theta \in \{\mu_{1},\mu_{2},\sigma_{1},\sigma_{2},\rho ,p_{1},p_{2}\}$ according to the model framework described in Section 2.2. For each θ, we define the fixed‐effects design matrix $X_{i,\theta }$ to consist of an intercept and an indicator for post‐treatment status, in order to allow systematic shifts between pre‐ and post‐treatment conditions to be modeled for each parameter. We specify the random‐effects design matrix $Z_{i,\theta }$, the associated random intercept vector $u_{\theta }$, and the variance structure $\sum_{\theta }$ as follows. For the dropout probabilities $\left(\theta \in \{p_{1},p_{2}\}\right)$, $Z_{i,\theta }$ encodes random intercepts at the patient level, with a shared variance component across the eight patients $\left(\text{diag}\left(\sum_{\theta }\right)=\sigma_{\theta }^{2}1_{8}\right)$. For the mean and co‐expression parameters $\left(\theta \in \{\mu_{1},\mu_{2},\rho \}\right)$, $Z_{i,\theta }$ encodes random intercepts at the patient‐by‐timepoint level, with separate variance components for the pre‐ and post‐treatment groups $\left(\text{diag}\left(\sum_{\theta }\right)=(\sigma_{\theta ,\text{pre}}^{2}1_{8};\sigma_{\theta ,\text{post}}^{2}1_{8})\right)$. For the dispersion parameters $\left(\theta \in \{\sigma_{1},\sigma_{2}\}\right)$, following the rationale in Malfait et al. [26], we set $Z_{i,\theta }=0$ and $u_{\theta }=0$.

**FIGURE 6:**
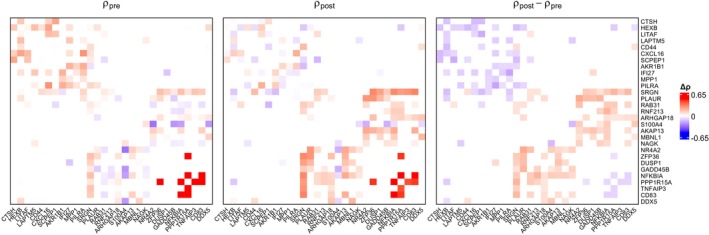
Droplet scenario: Heatmaps of gene‐gene correlation estimates for selected genes in pre‐treatment and post‐treatment cells from droplet‐based TNBC data set GSE266919, with an additional heatmap showing co‐expression changes between the two conditions.

Defining the fixed‐ and random‐effects structures in this way enables systematic modeling of timepoint effects while accounting for subject‐level and longitudinal heterogeneity in the dropout, mean, and co‐expression parameters. Before carrying out the analysis, we filter out mitochondrial, housekeeping (Eisenberg and Levanon [56]), and non‐protein‐coding (Durinck et al. [57]) genes from consideration. We further remove genes if they have more than 70% of their expression counts equal to 0 or if their 97.5% upper quantile is less than 4. After this pre‐processing, 251 genes (31 375 gene pairs) remain in the analysis. For each pair of genes, we fit the above model and obtain a p‐value for the $\tau_{1}$ parameter. The Benjamini‐Hochberg (BH) correction [58] is then applied to those 31 375 p‐values to adjust for multiple testing and control the false discovery rate. After the correction, 61 gene pairs were found to be significant. The top 25 gene pairs in terms of |Δρ| are displayed in Table 4. In Figure 6, we present heatmaps of the pre‐treatment and post‐treatment co‐expression for selected gene pairs, along with a heatmap illustrating the co‐expression changes between the two conditions.

**TABLE 4:** Droplet scenario: Top table of co‐expression differences between pre‐treatment and post‐treatment cells from droplet‐based TNBC dataset GSE266919.

#	Gene 1	Gene 2	ρ(pre)	ρ(post)	|Δρ|
1	GPNMB	SRGN	−0.001	−0.279	0.277
2	CDKN1A	SRGN	0.045	0.251	0.205
3	HSPA1A	C1QB	0.093	−0.112	0.205
4	GPNMB	KLF6	−0.034	−0.236	0.203
5	GPNMB	SGK1	0.063	−0.137	0.200
6	IFI27	PILRA	0.148	−0.050	0.198
7	APOE	SRGN	0.021	−0.177	0.197
8	SLC16A3	SH3BGRL3	0.013	0.209	0.196
9	RNF213	NFKBIA	−0.065	0.127	0.193
10	SRGN	PPP1R15A	0.068	0.260	0.192
11	ISG15	NR4A2	−0.150	0.037	0.187
12	LITAF	SCPEP1	0.155	−0.026	0.181
13	GM2A	SRGN	0.045	−0.134	0.178
14	LIPA	SRGN	0.002	−0.173	0.175
15	CTSS	SGK1	0.027	−0.144	0.171
16	CCL4	FABP5	−0.059	−0.226	0.167
17	LAIR1	C1QB	0.234	0.067	0.167
18	NFKBIA	CCL4	0.549	0.383	0.166
19	CMTM6	CTSS	0.078	−0.086	0.164
20	CD53	FCGRT	0.143	−0.021	0.164
21	IFI6	NR4A2	−0.311	−0.148	0.164
22	CTSL	PILRA	0.125	−0.037	0.161
23	NR4A2	RAB31	−0.066	0.095	0.161
24	APOC1	MCL1	−0.131	−0.292	0.161
25	PRNP	FOSB	−0.007	0.154	0.161

The heatmaps in Figure 6 illustrate some of the co‐expression changes induced within myeloid cells by treatment with nab‐paclitaxel. Co‐expression changes in gene pairs such as *SRGN*‐*RAB31* and *CXCL16*‐*HEXB* reflect the underlying reprogramming of myeloid regulatory networks described in the original study of this data set [39]. In prior research, *SRGN* has been shown to regulate the packaging and secretion of inflammatory cytokines such as TNF‐α [59], while *RAB31* has been identified as a contributor to early‐stage immune signaling and intracellular transport of signaling molecules [60]. The increased co‐expression of these two genes following treatment is consistent with the original study's findings that nab‐paclitaxel promotes inflammatory macrophage states with elevated immune activity. In contrast, *CXCL16*, which promotes monocyte recruitment and supports stromal activation in triple‐negative breast cancer [61], and *HEXB*, a marker of metabolically active tumor‐associated macrophages involved in glycolytic signaling [62], show decreased co‐expression – reflecting the disruption of suppressive myeloid states and the shift toward inflammatory reprogramming reported in the original study.

### 4.2 Application to the CRC Data Set

We apply the scCOSMiX model to the plate‐based CRC data set GSE108989 described in Section 2.1, focusing on tumor‐derived CD4 and CD8 T cells from 11 colorectal cancer patients. In their original study, Zhang et al. [40] reported that tumor‐infiltrating lymphocytes are highly heterogeneous with respect to gene expression profiles and clonal expansion patterns, with CD4 and CD8 T cells exhibiting markedly different transcriptional programs. We use scCOSMiX to examine whether these lineage‐specific transcriptional differences are reflected in distinct patterns of gene‐gene co‐expression between CD4 and CD8 T cells.

To this end, we apply the same fixed‐effect, random‐effect, and offset specification as in Section 4.1, but with the factor of interest changed from timepoint to cell type. The gene‐filtering process also remains the same, except this time we include an additional filter on the variance of the gene expression counts. We remove a gene if its normalized gene expression in either of the CD4 or CD8 cell types has standard deviation less than 0.4 or if the absolute difference between the CD4 and CD8 standard deviations is less than 0.1. The normalization is carried out using the methods from Hafemeister et al. [55] and is used to ensure the variance calculated is not due to sequencing depth effects. After filtering, 276 genes (37 950 gene pairs) remain for analysis. For each pair of genes, we fit the scCOSMiX model and obtain a p‐value for the $\tau_{1}$ parameter. The BH correction is then applied to those 37 950 p‐values to adjust for multiple testing and control the false discovery rate. After the correction, 548 gene pairs were found to be significant. The top 25 gene pairs in terms of |Δρ| are displayed in Table S5. In Figure S4, we present heatmaps of the CD4 and CD8 co‐expression for selected gene pairs, along with a heatmap illustrating the co‐expression changes between the two lineages.

The heatmaps in Figure S4 illustrate some of the co‐expression differences found between CD4 and CD8 T cells in our analysis. Decreased co‐expression in gene pairs such as *CD28*‐*RHOG* and *MAP2K3*‐*TFRC* reflects the transcriptional disorganization and functional exhaustion of CD8 cells described in the original study of the data set [40]. In prior research, *CD28* has been found to be a key co‐stimulatory receptor for T cell activation, particularly active in CD4 subsets [63], while *RHOG* has been identified as a modulator of T cell activation by dampening TCR signaling in later stages of the response [64]. Similarly, *MAP2K3* contributes to stress and survival signaling in colorectal cancer [65], and *TFRC* supports iron uptake and metabolic activity required for proliferation and effector function [66]. The reduced co‐expression of these two gene pairs in CD8 cells indicates impaired coordination of activation, signaling, and metabolic programs, consistent with the original study's finding that tumor‐infiltrating CD8 T cells are more exhausted, dysfunctional, and transcriptionally fragmented than CD4 T cells.

## 5 Discussion

In recent years, the proliferation of multi‐patient scRNA‐seq studies has created rich opportunities to analyze dynamic gene‐gene interaction changes across cell types, developmental stages, treatment conditions, and other biological contexts. However, such analyses involve substantial methodological challenges, including the hierarchical structure inherent in sampling multiple cells from each patient, the count‐based nature of gene expression measurements with protocol‐dependent distributional properties, and the need to model covariate‐dependent changes in both the marginal expression levels and the gene‐gene dependence structure. To address these challenges, in this paper we proposed scCOSMiX, a flexible zero‐inflated bivariate copula mixed‐effects model for analyzing differential co‐expression in multi‐patient scRNA‐seq data appropriate for both plate‐based and droplet‐based protocols.

We carried out simulation studies based on scenarios from both droplet‐ and plate‐based data sets, evaluating coverage, robustness, power, FDR control, precision‐recall, and computation time. As demonstrated by our simulations and experimental data analyses, the algorithm can flexibly estimate parameter values generated by various experimental protocols. In the coverage and robustness studies, scCOSMiX maintained proper coverage rate and low MSE for all parameters, even when fit to data generated without zero inflation. It performed similarly well in the power studies, where it showed high power on data simulated with 0%,15%, and 30% zero‐inflation. In the computation time study (Table 3), we found the mixed effects framework of scCOSMiX incurred a greater computational burden compared to methods treating all cells as independent, but this additional computational burden was offset by superior performance in the FDR control, precision‐recall, and power studies.

The scCOSMiX framework provides flexibility for researchers to specify both the marginal distributions and the copula to accommodate a wide range of data types. In this paper, we used a Gaussian copula with negative binomial marginals to capture gene co‐expression in scRNA‐seq data, but the model can be tailored to suit data from contexts beyond gene expression, including multi‐omics and microbiome studies. For example, by setting the first marginal as log‐normal and the second marginal as negative binomial, scientists could analyze whether the correlation between protein abundance and chromatin accessibility is in some way modulated by the cell type. Alternatively, to analyze microbial relative abundances, which often exhibit non‐elliptical dependence structures [67], the user could specify a Frank copula in place of a Gaussian copula. To ensure that these user‐specified marginal and copula choices provide a good fit to the data, model fit can be evaluated using randomized quantile residuals [68, 69] and corrected conditional AIC [70].

For a typical scRNA‐seq data set, there are several search heuristics that can be applied using scCOSMiX to identify differential co‐expression gene pairs: (i) candidate gene sets or pathways (ii) genome‐wide screening. Depending on the number of genes in the data set and the computing resources available, one may employ pre‐screening measures such as ζ, introduced by Yu (2018) [71], fastLA, proposed by Gunderson and Ho (2014) [72], or CS‐CORE, developed by Su et al. (2023) [33].

Although the simulation studies and real data analyses in this paper involved only a two‐level categorical covariate (pre‐ vs. post‐treatment in the droplet case and CD4 vs. CD8 in the plate case), the scCOSMiX framework can be used for modeling scenarios involving continuous covariates, categorical covariates with multiple levels, and interactions of covariates. The model‐based nature of scCOSMiX enables testing of complex hypotheses, such as whether the co‐expression of two genes changes with the expression level of a third gene, and whether that change is different in one cell type versus another. This flexibility sets it apart from existing methods such as CS‐CORE, which does not permit continuous covariates or categorical covariates with more than two levels; CoCoA, which does not incorporate covariate effects in the marginal parameters, and CNM, which does not allow more than one covariate in the model.

We conducted two real data analyses: the first on droplet‐based TNBC data set GSE266919, and the second on plate‐based CRC data set GSE108989. In the TNBC data set, we investigated co‐expression changes from pre‐ to post‐treatment in myeloid cells from 8 patients treated with nab‐paclitaxel or nab‐paclitaxel plus atezolizumab. In the CRC data set, we analyzed co‐expression differences between CD4 and CD8 T cells in tumor tissue from 11 CRC patients. For both analyses, we presented top tables featuring the 25 gene pairs with the largest differential co‐expression, and we plotted heat maps for a select number of genes to illustrate the phenomenon of co‐expression visually. The results of these analyses suggested plausible biological hypotheses that matched prior research on the data sets.

## Conflicts of Interest

D.T. contributed to this article as an employee of the University of Pennsylvania and the views expressed do not necessarily represent the views of Regeneron Pharmaceuticals Inc.

## Supporting information




**Data S1.** Additional supporting information, including additional plots and tables referenced in the text, a Kullback‐Leibler divergence study, and derivations of the gradient and hessian may be found in the online version of the article at the publisher's website.

## Data Availability

Data for the real data analyses may be accessed at https://www.ncbi.nlm.nih.gov/geo/query/acc.cgi?acc=GSE266919 and https://www.ncbi.nlm.nih.gov/geo/query/acc.cgi?acc=GSE108989. Data pre‐processing code as well as the processed data method may be found at https://github.com/YenYiHo‐Lab/scCOSMiX.
