# Supplementary material for: scCOSMIX: A Mixed‐Effects Framework for Differential Coexpression and Transcriptional Interactions Modeling in Single‐Cell RNA‐Seq
Source: Stat Med. 2025 Aug 7;44(18-19):e70213. doi: 10.1002/sim.70213 (PMC12330344; doi:10.1002/sim.70213)
Supplement: Supplementary file 1 — Data S1. Additional supporting information, including additional plots and tables referenced in the text, a Kullback‐Leibler divergence study, and derivations of the gradient and hessian may be found in the online version of the article at the publisher's website. [file SIM-44-0-s001.zip › Bussing_FigS2.pdf]

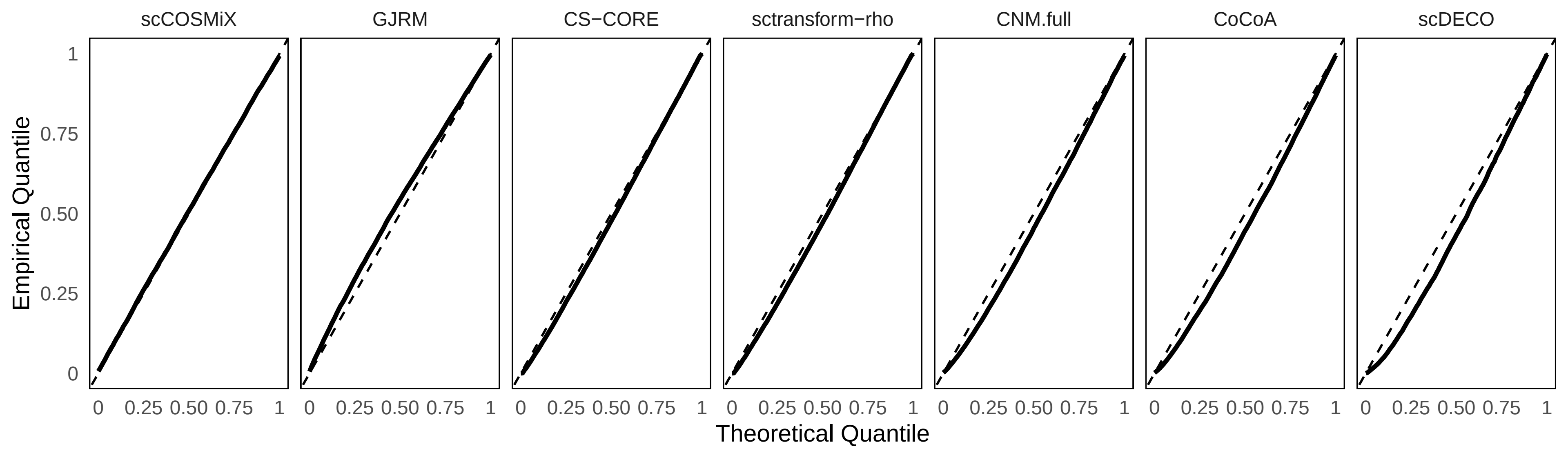
scCOSMiX
GJRM
CS−CORE
sctransform−rho
CNM.full
CoCoA
scDECO
Empirical Quantile
0
0.25
0.50
0.75
1
Theoretical Quantile
