# Supplementary material for: scCOSMIX: A Mixed‐Effects Framework for Differential Coexpression and Transcriptional Interactions Modeling in Single‐Cell RNA‐Seq
Source: Stat Med. 2025 Aug 7;44(18-19):e70213. doi: 10.1002/sim.70213 (PMC12330344; doi:10.1002/sim.70213)
Supplement: Supplementary file 1 — Data S1. Additional supporting information, including additional plots and tables referenced in the text, a Kullback‐Leibler divergence study, and derivations of the gradient and hessian may be found in the online version of the article at the publisher's website. [file SIM-44-0-s001.zip › scCOSMiX_Revision_1_Supplementary.pdf]

Supplementary Material for scCOSMiX

1 | ADDITIONAL TABLES AND PLOTS

[Figure 1 about here.]

[Table 1 about here.]

[Table 2 about here.]

[Table 3 about here.]

[Table 4 about here.]

[Figure 2 about here.]

[Figure 3 about here.]

[Table 5 about here.]

[Figure 4 about here.]

2 | KULLBACK-LEIBLER DIVERGENCE STUDY

The Kullback-Leibler divergence[1] between the true scCOSMiX model and the closest approximation within the misspecified model class from Equation 1 in Section 2.2 of the main manuscript is given by

$$\mathrm{KL} = \min_{\boldsymbol{\vartheta}} \sum_{(y_1,y_2)\in\mathbb{N}_0^2} f(y_1, y_2; \boldsymbol{\theta}) \log\left(\frac{f(y_1, y_2; \boldsymbol{\theta})}{f^*(y_1, y_2; \boldsymbol{\vartheta})}\right),$$

where f and f^* denote the joint probability mass functions under the true and misspecified models, respectively. The pseudotrue parameter $\boldsymbol{\theta}^*$ is the value of $\boldsymbol{\vartheta}$ that achieves this minimum:

$$\boldsymbol{\theta}^* = \arg\min_{\boldsymbol{\vartheta}} \sum_{(y_1,y_2)\in\mathbb{N}_0^2} f(y_1, y_2; \boldsymbol{\theta}) \log\left(\frac{f(y_1, y_2; \boldsymbol{\theta})}{f^*(y_1, y_2; \boldsymbol{\vartheta})}\right).$$

To illustrate this concept, we provide some specific examples before presenting the larger study. In Table S6, the pseudotrue $\boldsymbol{\theta}^*$ is presented for a case where true $\boldsymbol{\theta}$ has small σ_1, σ_2 (low over-dispersion).

[Table 6 about here.]

We note that in its attempt to capture the true distribution, the misspecified model has adopted smaller mean and dependence parameters, while its over-dispersion parameters are larger than their true counterparts. To see the difference between the true pmf $f(y_1, y_2\ ;\ \boldsymbol{\theta})$ and the corresponding misspecified pmf $f^*(y_1, y_2\ ;\boldsymbol{\theta}^*)$ more clearly, we plot them (log-transformed) side by side in Figure S5.

[Figure 5 about here.]

It is apparent that the misspecified model cannot effectively capture the shape of the true distribution, and the relatively large KL divergence value of 0.16 reflects this.

For the next example, we keep all the parameters of $\boldsymbol{\theta}$ the same as before, except σ_1, σ_2 are made larger (high over-dispersion). The pseudotrue for this case is presented in Table S7.

[Table 7 about here.]

As in the low over-dispersion case, we observe a relative attenuation of the mean parameters and inflation of the over-dispersion parameters. However, the dependence parameter is notably less shrunken in this case than in the low over-dispersion case. In Figure S6, we observe that the shapes of the two distributions are much more similar visually than in the previous example. Furthermore, the KL divergence of 0.06 is significantly smaller than in the low over-dispersion case.

[Figure 6 about here.]

These two examples suggest that non-zero-inflated models can better approximate the scCOSMiX model in cases where over-dispersion is high. In order to investigate this idea more thoroughly, we compute the KL divergence and its corresponding pseudotrue for a range of different values for $\boldsymbol{\theta}$. Specifically, we have $\boldsymbol{\theta} = (\mu_1, \mu_2, \sigma_1, \sigma_2, \rho, p_1, p_2) = (\mu, \mu, \sigma, \sigma, 0.5, 0.3, 0.3)$, and we vary μ, σ over a grid.

[Figure 7 about here.]

[Figure 8 about here.]

3 | LIKELIHOOD AND DERIVATIVES

3.1 | Bivariate Copulas

A bivariate copula is a 2-dimensional joint conditional distribution function (cdf) $C(u_1, u_2)$ whose marginals follow a standard uniform distribution[2]. Sklar's theorem[3] applied to the bivariate case states that any bivariate joint cdf G with marginals F_1, F_2 can be expressed as

$$G(x_1, x_2) = C\left(F_1(x_1), F_2(x_2)\right),$$

where C is some bivariate copula. Conversely, any bivariate copula C can be applied to any pair of marginal cdfs F_1, F_2 to create a bivariate joint cdf with marginals F_1, F_2[4]. The utility of this result is that it allows us to specify marginal distributions of our choosing, and then impart a dependence between them through a copula. For many popular copulas, the dependence structure between the marginals is parameterized by a single parameter. These include the Clayton, Joe, Gumbel, Frank, and Gaussian copulas.

Let Y_1 and Y_2 be random variables with cdfs $F_{m_1}(\cdot; \boldsymbol{\theta}_1)$ and $F_{m_2}(\cdot; \boldsymbol{\theta}_2)$ respectively, and let $C(u_1, u_2;\ \rho)$ be a copula whose dependence structure is parameterized through a single parameter ρ. Then by the converse of Sklar's theorem, C induces a bivariate joint cdf G on (Y_1, Y_2) which can be expressed as:

$$G\left(y_1, y_2;\ \boldsymbol{\theta}_1, \boldsymbol{\theta}_2, \rho\right) = C\left(F_{m_1}(y_1;\ \boldsymbol{\theta}_1),\ F_{m_2}(y_2;\ \boldsymbol{\theta}_2)\right).$$

If F_{m_1} and F_{m_2} are discrete distributions, then we can use the following property

$$\begin{aligned} P\left(Y_1 = y_1, Y_2 = y_2\right) =& P\left(Y_1 \le y_1, Y_2 \le y_2\right) \\ &- P\left(Y_1 \le y_1 - 1, Y_2 \le y_2\right) \\ &- P\left(Y_1 \le y_1, Y_2 \le y_2 - 1\right) \\ &+ P\left(Y_1 \le y_1 - 1, Y_2 \le y_2 - 1\right), \end{aligned}$$

to write the joint probability mass function (pmf) as:

$$\begin{aligned} g\left(y_1, y_2;\ \boldsymbol{\theta}_1, \boldsymbol{\theta}_2, \rho\right) =& G\left(y_1, y_2;\ \boldsymbol{\theta}_1, \boldsymbol{\theta}_2, \rho\right) + \\ & - G\left(y_1 - 1, y_2;\ \boldsymbol{\theta}_1, \boldsymbol{\theta}_2, \rho\right) + \\ & - G\left(y_1, y_2 - 1;\ \boldsymbol{\theta}_1, \boldsymbol{\theta}_2, \rho\right) + \\ & + G\left(y_1 - 1, y_2 - 1;\ \boldsymbol{\theta}_1, \boldsymbol{\theta}_2, \rho\right). \end{aligned} \tag{1}$$

If F_{m_1} and F_{m_2} are continuous distributions, then the joint probability density function (pdf) is obtained by taking the mixed derivative of $G\left(y_1, y_2;\ \boldsymbol{\theta}_1, \boldsymbol{\theta}_2, \rho\right)$ with respect to y_1 and y_2 as follows:

$$\begin{aligned} g(y_1, y_2;\ \boldsymbol{\theta}_1, \boldsymbol{\theta}_2, \rho) &= \frac{\partial^2}{\partial y_1 \partial y_2} G\left(y_1, y_2;\ \boldsymbol{\theta}_1, \boldsymbol{\theta}_2, \rho\right) = \\ &= \left[\frac{\partial^2 C\left(F_{m_1}(y_1;\ \boldsymbol{\theta}_1), F_{m_2}(y_2;\ \boldsymbol{\theta}_2)\right)}{\partial F_{m_1}(y_1;\ \boldsymbol{\theta}_1) \partial F_{m_2}(y_2;\ \boldsymbol{\theta}_2)}\right] \frac{\partial F_{m_1}(y_1;\ \boldsymbol{\theta}_1)}{\partial y_1} \frac{\partial F_{m_2}(y_2;\ \boldsymbol{\theta}_2)}{\partial y_2} = \\ &= \left[\frac{\partial^2 C\left(F_{m_1}(y_1;\ \boldsymbol{\theta}_1), F_{m_2}(y_2;\ \boldsymbol{\theta}_2)\right)}{\partial F_{m_1}(y_1;\ \boldsymbol{\theta}_1) \partial F_{m_2}(y_2;\ \boldsymbol{\theta}_2)}\right] f_{m_1}(y_1;\ \boldsymbol{\theta}_1) f_{m_2}(y_2;\ \boldsymbol{\theta}_2) \end{aligned} \tag{2}$$

The Gaussian copula is a popular choice in the literature for analyzing scRNA-seq data[5,6,7,8], and its association parameter ρ, being the correlation coefficient of a multivariate Gaussian distribution, is readily interpretable. The bivariate Gaussian copula is defined as

$$C(u_1, u_2;\ \rho) = \Phi_2\left(\Phi^{-1}(u_1),\ \Phi^{-1}(u_2);\ \rho\right),$$

[9] where Φ_2 is the cdf of a standard bivariate Gaussian random vector with correlation coefficient ρ, and Φ^{-1} is the standard univariate Gaussian quantile function. Thus, applying the Gaussian copula to a pair of random variables Y_1 and Y_2 (with marginal cdfs F_{m_1} and F_{m_2}) induces the following bivariate joint cdf:

$$G\left(y_1, y_2;\ \boldsymbol{\theta}_1, \boldsymbol{\theta}_2, \rho\right) = \Phi_2\left(\Phi^{-1}\left\{F_{m_1}(y_1; \boldsymbol{\theta}_1)\right\},\ \Phi^{-1}\left\{F_{m_2}(y_2; \boldsymbol{\theta}_2)\right\};\ \rho\right).$$

If F_{m_1} and F_{m_2} are discrete, then the joint pmf follows directly from Equation (1). If F_{m_1} and F_{m_2} are continuous, then the joint pdf is obtained by elaborating Equation (2):

$$\begin{aligned} g(y_1, y_2;\ \boldsymbol{\theta}_1, \boldsymbol{\theta}_2, \rho) &= \left[\frac{\partial^2 C\left(F_{m_1}(y_1;\ \boldsymbol{\theta}_1), F_{m_2}(y_2;\ \boldsymbol{\theta}_2)\right)}{\partial F_{m_1}(y_1;\ \boldsymbol{\theta}_1) \partial F_{m_2}(y_2;\ \boldsymbol{\theta}_2)}\right] f_{m_1}(y_1;\ \boldsymbol{\theta}_1) f_{m_2}(y_2;\ \boldsymbol{\theta}_2) = \\ &= \left[\frac{\partial^2 C\left(u_1, u_2\right)}{\partial u_1 \partial u_2}\right] f_{m_1}(y_1;\ \boldsymbol{\theta}_1) f_{m_2}(y_2;\ \boldsymbol{\theta}_2) = \\ &= \left[\frac{\partial^2 \Phi_2\left(\Phi^{-1}(u_1),\ \Phi^{-1}(u_2);\ \rho\right)}{\partial u_1 \partial u_2}\right] f_{m_1}(y_1;\ \boldsymbol{\theta}_1) f_{m_2}(y_2;\ \boldsymbol{\theta}_2) = \\ &= \frac{f_{m_1}(y_1; \boldsymbol{\theta}_1) f_{m_2}(y_2; \boldsymbol{\theta}_2)}{\sqrt{1-\rho^2}} \exp\left\{\frac{-\left(\rho^2\left(q_{y_1}^2 + q_{y_2}^2\right) + 2\rho q_{y_1} q_{y_2}\right)}{2(1-\rho^2)}\right\}, \end{aligned}$$

where, for notational simplicity, we use q_{y_1} in place of $\Phi^{-1}\left\{F_{m_1}(y_1; \boldsymbol{\theta}_1)\right\}$ and q_{y_2} in place of $\Phi^{-1}\left\{F_{m_2}(y_2; \boldsymbol{\theta}_2)\right\}$.

3.2 | The Penalized Log-Likelihood

Recall from Section 2.2 of the main manuscript, that the scCOSMiX joint pmf for a single pair of negative binomial observations (y_1, y_2), conditional on fixed effects and random effects design vectors $\boldsymbol{x}, \boldsymbol{w}$, is given by:

$$f = f(y_1, y_2\,;\,\mu_1,\sigma_1,\mu_2,\sigma_2,\rho,p_1,p_2) =$$
$$= (1-p_1)(1-p_2)\,g(y_1,y_2\,;\,\mu_1,\sigma_1,\mu_2,\sigma_2,\rho)$$
$$+\begin{cases} 0 & \text{if } y_1 \neq 0 \text{ and } y_2 \neq 0 \\ (1-p_1)\,p_2 f_{\mathrm{NB}}(y_1\,;\,\mu_1,\sigma_1) & \text{if } y_1 \neq 0 \text{ and } y_2 = 0 \\ p_1(1-p_2) f_{\mathrm{NB}}(y_2\,;\,\mu_2,\sigma_2) & \text{if } y_1 = 0 \text{ and } y_2 \neq 0 \\ p_1p_2 + (1-p_1)\,p_2 f_{\mathrm{NB}}(y_1\,;\,\mu_1,\sigma_1) + p_1(1-p_2) f_{\mathrm{NB}}(y_2\,;\,\mu_2,\sigma_2) & \text{if } y_1 = 0 \text{ and } y_2 = 0 \end{cases}$$

where

$$\begin{aligned} \log\{\mu_1\} &= \boldsymbol{\delta}_{\mu_1}^\top \boldsymbol{w}_{\mu_1} + \boldsymbol{u}_{\mu_1}^\top \boldsymbol{z}_{\mu_1} + \log(S_i) \\ \log\{\mu_2\} &= \boldsymbol{\delta}_{\mu_2}^\top \boldsymbol{w}_{\mu_2} + \boldsymbol{u}_{\mu_2}^\top \boldsymbol{z}_{\mu_2} + \log(S_i) \\ \log\{\sigma_1\} &= \boldsymbol{\delta}_{\sigma_1}^\top \boldsymbol{w}_{\sigma_1} + \boldsymbol{u}_{\sigma_1}^\top \boldsymbol{z}_{\sigma_1} \\ \log\{\sigma_2\} &= \boldsymbol{\delta}_{\sigma_2}^\top \boldsymbol{w}_{\sigma_2} + \boldsymbol{u}_{\sigma_2}^\top \boldsymbol{z}_{\sigma_2} \\ \operatorname{atanh}\{\rho\} &= \boldsymbol{\delta}_{\rho}^\top \boldsymbol{w}_{\rho} + \boldsymbol{u}_{\rho}^\top \boldsymbol{z}_{\rho} \\ \operatorname{logit}\{p_1\} &= \boldsymbol{\kappa}_1^\top \boldsymbol{w}_{p_1} + \boldsymbol{u}_{p_1}^\top \boldsymbol{z}_{p_1} \\ \operatorname{logit}\{p_2\} &= \boldsymbol{\kappa}_2^\top \boldsymbol{w}_{p_2} + \boldsymbol{u}_{p_2}^\top \boldsymbol{z}_{p_2}, \end{aligned}$$

and $\boldsymbol{w}_\theta, \boldsymbol{z}_\theta$ are the (possibly improper) subset of the fixed effects and random effects vectors $\boldsymbol{w}, \boldsymbol{z}$ pertaining to parameter θ. It can be chosen as the null set if no covariate effects are desired in that parameter.

Letting $\boldsymbol{x}_\theta = (\boldsymbol{w}_\theta^\top, \boldsymbol{z}_\theta^\top)^\top$, and letting $\boldsymbol{\delta}_{\mu_1} = (\boldsymbol{\beta}_1^\top, \boldsymbol{u}_{\mu_1}^\top)^\top$, $\boldsymbol{\delta}_{\mu_2} = (\boldsymbol{\beta}_2^\top, \boldsymbol{u}_{\mu_2}^\top)^\top, \ldots, \boldsymbol{\delta}_{p_2} = (\boldsymbol{\kappa}_2^\top, \boldsymbol{u}_{p_2}^\top)^\top$ then we can write:

$$\begin{aligned} \log\{\mu_1\} &= \boldsymbol{\delta}_{\mu_1}^\top \boldsymbol{x}_{\mu_1} + \log(S_i) \\ \log\{\mu_2\} &= \boldsymbol{\delta}_{\mu_2}^\top \boldsymbol{x}_{\mu_2} + \log(S_i) \\ \log\{\sigma_1\} &= \boldsymbol{\delta}_{\sigma_1}^\top \boldsymbol{x}_{\sigma_1} \\ \log\{\sigma_2\} &= \boldsymbol{\delta}_{\sigma_2}^\top \boldsymbol{x}_{\sigma_2} \\ \operatorname{atanh}\{\rho\} &= \boldsymbol{\delta}_{\rho}^\top \boldsymbol{x}_{\rho} \\ \operatorname{logit}\{p_1\} &= \boldsymbol{\delta}_{p_1}^\top \boldsymbol{x}_{p_1} \\ \operatorname{logit}\{p_2\} &= \boldsymbol{\delta}_{p_2}^\top \boldsymbol{x}_{p_2}. \end{aligned}$$

Then we can express the penalized log-likelihood for one pair of observations (y_1, y_2) as:

$$l = l(\boldsymbol{\delta}; y_1, y_2) = \log(f(y_1, y_2;\ \boldsymbol{\delta})) - \frac{1}{2}\sum_{\theta\in\Theta} \boldsymbol{u}_\theta^\top \operatorname{diag}\{\boldsymbol{\lambda}_\theta\}\,\boldsymbol{u}_\theta$$

3.3 | Gradient

3.3.1 | Derivative of penalized log-likelihood with respect to $\boldsymbol{\delta}_{p_1}$

$$\frac{\partial l}{\partial \boldsymbol{\delta}_{p_1}} = \frac{1}{f}\frac{\partial f}{\partial p_1}\frac{\partial p_1}{\partial \boldsymbol{\delta}_{p_1}} - \operatorname{diag}\left\{\boldsymbol{\lambda}_{p_1}\right\}\boldsymbol{u}_{p_1}$$

where

$$\begin{aligned} \frac{\partial}{\partial \boldsymbol{\delta}_{p_1}} p_1 &= \frac{\partial}{\partial \boldsymbol{\delta}_{p_1}} \operatorname{sigmoid}\left\{\boldsymbol{\delta}_{p_1}^\top \boldsymbol{x}_{p_1}\right\} \\ &= \operatorname{sigmoid}\left\{\boldsymbol{\delta}_{p_1}^\top \boldsymbol{x}_{p_1}\right\}\left(1 - \operatorname{sigmoid}\left\{\boldsymbol{\delta}_{p_1}^\top \boldsymbol{x}_{p_1}\right\}\right)\boldsymbol{x}_{p_1} \end{aligned}$$

and

$$\frac{\partial}{\partial p_1} f =$$
$$= \begin{cases} -(1-p_2)g(y_1,y_2|\mu_1,\sigma_1,\mu_2,\sigma_2,\rho) & \text{if } y_1 \neq 0 \text{ and } y_2 \neq 0 \\ -(1-p_2)g(y_1,y_2|\mu_1,\sigma_1,\mu_2,\sigma_2,\rho) - p_2 f_{\mathrm{NB}}(y_1;\mu_1,\sigma_1) & \text{if } y_1 \neq 0 \text{ and } y_2 = 0 \\ -(1-p_2)g(y_1,y_2|\mu_1,\sigma_1,\mu_2,\sigma_2,\rho) + (1-p_2) f_{\mathrm{NB}}(y_2;\mu_2,\sigma_2) & \text{if } y_1 = 0 \text{ and } y_2 \neq 0 \\ -(1-p_2)g(y_1,y_2|\mu_1,\sigma_1,\mu_2,\sigma_2,\rho) + p_2 - p_2 f_{\mathrm{NB}}(y_1\,;\,\mu_1,\sigma_1) + (1-p_2) f_{\mathrm{NB}}(y_2\,;\,\mu_2,\sigma_2) & \text{if } y_1 = 0 \text{ and } y_2 = 0 \end{cases}$$

3.3.2 | Derivative of penalized log-likelihood with respect to δ_{p_2}

$$\frac{\partial l}{\partial \boldsymbol{\delta}_{p_2}} = \frac{1}{f}\frac{\partial f}{\partial p_2}\frac{\partial p_2}{\partial \boldsymbol{\delta}_{p_2}} - \mathrm{diag}\left\{\boldsymbol{\lambda}_{p_2}\right\}\boldsymbol{u}_{p_2}$$

where

$$\begin{aligned} \frac{\partial}{\partial \boldsymbol{\delta}_{p_2}} p_2 &= \frac{\partial}{\partial \boldsymbol{\delta}_{p_2}} \mathrm{sigmoid}\left\{\boldsymbol{\delta}_{p_2}^{\top}\boldsymbol{x}_{p_2}\right\} \\ &= \mathrm{sigmoid}\left\{\boldsymbol{\delta}_{p_2}^{\top}\boldsymbol{x}_{p_2}\right\}\left(1 - \mathrm{sigmoid}\left\{\boldsymbol{\delta}_{p_2}^{\top}\boldsymbol{x}_{p_2}\right\}\right)\boldsymbol{x}_{p_2} \end{aligned}$$

and

$$\frac{\partial}{\partial p_1} f =$$
$$= \begin{cases} -(1-p_1)g(y_1,y_2|\mu_1,\sigma_1,\mu_2,\sigma_2,\rho) & \text{if } y_1 \neq 0 \text{ and } y_2 \neq 0 \\ -(1-p_1)g(y_1,y_2|\mu_1,\sigma_1,\mu_2,\sigma_2,\rho) + (1-p_1) f_{\mathrm{NB}}(y_1;\mu_1,\sigma_1) & \text{if } y_1 \neq 0 \text{ and } y_2 = 0 \\ -(1-p_1)g(y_1,y_2|\mu_1,\sigma_1,\mu_2,\sigma_2,\rho) - p_1 f_{\mathrm{NB}}(y_2;\mu_2,\sigma_2) & \text{if } y_1 = 0 \text{ and } y_2 \neq 0 \\ -(1-p_1)g(y_1,y_2|\mu_1,\sigma_1,\mu_2,\sigma_2,\rho) + p_1 + (1-p_1) f_{\mathrm{NB}}(y_1\,;\,\mu_1,\sigma_1) - p_1 f_{\mathrm{NB}}(y_2\,;\,\mu_2,\sigma_2) & \text{if } y_1 = 0 \text{ and } y_2 = 0 \end{cases}$$

3.3.3 | Derivative of penalized log-likelihood with respect to δ_{μ_1}

$$\frac{\partial l}{\partial \boldsymbol{\delta}_{\mu_1}} = \frac{1}{f}\frac{\partial f}{\partial \mu_1}\frac{\partial \mu_1}{\partial \boldsymbol{\delta}_{\mu_1}} - \mathrm{diag}\left\{\boldsymbol{\lambda}_{\mu_1}\right\}\boldsymbol{u}_{\mu_1}$$

where

$$\begin{aligned} \frac{\partial}{\partial \boldsymbol{\delta}_{\mu_1}} \mu_1 &= \frac{\partial}{\partial \boldsymbol{\delta}_{\mu_1}} \exp\left\{\boldsymbol{\delta}_{\mu_1}^{\top}\boldsymbol{x}_{\mu_1}\right\} \\ &= \exp\left\{\boldsymbol{\delta}_{\mu_1}^{\top}\boldsymbol{x}_{\mu_1}\right\}\boldsymbol{x}_{\mu_1} \end{aligned}$$

and

$$\frac{\partial f}{\partial \mu_1} =$$
$$= \begin{cases} (1-p_1)(1-p_2)\frac{\partial}{\partial \mu_1} g(y_1,y_2|\mu_1,\sigma_1,\mu_2,\sigma_2,\rho) & \text{if } y_1 \neq 0, y_2 \neq 0 \\ (1-p_1)(1-p_2)\frac{\partial}{\partial \mu_1} g(y_1,y_2|\mu_1,\sigma_1,\mu_2,\sigma_2,\rho) + (1-p_1)p_2 \frac{\partial}{\partial \mu_1} f_{\mathrm{NB}}(y_1;\mu_1,\sigma_1) & \text{if } y_1 \neq 0, y_2 = 0 \\ (1-p_1)(1-p_2)\frac{\partial}{\partial \mu_1} g(y_1,y_2|\mu_1,\sigma_1,\mu_2,\sigma_2,\rho) & \text{if } y_1 = 0, y_2 \neq 0 \\ (1-p_1)(1-p_2)\frac{\partial}{\partial \mu_1} g(y_1,y_2|\mu_1,\sigma_1,\mu_2,\sigma_2,\rho) + (1-p_1)\,p_2\,\frac{\partial}{\partial \mu_1} f_{\mathrm{NB}}(y_1\,;\,\mu_1,\sigma_1) & \text{if } y_1 = 0, y_2 = 0 \end{cases}$$

where

$$
\begin{aligned}
&\frac{\partial}{\partial \mu_1} g(y_1, y_2 | \mu_1, \sigma_1, \mu_2, \sigma_2, \rho) = \\
=&\frac{\partial}{\partial \mu_1} \int_{\Phi^{-1}\{F_{\mathrm{NB}}(y_1-1;\mu_1,\sigma_1)\}}^{\Phi^{-1}\{F_{\mathrm{NB}}(y_1;\mu_1,\sigma_1)\}} \int_{\Phi^{-1}\{F_{\mathrm{NB}}(y_2-1;\mu_2,\sigma_2)\}}^{\Phi^{-1}\{F_{\mathrm{NB}}(y_2;\mu_2,\sigma_2)\}} \phi_2(t_1, t_2|\rho) dt_2 dt_1 \\
=&\frac{\partial g(y_1, y_2|\mu_1, \sigma_1, \mu_2, \sigma_2, \rho)}{F_{\mathrm{NB}}(y_1; \mu_1, \sigma_1)} \frac{\partial F_{\mathrm{NB}}(y_1; \mu_1, \sigma_1)}{\partial \mu_1} + \\
+&\frac{\partial g(y_1, y_2|\mu_1, \sigma_1, \mu_2, \sigma_2, \rho)}{\partial F_{\mathrm{NB}}(y_1-1; \mu_1, \sigma_1)} \frac{\partial F_{\mathrm{NB}}(y_1-1; \mu_1, \sigma_1)}{\partial \mu_1}
\end{aligned}
$$

and

$$
\begin{aligned}
&\frac{\partial g(y_1, y_2|\mu_1, \sigma_1, \mu_2, \sigma_2, \rho)}{\partial F_{\mathrm{NB}}(y_1; \mu_1, \sigma_1)} = \\
=&\Phi\left(\frac{\Phi^{-1}\{F_{\mathrm{NB}}(y_2; \mu_2, \sigma_2)\} - \Phi^{-1}\{F_{\mathrm{NB}}(y_1; \mu_1, \sigma_1)\}\rho}{\sqrt{1-\rho^2}}\right) + \\
-&\Phi\left(\frac{\Phi^{-1}\{F_{\mathrm{NB}}(y_2-1; \mu_2, \sigma_2)\} - \Phi^{-1}\{F_{\mathrm{NB}}(y_1; \mu_1, \sigma_1)\}\rho}{\sqrt{1-\rho^2}}\right)
\end{aligned}
$$

while

$$
\begin{aligned}
&\frac{\partial g(y_1, y_2|\mu_1, \sigma_1, \mu_2, \sigma_2, \rho)}{\partial F_{\mathrm{NB}}(y_1-1; \mu_1, \sigma_1)} = \\
=&-\Phi\left(\frac{\Phi^{-1}\{F_{\mathrm{NB}}(y_2; \mu_2, \sigma_2)\} - \Phi^{-1}\{F_{\mathrm{NB}}(y_1-1; \mu_1, \sigma_1)\}\rho}{\sqrt{1-\rho^2}}\right) + \\
+&\Phi\left(\frac{\Phi^{-1}\{F_{\mathrm{NB}}(y_2-1; \mu_2, \sigma_2)\} - \Phi^{-1}\{F_{\mathrm{NB}}(y_1-1; \mu_1, \sigma_1)\}\rho}{\sqrt{1-\rho^2}}\right)
\end{aligned}
$$

and

$$
\begin{aligned}
&\frac{\partial F_{\mathrm{NB}}(y_1; \mu_1, \sigma_1)}{\partial \mu_1} = \sum_{k=0}^{y_1} \frac{\partial f_{\mathrm{NB}}(k; \mu_1, \sigma_1)}{\partial \mu_1} = \\
=&\sum_{k=0}^{y_1} \frac{\Gamma(k+\frac{1}{\sigma_1})}{\Gamma(1+k)\Gamma(\frac{1}{\sigma_1})} \left(\frac{\frac{1}{\sigma_1}}{\frac{1}{\sigma_1}+\mu_1}\right)^{\frac{1}{\sigma_1}} \left(\frac{\mu_1}{\frac{1}{\sigma_1}+\mu_1}\right)^{k} \times \left[\frac{k}{\mu_1} - \frac{\frac{1}{\sigma_1}+k}{\frac{1}{\sigma_1}+\mu_1}\right]
\end{aligned}
$$

3.3.4 Derivative of penalized log-likelihood with respect to δ_{μ_2}

$$
\frac{\partial l}{\partial \boldsymbol{\delta}_{\mu_2}} = \frac{1}{f} \frac{\partial f}{\partial \mu_2} \frac{\partial \mu_2}{\partial \boldsymbol{\delta}_{\mu_2}} - \operatorname{diag}\left\{\boldsymbol{\lambda}_{\mu_2}\right\} \boldsymbol{u}_{\mu_2}
$$

where

$$
\begin{aligned}
\frac{\partial}{\partial \boldsymbol{\delta}_{\mu_2}} \mu_2 &= \frac{\partial}{\partial \boldsymbol{\delta}_{\mu_2}} \exp\left\{\boldsymbol{\delta}_{\mu_2}^{\top} \boldsymbol{x}_{\mu_2}\right\} \\
&= \exp\left\{\boldsymbol{\delta}_{\mu_2}^{\top} \boldsymbol{x}_{\mu_2}\right\} \boldsymbol{x}_{\mu_2}
\end{aligned}
$$

and

$$
\frac{\partial f}{\partial \mu_2} =
$$
$$
= \begin{cases}
(1-p_1)(1-p_2)\frac{\partial}{\partial \mu_2} g(y_1, y_2|\mu_1, \sigma_1, \mu_2, \sigma_2, \rho) & \text{if } y_1 \neq 0, y_2 \neq 0 \\
(1-p_1)(1-p_2)\frac{\partial}{\partial \mu_2} g(y_1, y_2|\mu_1, \sigma_1, \mu_2, \sigma_2, \rho) & \text{if } y_1 \neq 0, y_2 = 0 \\
(1-p_1)(1-p_2)\frac{\partial}{\partial \mu_2} g(y_1, y_2|\mu_1, \sigma_1, \mu_2, \sigma_2, \rho) + p_1(1-p_2)\frac{\partial}{\partial \mu_2} f_{\mathrm{NB}}(y_2; \mu_2, \sigma_2) & \text{if } y_1 = 0, y_2 \neq 0 \\
(1-p_1)(1-p_2)\frac{\partial}{\partial \mu_2} g(y_1, y_2|\mu_1, \sigma_1, \mu_2, \sigma_2, \rho) + p_1(1-p_2)\frac{\partial}{\partial \mu_2} f_{\mathrm{NB}}(y_2; \mu_2, \sigma_2) & \text{if } y_1 = 0, y_2 = 0
\end{cases}
$$

where

$$
\begin{aligned}
&\frac{\partial}{\partial \mu_2} g(y_1, y_2 | \mu_1, \sigma_1, \mu_2, \sigma_2, \rho) = \\
=&\frac{\partial}{\partial \mu_2} \int_{\Phi^{-1}\{F_{\mathrm{NB}}(y_1-1;\mu_1,\sigma_1)\}}^{\Phi^{-1}\{F_{\mathrm{NB}}(y_1;\mu_1,\sigma_1)\}} \int_{\Phi^{-1}\{F_{\mathrm{NB}}(y_2-1;\mu_2,\sigma_2)\}}^{\Phi^{-1}\{F_{\mathrm{NB}}(y_2;\mu_2,\sigma_2)\}} \phi_2(t_1, t_2 | \rho) dt_2 dt_1 \\
=&\frac{\partial g(y_1, y_2 | \mu_1, \sigma_1, \mu_2, \sigma_2, \rho)}{F_{\mathrm{NB}}(y_2; \mu_2, \sigma_2)} \frac{\partial F_{\mathrm{NB}}(y_2; \mu_2, \sigma_2)}{\partial \mu_2} + \\
+&\frac{\partial g(y_1, y_2 | \mu_1, \sigma_1, \mu_2, \sigma_2, \rho)}{\partial F_{\mathrm{NB}}(y_2 - 1; \mu_2, \sigma_2)} \frac{\partial F_{\mathrm{NB}}(y_2 - 1; \mu_2, \sigma_2)}{\partial \mu_2}
\end{aligned}
$$

and

$$
\begin{aligned}
&\frac{\partial g(y_1, y_2 | \mu_1, \sigma_1, \mu_2, \sigma_2, \rho)}{\partial F_{\mathrm{NB}}(y_2; \mu_2, \sigma_2)} = \\
=&\Phi\left(\frac{\Phi^{-1}\{F_{\mathrm{NB}}(y_1; \mu_1, \sigma_1)\} - \Phi^{-1}\{F_{\mathrm{NB}}(y_2; \mu_2, \sigma_2)\}\rho}{\sqrt{1-\rho^2}} \right) + \\
-&\Phi\left(\frac{\Phi^{-1}\{F_{\mathrm{NB}}(y_1 - 1; \mu_1, \sigma_1)\} - \Phi^{-1}\{F_{\mathrm{NB}}(y_2; \mu_2, \sigma_2)\}\rho}{\sqrt{1-\rho^2}} \right)
\end{aligned}
$$

while

$$
\begin{aligned}
&\frac{\partial g(y_1, y_2 | \mu_1, \sigma_1, \mu_2, \sigma_2, \rho)}{\partial F_{\mathrm{NB}}(y_2 - 1; \mu_2, \sigma_2)} = \\
=&-\Phi\left(\frac{\Phi^{-1}\{F_{\mathrm{NB}}(y_1; \mu_1, \sigma_1)\} - \Phi^{-1}\{F_{\mathrm{NB}}(y_2 - 1; \mu_2, \sigma_2)\}\rho}{\sqrt{1-\rho^2}} \right) + \\
+&\Phi\left(\frac{\Phi^{-1}\{F_{\mathrm{NB}}(y_1 - 1; \mu_1, \sigma_1)\} - \Phi^{-1}\{F_{\mathrm{NB}}(y_2 - 1; \mu_2, \sigma_2)\}\rho}{\sqrt{1-\rho^2}} \right)
\end{aligned}
$$

and

$$
\begin{aligned}
\frac{\partial F_{\mathrm{NB}}(y_2; \mu_1, \sigma_1)}{\partial \mu_2} &= \sum_{k=0}^{y_2} \frac{\partial f_{\mathrm{NB}}(k; \mu_1, \sigma_1)}{\partial \mu_2} = \\
&= \sum_{k=0}^{y_2} \frac{\Gamma(k + \frac{1}{\sigma_2})}{\Gamma(1+k)\Gamma(\frac{1}{\sigma_2})} \left(\frac{\frac{1}{\sigma_2}}{\frac{1}{\sigma_2} + \mu_1} \right)^{\frac{1}{\sigma_2}} \left(\frac{\mu_2}{\frac{1}{\sigma_2} + \mu_2} \right)^k \times \left[\frac{k}{\mu_2} - \frac{\frac{1}{\sigma_2} + k}{\frac{1}{\sigma_2} + \mu_2} \right]
\end{aligned}
$$

3.3.5 | Derivative of penalized log-likelihood with respect to δ_{σ_1}

$$
\frac{\partial l}{\partial \boldsymbol{\delta}_{\sigma_1}} = \frac{1}{f} \frac{\partial f}{\partial \sigma_1} \frac{\partial \sigma_1}{\partial \boldsymbol{\delta}_{\sigma_1}} - \operatorname{diag}\left\{ \boldsymbol{\lambda}_{\sigma_1} \right\} \boldsymbol{u}_{\sigma_1}
$$

where

$$
\begin{aligned}
\frac{\partial \sigma_1}{\partial \boldsymbol{\delta}_{\sigma_1}} &= \frac{\partial}{\partial \boldsymbol{\delta}_{\sigma_1}} \exp\left\{ \boldsymbol{\delta}_{\sigma_1}^\top \boldsymbol{x}_{\sigma_1} \right\} \\
&= \exp\left\{ \boldsymbol{\delta}_{\sigma_1}^\top \boldsymbol{x}_{\sigma_1} \right\} \boldsymbol{x}_{\sigma_1}
\end{aligned}
$$

and

$$
\begin{aligned}
&\frac{\partial f}{\partial \sigma_1} = \\
&= \begin{cases}
(1-p_1)(1-p_2)\frac{\partial}{\partial \sigma_1} g(y_1, y_2 | \mu_1, \sigma_1, \mu_2, \sigma_2, \rho) & \text{if } y_1 \neq 0, y_2 \neq 0 \\
(1-p_1)(1-p_2)\frac{\partial}{\partial \sigma_1} g(y_1, y_2 | \mu_1, \sigma_1, \mu_2, \sigma_2, \rho) + (1-p_1)p_2 \frac{\partial}{\partial \sigma_1} f_{\mathrm{NB}}(y_1; \mu_1, \sigma_1) & \text{if } y_1 \neq 0, y_2 = 0 \\
(1-p_1)(1-p_2)\frac{\partial}{\partial \sigma_1} g(y_1, y_2 | \mu_1, \sigma_1, \mu_2, \sigma_2, \rho) & \text{if } y_1 = 0, y_2 \neq 0 \\
(1-p_1)(1-p_2)\frac{\partial}{\partial \sigma_1} g(y_1, y_2 | \mu_1, \sigma_1, \mu_2, \sigma_2, \rho) + (1-p_1)\, p_2 \, \frac{\partial}{\partial \sigma_1} f_{\mathrm{NB}}(y_1\,;\, \mu_1, \sigma_1) & \text{if } y_1 = 0, y_2 = 0
\end{cases}
\end{aligned}
$$

where

$$\begin{aligned}
&\frac{\partial}{\partial\sigma_1} g(y_1, y_2|\mu_1, \sigma_1, \mu_2, \sigma_2, \rho) = \\
=&\frac{\partial}{\partial\sigma_1}\int_{\Phi^{-1}\{F_{\mathrm{NB}}(y_1-1;\mu_1,\sigma_1)\}}^{\Phi^{-1}\{F_{\mathrm{NB}}(y_1;\mu_1,\sigma_1)\}}\int_{\Phi^{-1}\{F_{\mathrm{NB}}(y_2-1;\mu_2,\sigma_2)\}}^{\Phi^{-1}\{F_{\mathrm{NB}}(y_2;\mu_2,\sigma_2)\}} \phi_2(t_1, t_2|\rho) dt_2 dt_1 \\
=&\frac{\partial g(y_1, y_2|\mu_1, \sigma_1, \mu_2, \sigma_2, \rho)}{F_{\mathrm{NB}}(y_1; \mu_1, \sigma_1)} \frac{\partial F_{\mathrm{NB}}(y_1; \mu_1, \sigma_1)}{\partial\sigma_1} + \\
+&\frac{\partial g(y_1, y_2|\mu_1, \sigma_1, \mu_2, \sigma_2, \rho)}{\partial F_{\mathrm{NB}}(y_1-1; \mu_1, \sigma_1)} \frac{\partial F_{\mathrm{NB}}(y_1-1; \mu_1, \sigma_1)}{\partial\sigma_1}
\end{aligned}$$

We have given $\frac{\partial g(y_1,y_2|\mu_1,\sigma_1,\mu_2,\sigma_2,\rho)}{F_{\mathrm{NB}}(y_1;\mu_1,\sigma_1)}$ and $\frac{\partial c(y_1-1,y_2|\mu_1,\sigma_1,\mu_2,\sigma_2,\rho)}{F_{\mathrm{NB}}(y_1-1;\mu_1,\sigma_1)}$ in subsection 3.3.3, the remaining terms are:

$$\begin{aligned}
&\frac{\partial F_{\mathrm{NB}}(y_1; \mu_1, \sigma_1)}{\partial\sigma_1} = \sum_{k=0}^{y_1} \frac{\partial f_{\mathrm{NB}}(k; \mu_1, \sigma_1)}{\partial\sigma_1} = \\
=&\sum_{k=0}^{y_1} \frac{-1}{\sigma_1^2} \frac{\Gamma(k+\frac{1}{\sigma_1})}{\Gamma(1+k)\Gamma(\frac{1}{\sigma_1})(\frac{1}{\sigma_1}+\mu_1)} \left(\frac{\frac{1}{\sigma_1}}{\frac{1}{\sigma_1}+\mu_1}\right)^{\frac{1}{\sigma_1}} \left(\frac{\mu_1}{\frac{1}{\sigma_1}+\mu_1}\right)^{k} \\
&\times\left(-k+\mu_1+\left[\frac{1}{\sigma_1}+\mu_1\right]\left[\log\left(\frac{\frac{1}{\sigma_1}}{\frac{1}{\sigma_1}+\mu_1}\right)+\psi(k+\frac{1}{\sigma_1})-\psi(\frac{1}{\sigma_1})\right]\right)
\end{aligned}$$

3.3.6 | Derivative of penalized log-likelihood with respect to $\boldsymbol{\delta}_{\sigma_2}$

$$\frac{\partial l}{\partial\boldsymbol{\delta}_{\sigma_2}} = \frac{1}{f}\frac{\partial f}{\partial\sigma_2}\frac{\partial\sigma_2}{\partial\boldsymbol{\delta}_{\sigma_2}} - \mathrm{diag}\left\{\boldsymbol{\lambda}_{\sigma_2}\right\}\boldsymbol{u}_{\sigma_2}$$

where

$$\begin{aligned}
\frac{\partial\sigma_2}{\partial\boldsymbol{\delta}_{\sigma_2}} &= \frac{\partial}{\partial\boldsymbol{\delta}_{\sigma_2}}\exp\left\{\boldsymbol{\delta}_{\sigma_2}^{\top}\boldsymbol{x}_{\sigma_2}\right\} \\
&= \exp\left\{\boldsymbol{\delta}_{\sigma_2}^{\top}\boldsymbol{x}_{\sigma_2}\right\}\boldsymbol{x}_{\sigma_2}
\end{aligned}$$

and

$$\begin{aligned}
&\frac{\partial f}{\partial\sigma_2} = \\
=&\begin{cases}
(1-p_1)(1-p_2)\frac{\partial}{\partial\sigma_2}g(y_1, y_2|\mu_1, \sigma_1, \mu_2, \sigma_2, \rho) & \text{if } y_1 \neq 0, y_2 \neq 0 \\
(1-p_1)(1-p_2)\frac{\partial}{\partial\sigma_2}g(y_1, y_2|\mu_1, \sigma_1, \mu_2, \sigma_2, \rho) & \text{if } y_1 \neq 0, y_2 = 0 \\
(1-p_1)(1-p_2)\frac{\partial}{\partial\sigma_2}g(y_1, y_2|\mu_1, \sigma_1, \mu_2, \sigma_2, \rho) + p_1(1-p_2)\frac{\partial}{\partial\sigma_2}f_{\mathrm{NB}}(y_2; \mu_2, \sigma_2) & \text{if } y_1 = 0, y_2 \neq 0 \\
(1-p_1)(1-p_2)\frac{\partial}{\partial\sigma_2}g(y_1, y_2|\mu_1, \sigma_1, \mu_2, \sigma_2, \rho) + p_1(1-p_2)\frac{\partial}{\partial\sigma_2}f_{\mathrm{NB}}(y_2; \mu_2, \sigma_2) & \text{if } y_1 = 0, y_2 = 0
\end{cases}
\end{aligned}$$

where

$$\begin{aligned}
&\frac{\partial}{\partial\sigma_2} g(y_1, y_2|\mu_1, \sigma_1, \mu_2, \sigma_2, \rho) = \\
=&\frac{\partial}{\partial\sigma_2}\int_{\Phi^{-1}\{F_{\mathrm{NB}}(y_1-1;\mu_1,\sigma_1)\}}^{\Phi^{-1}\{F_{\mathrm{NB}}(y_1;\mu_1,\sigma_1)\}}\int_{\Phi^{-1}\{F_{\mathrm{NB}}(y_2-1;\mu_2,\sigma_2)\}}^{\Phi^{-1}\{F_{\mathrm{NB}}(y_2;\mu_2,\sigma_2)\}} \phi_2(t_1, t_2|\rho) dt_2 dt_1 \\
=&\frac{\partial g(y_1, y_2|\mu_1, \sigma_1, \mu_2, \sigma_2, \rho)}{F_{\mathrm{NB}}(y_2; \mu_2, \sigma_2)} \frac{\partial F_{\mathrm{NB}}(y_2; \mu_2, \sigma_2)}{\partial\sigma_2} + \\
+&\frac{\partial g(y_1, y_2|\mu_1, \sigma_1, \mu_2, \sigma_2, \rho)}{\partial F_{\mathrm{NB}}(y_2-1; \mu_2, \sigma_2)} \frac{\partial F_{\mathrm{NB}}(y_2-1; \mu_2, \sigma_2)}{\partial\sigma_2}
\end{aligned}$$

We have given $\frac{\partial g(y_1,y_2|\mu_1,\sigma_1,\mu_2,\sigma_2,\rho)}{F_{\mathrm{NB}}(y_2;\mu_2,\sigma_2)}$ and $\frac{\partial c(y_1-1,y_2|\mu_1,\sigma_1,\mu_2,\sigma_2,\rho)}{F_{\mathrm{NB}}(y_2-1;\mu_2,\sigma_2)}$ in subsection 3.3.4, the remaining terms are:

$$\frac{\partial F_{\mathrm{NB}}(y_2;\mu_2,\sigma_2)}{\partial \sigma_2} = \sum_{k=0}^{y_2} \frac{\partial f_{\mathrm{NB}}(k;\mu_2,\sigma_2)}{\partial \sigma_2} =$$

$$= \sum_{k=0}^{y_2} \frac{-1}{\sigma_2^2} \frac{\Gamma(k+\frac{1}{\sigma_2})}{\Gamma(1+k)\Gamma(\frac{1}{\sigma_2})(\frac{1}{\sigma_2}+\mu_2)} \left(\frac{\frac{1}{\sigma_2}}{\frac{1}{\sigma_2}+\mu_2}\right)^{\frac{1}{\sigma_2}} \left(\frac{\mu_2}{\frac{1}{\sigma_2}+\mu_2}\right)^{k}$$

$$\times \left(-k+\mu_2+\left[\frac{1}{\sigma_2}+\mu_2\right]\left[\log\left(\frac{\frac{1}{\sigma_2}}{\frac{1}{\sigma_2}+\mu_2}\right)+\psi(k+\frac{1}{\sigma_2})-\psi(\frac{1}{\sigma_2})\right]\right)$$

3.3.7 | Derivative of penalized log-likelihood with respect to δ_ρ

$$\frac{\partial l}{\partial \boldsymbol{\delta}_\rho} = \frac{1}{f}\frac{\partial f}{\partial \rho}\frac{\partial \rho}{\partial \boldsymbol{\delta}_\rho} - \mathrm{diag}\left\{\boldsymbol{\lambda}_\rho\right\}\boldsymbol{u}_\rho$$

where

$$\begin{aligned}\frac{\partial \rho}{\partial \boldsymbol{\delta}_\rho} &= \frac{\partial}{\partial \boldsymbol{\delta}_\rho}\rho \\ &= \frac{\partial}{\partial \boldsymbol{\delta}_\rho}\tanh\left\{\boldsymbol{\delta}_\rho^\top \boldsymbol{x}_\rho\right\} \\ &= \cosh^2\left\{\boldsymbol{\delta}_\rho^\top \boldsymbol{x}_\rho\right\}\boldsymbol{x}_\rho\end{aligned}$$

and

$$\frac{\partial f}{\partial \rho} =$$

$$= \begin{cases} (1-p_1)(1-p_2)\frac{\partial}{\partial\rho}g(y_1,y_2|\mu_1,\sigma_1,\mu_2,\sigma_2,\rho) & \text{if } y_1 \neq 0 \text{ and } y_2 \neq 0 \\ (1-p_1)(1-p_2)\frac{\partial}{\partial\rho}g(y_1,y_2|\mu_1,\sigma_1,\mu_2,\sigma_2,\rho) & \text{if } y_1 \neq 0 \text{ and } y_2 = 0 \\ (1-p_1)(1-p_2)\frac{\partial}{\partial\rho}g(y_1,y_2|\mu_1,\sigma_1,\mu_2,\sigma_2,\rho) & \text{if } y_1 = 0 \text{ and } y_2 \neq 0 \\ (1-p_1)(1-p_2)\frac{\partial}{\partial\rho}g(y_1,y_2|\mu_1,\sigma_1,\mu_2,\sigma_2,\rho) & \text{if } y_1 = 0 \text{ and } y_2 = 0 \end{cases} =$$

$$=(1-p_1)(1-p_2)\frac{\partial}{\partial\rho}g(y_1,y_2|\mu_1,\sigma_1,\mu_2,\sigma_2,\rho)$$

where

$$\frac{\partial}{\partial\rho}g(y_1,y_2|\mu_1,\sigma_1,\mu_2,\sigma_2,\rho) =$$

$$= \int_{\Phi^{-1}\{F_{\mathrm{NB}}(y_1-1;\mu_1,\sigma_1)\}}^{\Phi^{-1}\{F_{\mathrm{NB}}(y_1;\mu_1,\sigma_1)\}} \int_{\Phi^{-1}\{F_{\mathrm{NB}}(y_2-1;\mu_2,\sigma_2)\}}^{\Phi^{-1}\{F_{\mathrm{NB}}(y_2;\mu_2,\sigma_2)\}} \left[\frac{\rho}{1-\rho^2} + \frac{(\rho t_1 - t_2)(\rho t_2 - t_1)}{(1-\rho^2)^2}\right] \phi_2(t_1,t_2|\rho)dt_2dt_1$$

3.4 | Hessian

3.4.1 | 2nd derivative with respect to δ_{p_1}

$$\frac{\partial^2 l}{\partial \boldsymbol{\delta}_{p_1}^2} =$$

$$= \frac{\partial}{\partial \boldsymbol{\delta}_{p_1}}\frac{\partial l}{\partial \boldsymbol{\delta}_{p_1}}$$

$$= \frac{\partial}{\partial \boldsymbol{\delta}_{p_1}}\left(\frac{1}{f}\frac{\partial f}{\partial p_1}\frac{\partial p_1}{\partial \boldsymbol{\delta}_{p_1}} - \mathrm{diag}\left\{\boldsymbol{\lambda}_{p_1}\right\}\boldsymbol{u}_{p_1}\right)$$

$$= \left(\frac{\partial}{\partial \boldsymbol{\delta}_{p_1}}\frac{1}{f}\right)\frac{\partial f}{\partial p_1}\frac{\partial p_1}{\partial \boldsymbol{\delta}_{p_1}} + \frac{1}{f}\left(\frac{\partial}{\partial \boldsymbol{\delta}_{p_1}}\frac{\partial f}{\partial p_1}\right)\frac{\partial p_1}{\partial \boldsymbol{\delta}_{p_1}} + \frac{1}{f}\frac{\partial f}{\partial p_1}\left(\frac{\partial}{\partial \boldsymbol{\delta}_{p_1}}\frac{\partial p_1}{\partial \boldsymbol{\delta}_{p_1}}\right) - \mathrm{diag}\left\{\boldsymbol{\lambda}_{p_1}\right\}$$

where

$$\frac{\partial}{\partial \boldsymbol{\delta}_{p_1}} \frac{1}{f} = \frac{-1}{f^2} \frac{\partial f}{\partial \boldsymbol{\delta}_{p_1}}$$

where $\frac{\partial f}{\partial \boldsymbol{\delta}_{p_1}}$ was already calculated in subsection 3.3.5, and

$$\begin{aligned}
&\frac{\partial}{\partial \boldsymbol{\delta}_{p_1}} \frac{\partial f}{\partial p_1} = \\
=&\frac{\partial p_1}{\partial \boldsymbol{\delta}_{p_1}} \left(\frac{\partial}{\partial \boldsymbol{\delta}_{p_1}} \frac{\partial f}{\partial p_1} \right) \\
=&\frac{\partial p_1}{\partial \boldsymbol{\delta}_{p_1}} \frac{\partial}{\partial p_1} \begin{cases} -(1-p_2) g(y_1, y_2 | \mu_1, \sigma_1, \mu_2, \sigma_2, \rho) & \text{if } y_1 \neq 0 \text{ and } y_2 \neq 0 \\ -(1-p_2) g(y_1, y_2 | \mu_1, \sigma_1, \mu_2, \sigma_2, \rho) - p_2 f_{\mathrm{NB}}(y_1; \mu_1, \sigma_1) & \text{if } y_1 \neq 0 \text{ and } y_2 = 0 \\ -(1-p_2) g(y_1, y_2 | \mu_1, \sigma_1, \mu_2, \sigma_2, \rho) + (1-p_2) f_{\mathrm{NB}}(y_2; \mu_2, \sigma_2) & \text{if } y_1 = 0 \text{ and } y_2 \neq 0 \\ -(1-p_2) g(y_1, y_2 | \mu_1, \sigma_1, \mu_2, \sigma_2, \rho) + p_2 - p_2 f_{\mathrm{NB}}(y_1 ; \mu_1, \sigma_1) + (1-p_2) f_{\mathrm{NB}}(y_2 ; \mu_2, \sigma_2) & \text{if } y_1 = 0 \text{ and } y_2 = 0 \end{cases} \\
=&0
\end{aligned}$$

and

$$\begin{aligned}
\frac{\partial}{\partial \boldsymbol{\delta}_{p_1}} \frac{\partial p_1}{\partial \boldsymbol{\delta}_{p_1}} &= \frac{\partial}{\partial \boldsymbol{\delta}_{p_1}} \left(\mathrm{sigmoid}\left\{ \boldsymbol{\delta}_{p_1}^\top \boldsymbol{x}_{p_1} \right\} \left(1 - \mathrm{sigmoid}\left\{ \boldsymbol{\delta}_{p_1}^\top \boldsymbol{x}_{p_1} \right\} \right) \boldsymbol{x}_{p_1} \right) \\
&= \left(\frac{\partial}{\partial \boldsymbol{\delta}_{p_1}} \mathrm{sigmoid}\left\{ \boldsymbol{\delta}_{p_1}^\top \boldsymbol{x}_{p_1} \right\} \right) \left(1 - \mathrm{sigmoid}\left\{ \boldsymbol{\delta}_{p_1}^\top \boldsymbol{x}_{p_1} \right\} \right) \boldsymbol{x}_{p_1} \boldsymbol{x}_{p_1}^\top + \\
&+ \mathrm{sigmoid}\left\{ \boldsymbol{\delta}_{p_1}^\top \boldsymbol{x}_{p_1} \right\} \left(1 - \frac{\partial}{\partial \boldsymbol{\delta}_{p_1}} \mathrm{sigmoid}\left\{ \boldsymbol{\delta}_{p_1}^\top \boldsymbol{x}_{p_1} \right\} \right) \boldsymbol{x}_{p_1} \boldsymbol{x}_{p_1}^\top
\end{aligned}$$

where we have already calculated $\frac{\partial}{\partial \boldsymbol{\delta}_{p_1}} \mathrm{sigmoid}\left\{ \boldsymbol{\delta}_{p_1}^\top \boldsymbol{x}_{p_1} \right\}$ in subsection 3.3.1.

3.4.2 | Mixed derivative with respect to $\boldsymbol{\delta}_{p_1}$ and $\boldsymbol{\delta}_{p_2}$

$$\begin{aligned}
&\frac{\partial^2 l}{\partial \boldsymbol{\delta}_{p_2} \boldsymbol{\delta}_{p_1}} = \\
=&\frac{\partial}{\partial \boldsymbol{\delta}_{p_2}} \frac{\partial l}{\partial \boldsymbol{\delta}_{p_1}} \\
=&\frac{\partial}{\partial \boldsymbol{\delta}_{p_2}} \left(\frac{1}{f} \frac{\partial f}{\partial p_1} \frac{\partial p_1}{\partial \boldsymbol{\delta}_{p_1}} - \mathrm{diag}\left\{ \boldsymbol{\lambda}_{p_1} \right\} \boldsymbol{u}_{p_1} \right) \\
=& \left(\frac{\partial}{\partial \boldsymbol{\delta}_{p_2}} \frac{1}{f} \right) \frac{\partial f}{\partial p_1} \frac{\partial p_1}{\partial \boldsymbol{\delta}_{p_1}} + \frac{1}{f} \left(\frac{\partial}{\partial \boldsymbol{\delta}_{p_2}} \frac{\partial f}{\partial p_1} \right) \frac{\partial p_1}{\partial \boldsymbol{\delta}_{p_1}} + \frac{1}{f} \frac{\partial f}{\partial p_1} \left(\frac{\partial}{\partial \boldsymbol{\delta}_{p_2}} \frac{\partial p_1}{\partial \boldsymbol{\delta}_{p_1}} \right)
\end{aligned}$$

where

$$\frac{\partial}{\partial \boldsymbol{\delta}_{p_2}} \frac{\partial p_1}{\boldsymbol{\delta}_{p_1}} = 0$$

and

$$\frac{\partial}{\partial \boldsymbol{\delta}_{p_2}} \frac{1}{f} = \frac{-1}{f^2} \frac{\partial f}{\partial \boldsymbol{\delta}_{p_2}}$$

where $\frac{\partial f}{\partial \boldsymbol{\delta}_{p_2}}$ was already calculated in subsection 3.3.6, and

$$\frac{\partial}{\partial \boldsymbol{\delta}_{p_2}} \frac{\partial f}{\partial p_1} =$$

$$= \frac{\partial p_2}{\partial \boldsymbol{\delta}_{p_2}} \left(\frac{\partial}{\partial p_2} \frac{\partial f}{\partial p_1} \right) =$$

$$= \frac{\partial p_2}{\partial \boldsymbol{\delta}_{p_2}} \frac{\partial}{\partial p_2} \begin{cases} -(1-p_2) g(y_1, y_2 | \mu_1, \sigma_1, \mu_2, \sigma_2, \rho) & \text{if } y_1 \neq 0 \text{ and } y_2 \neq 0 \\ -(1-p_2) g(y_1, y_2 | \mu_1, \sigma_1, \mu_2, \sigma_2, \rho) - p_2 f_{\text{NB}}(y_1; \mu_1, \sigma_1) & \text{if } y_1 \neq 0 \text{ and } y_2 = 0 \\ -(1-p_2) g(y_1, y_2 | \mu_1, \sigma_1, \mu_2, \sigma_2, \rho) + (1-p_2) f_{\text{NB}}(y_2; \mu_2, \sigma_2) & \text{if } y_1 = 0 \text{ and } y_2 \neq 0 \\ -(1-p_2) g(y_1, y_2 | \mu_1, \sigma_1, \mu_2, \sigma_2, \rho) + p_2 - p_2 f_{\text{NB}}(y_1 ; \mu_1, \sigma_1) + (1-p_2) f_{\text{NB}}(y_2 ; \mu_2, \sigma_2) & \text{if } y_1 = 0 \text{ and } y_2 = 0 \end{cases}$$

$$= \frac{\partial p_2}{\partial \boldsymbol{\delta}_{p_2}} \begin{cases} g(y_1, y_2 | \mu_1, \sigma_1, \mu_2, \sigma_2, \rho) & \text{if } y_1 \neq 0 \text{ and } y_2 \neq 0 \\ g(y_1, y_2 | \mu_1, \sigma_1, \mu_2, \sigma_2, \rho) - f_{\text{NB}}(y_1; \mu_1, \sigma_1) & \text{if } y_1 \neq 0 \text{ and } y_2 = 0 \\ g(y_1, y_2 | \mu_1, \sigma_1, \mu_2, \sigma_2, \rho) - f_{\text{NB}}(y_2; \mu_2, \sigma_2) & \text{if } y_1 = 0 \text{ and } y_2 \neq 0 \\ g(y_1, y_2 | \mu_1, \sigma_1, \mu_2, \sigma_2, \rho) + 1 - f_{\text{NB}}(y_1; \mu_1, \sigma_1) - f_{\text{NB}}(y_2; \mu_2, \sigma_2) & \text{if } y_1 = 0 \text{ and } y_2 = 0 \end{cases}$$

3.4.3 | Mixed derivative with respect to $\boldsymbol{\delta}_{p_1}$ and $\boldsymbol{\delta}_{\mu_1}$

$$\frac{\partial^2 l}{\partial \boldsymbol{\delta}_{p_1} \partial \boldsymbol{\delta}_{\mu_1}} =$$

$$= \frac{\partial}{\partial \boldsymbol{\delta}_{p_1}} \left(\frac{\partial l}{\partial \boldsymbol{\delta}_{\mu_1}} \right) =$$

$$= \frac{\partial}{\partial \boldsymbol{\delta}_{p_1}} \left(\frac{1}{f} \frac{\partial f}{\partial \mu_1} \frac{\partial \mu_1}{\partial \boldsymbol{\delta}_{\mu_1}} - \text{diag}\left\{ \boldsymbol{\lambda}_{\mu_1} \right\} \boldsymbol{u}_{\mu_1} \right) =$$

$$= \left(\frac{\partial}{\partial \boldsymbol{\delta}_{p_1}} \frac{1}{f} \right) \frac{\partial f}{\partial \mu_1} \frac{\partial \mu_1}{\partial \boldsymbol{\delta}_{\mu_1}} + \frac{1}{f} \left(\frac{\partial}{\partial \boldsymbol{\delta}_{p_1}} \frac{\partial f}{\partial \mu_1} \right) \frac{\partial \mu_1}{\partial \boldsymbol{\delta}_{\mu_1}} + \frac{1}{f} \frac{\partial f}{\partial \mu_1} \left(\frac{\partial}{\partial \boldsymbol{\delta}_{p_1}} \frac{\partial \mu_1}{\partial \boldsymbol{\delta}_{\mu_1}} \right)$$

where we have calculated $\frac{\partial}{\partial \boldsymbol{\delta}_{p_1}} \frac{1}{f}$ in subsection 3.4.1 and where $\frac{\partial}{\partial \boldsymbol{\delta}_{p_1}} \frac{\partial \mu_1}{\partial \boldsymbol{\delta}_{\mu_1}} = 0$. The remaining term is:

$$\frac{\partial}{\partial \boldsymbol{\delta}_{p_1}} \frac{\partial f}{\partial \mu_1} = \frac{\partial p_1}{\partial \boldsymbol{\delta}_{p_1}} \frac{\partial}{\partial \mu_1} \frac{\partial f}{\partial p_1} =$$

$$= \frac{\partial p_1}{\partial \boldsymbol{\delta}_{p_1}} \begin{cases} -(1-p_2) \frac{\partial}{\partial \mu_1} g(y_1, y_2 | \mu_1, \sigma_1, \mu_2, \sigma_2, \rho) & \text{if } y_1 \neq 0 \text{ and } y_2 \neq 0 \\ -(1-p_2) \frac{\partial}{\partial \mu_1} g(y_1, y_2 | \mu_1, \sigma_1, \mu_2, \sigma_2, \rho) - p_2 \frac{\partial}{\partial \mu_1} f_{\text{NB}}(y_1; \mu_1, \sigma_1) & \text{if } y_1 \neq 0 \text{ and } y_2 = 0 \\ -(1-p_2) \frac{\partial}{\partial \mu_1} g(y_1, y_2 | \mu_1, \sigma_1, \mu_2, \sigma_2, \rho) & \text{if } y_1 = 0 \text{ and } y_2 \neq 0 \\ -(1-p_2) \frac{\partial}{\partial \mu_1} g(y_1, y_2 | \mu_1, \sigma_1, \mu_2, \sigma_2, \rho) - p_2 \frac{\partial}{\partial \mu_1} f_{\text{NB}}(y_1 ; \mu_1, \sigma_1) & \text{if } y_1 = 0 \text{ and } y_2 = 0 \end{cases}$$

3.4.4 | Mixed derivative with respect to $\boldsymbol{\delta}_{p_1}$ and $\boldsymbol{\delta}_{\mu_2}$

$$\frac{\partial^2 l}{\partial \boldsymbol{\delta}_{p_1} \partial \boldsymbol{\delta}_{\mu_2}} =$$

$$= \frac{\partial}{\partial \boldsymbol{\delta}_{p_1}} \left(\frac{\partial l}{\partial \boldsymbol{\delta}_{\mu_2}} \right) =$$

$$= \frac{\partial}{\partial \boldsymbol{\delta}_{p_1}} \left(\frac{1}{f} \frac{\partial f}{\partial \mu_2} \frac{\partial \mu_2}{\partial \boldsymbol{\delta}_{\mu_2}} - \text{diag}\left\{ \boldsymbol{\lambda}_{\mu_2} \right\} \boldsymbol{u}_{\mu_2} \right) =$$

$$= \left(\frac{\partial}{\partial \boldsymbol{\delta}_{p_1}} \frac{1}{f} \right) \frac{\partial f}{\partial \mu_2} \frac{\partial \mu_2}{\partial \boldsymbol{\delta}_{\mu_2}} + \frac{1}{f} \left(\frac{\partial}{\partial \boldsymbol{\delta}_{p_1}} \frac{\partial f}{\partial \mu_2} \right) \frac{\partial \mu_2}{\partial \boldsymbol{\delta}_{\mu_2}} + \frac{1}{f} \frac{\partial f}{\partial \mu_2} \left(\frac{\partial}{\partial \boldsymbol{\delta}_{p_1}} \frac{\partial \mu_2}{\partial \boldsymbol{\delta}_{\mu_2}} \right)$$

where we have calculated $\frac{\partial}{\partial \boldsymbol{\delta}_{p_1}} \frac{1}{f}$ in subsection 3.4.1 and where $\frac{\partial}{\partial \boldsymbol{\delta}_{p_1}} \frac{\partial \mu_2}{\partial \boldsymbol{\delta}_{\mu_2}} = 0$. The remaining term is:

$$\frac{\partial}{\partial \boldsymbol{\delta}_{p_1}}\frac{\partial f}{\partial \mu_2} = \frac{\partial p_1}{\partial \boldsymbol{\delta}_{p_1}}\frac{\partial}{\partial p_1}\frac{\partial f}{\partial \mu_2} =$$

$$=\frac{\partial p_1}{\partial \boldsymbol{\delta}_{p_1}}\begin{cases} -(1-p_2)\frac{\partial}{\partial \mu_2}g(y_1,y_2|\mu_1,\sigma_1,\mu_2,\sigma_2,\rho) & \text{if } y_1 \neq 0 \text{ and } y_2 \neq 0 \\ -(1-p_2)\frac{\partial}{\partial \mu_2}g(y_1,y_2|\mu_1,\sigma_1,\mu_2,\sigma_2,\rho) & \text{if } y_1 \neq 0 \text{ and } y_2 = 0 \\ -(1-p_2)\frac{\partial}{\partial \mu_2}g(y_1,y_2|\mu_1,\sigma_1,\mu_2,\sigma_2,\rho) + (1-p_2)\frac{\partial}{\partial \mu_2}f_{\mathrm{NB}}(y_2;\mu_2,\sigma_2) & \text{if } y_1 = 0 \text{ and } y_2 \neq 0 \\ -(1-p_2)\frac{\partial}{\partial \mu_2}g(y_1,y_2|\mu_1,\sigma_1,\mu_2,\sigma_2,\rho) + (1-p_2)\frac{\partial}{\partial \mu_2}f_{\mathrm{NB}}(y_2;\mu_2,\sigma_2) & \text{if } y_1 = 0 \text{ and } y_2 = 0 \end{cases}$$

3.4.5 | Mixed derivative with respect to $\boldsymbol{\delta}_{p_1}$ and $\boldsymbol{\delta}_{\sigma_1}$

$$\begin{aligned} &\frac{\partial^2 l}{\partial \boldsymbol{\delta}_{p_1}\partial \boldsymbol{\delta}_{\sigma_1}} = \\ &=\frac{\partial}{\partial \boldsymbol{\delta}_{p_1}}\left(\frac{\partial l}{\partial \boldsymbol{\delta}_{\sigma_1}}\right) = \\ &=\frac{\partial}{\partial \boldsymbol{\delta}_{p_1}}\left(\frac{1}{f}\frac{\partial f}{\partial \sigma_1}\frac{\partial \sigma_1}{\partial \boldsymbol{\delta}_{\sigma_1}} - \operatorname{diag}\left\{\boldsymbol{\lambda}_{\sigma_1}\right\}\boldsymbol{u}_{\sigma_1}\right) = \\ &=\left(\frac{\partial}{\partial \boldsymbol{\delta}_{p_1}}\frac{1}{f}\right)\frac{\partial f}{\partial \sigma_1}\frac{\partial \sigma_1}{\partial \boldsymbol{\delta}_{\sigma_1}} + \frac{1}{f}\left(\frac{\partial}{\partial \boldsymbol{\delta}_{p_1}}\frac{\partial f}{\partial \sigma_1}\right)\frac{\partial \sigma_1}{\partial \boldsymbol{\delta}_{\sigma_1}} + \frac{1}{f}\frac{\partial f}{\partial \sigma_1}\left(\frac{\partial}{\partial \boldsymbol{\delta}_{p_1}}\frac{\partial \sigma_1}{\partial \boldsymbol{\delta}_{\sigma_1}}\right) \end{aligned}$$

where we have calculated $\frac{\partial}{\partial \boldsymbol{\delta}_{p_1}}\frac{1}{f}$ in subsection 3.4.1 and where $\frac{\partial}{\partial \boldsymbol{\delta}_{p_1}}\frac{\partial \sigma_1}{\partial \boldsymbol{\delta}_{\sigma_1}} = 0$. The remaining term is:

$$\frac{\partial}{\partial \boldsymbol{\delta}_{p_1}}\frac{\partial f}{\partial \sigma_1} = \frac{\partial p_1}{\partial \boldsymbol{\delta}_{p_1}}\frac{\partial}{\partial p_1}\frac{\partial f}{\partial \sigma_1} =$$

$$=\frac{\partial p_1}{\partial \boldsymbol{\delta}_{p_1}}\begin{cases} -(1-p_2)\frac{\partial}{\partial \sigma_1}g(y_1,y_2|\mu_1,\sigma_1,\mu_2,\sigma_2,\rho) & \text{if } y_1 \neq 0 \text{ and } y_2 \neq 0 \\ -(1-p_2)\frac{\partial}{\partial \sigma_1}g(y_1,y_2|\mu_1,\sigma_1,\mu_2,\sigma_2,\rho) - p_2\frac{\partial}{\partial \sigma_1}f_{\mathrm{NB}}(y_1;\mu_1,\sigma_1) & \text{if } y_1 \neq 0 \text{ and } y_2 = 0 \\ -(1-p_2)\frac{\partial}{\partial \sigma_1}g(y_1,y_2|\mu_1,\sigma_1,\mu_2,\sigma_2,\rho) & \text{if } y_1 = 0 \text{ and } y_2 \neq 0 \\ -(1-p_2)\frac{\partial}{\partial \sigma_1}g(y_1,y_2|\mu_1,\sigma_1,\mu_2,\sigma_2,\rho) - p_2\frac{\partial}{\partial \sigma_1}f_{\mathrm{NB}}(y_1;\mu_1,\sigma_1) & \text{if } y_1 = 0 \text{ and } y_2 = 0 \end{cases}$$

3.4.6 | Mixed derivative with respect to $\boldsymbol{\delta}_{p_1}$ and $\boldsymbol{\delta}_{\sigma_2}$

$$\begin{aligned} &\frac{\partial^2 l}{\partial \boldsymbol{\delta}_{p_1}\partial \boldsymbol{\delta}_{\sigma_2}} = \\ &=\frac{\partial}{\partial \boldsymbol{\delta}_{p_1}}\left(\frac{\partial l}{\partial \boldsymbol{\delta}_{\sigma_2}}\right) = \\ &=\frac{\partial}{\partial \boldsymbol{\delta}_{p_1}}\left(\frac{1}{f}\frac{\partial f}{\partial \sigma_2}\frac{\partial \sigma_2}{\partial \boldsymbol{\delta}_{\sigma_2}} - \operatorname{diag}\left\{\boldsymbol{\lambda}_{\sigma_2}\right\}\boldsymbol{u}_{\sigma_2}\right) = \\ &=\left(\frac{\partial}{\partial \boldsymbol{\delta}_{p_1}}\frac{1}{f}\right)\frac{\partial f}{\partial \sigma_2}\frac{\partial \sigma_2}{\partial \boldsymbol{\delta}_{\sigma_2}} + \frac{1}{f}\left(\frac{\partial}{\partial \boldsymbol{\delta}_{p_1}}\frac{\partial f}{\partial \sigma_2}\right)\frac{\partial \sigma_2}{\partial \boldsymbol{\delta}_{\sigma_2}} + \frac{1}{f}\frac{\partial f}{\partial \sigma_2}\left(\frac{\partial}{\partial \boldsymbol{\delta}_{p_1}}\frac{\partial \sigma_2}{\partial \boldsymbol{\delta}_{\sigma_2}}\right) \end{aligned}$$

where we have calculated $\frac{\partial}{\partial \boldsymbol{\delta}_{p_1}}\frac{1}{f}$ in subsection 3.4.1 and where $\frac{\partial}{\partial \boldsymbol{\delta}_{p_1}}\frac{\partial \sigma_2}{\partial \boldsymbol{\delta}_{\sigma_2}} = 0$. The remaining term is:

$$\frac{\partial}{\partial \boldsymbol{\delta}_{p_1}}\frac{\partial f}{\partial \sigma_2} = \frac{\partial p_1}{\partial \boldsymbol{\delta}_{p_1}}\frac{\partial}{\partial p_1}\frac{\partial f}{\partial \sigma_2} =$$

$$=\frac{\partial p_1}{\partial \boldsymbol{\delta}_{p_1}}\begin{cases} -(1-p_2)\frac{\partial}{\partial \sigma_2}g(y_1,y_2|\mu_1,\sigma_1,\mu_2,\sigma_2,\rho) & \text{if } y_1 \neq 0 \text{ and } y_2 \neq 0 \\ -(1-p_2)\frac{\partial}{\partial \sigma_2}g(y_1,y_2|\mu_1,\sigma_1,\mu_2,\sigma_2,\rho) & \text{if } y_1 \neq 0 \text{ and } y_2 = 0 \\ -(1-p_2)\frac{\partial}{\partial \sigma_2}g(y_1,y_2|\mu_1,\sigma_1,\mu_2,\sigma_2,\rho) + (1-p_2)\frac{\partial}{\partial \sigma_2}f_{\mathrm{NB}}(y_2;\mu_2,\sigma_2) & \text{if } y_1 = 0 \text{ and } y_2 \neq 0 \\ -(1-p_2)\frac{\partial}{\partial \sigma_2}g(y_1,y_2|\mu_1,\sigma_1,\mu_2,\sigma_2,\rho) + (1-p_2)\frac{\partial}{\partial \sigma_2}f_{\mathrm{NB}}(y_2;\mu_2,\sigma_2) & \text{if } y_1 = 0 \text{ and } y_2 = 0 \end{cases}$$

3.4.7 | Mixed derivative with respect to $\boldsymbol{\delta}_{p_1}$ and $\boldsymbol{\delta}_{\rho}$

$$
\begin{aligned}
&\frac{\partial^2 l}{\partial \boldsymbol{\delta}_{p_1} \partial \boldsymbol{\delta}_{\rho}} = \\
=&\frac{\partial}{\partial \boldsymbol{\delta}_{p_1}} \left(\frac{\partial l}{\partial \boldsymbol{\delta}_{\rho}} \right) = \\
=&\frac{\partial}{\partial \boldsymbol{\delta}_{p_1}} \left(\frac{1}{f} \frac{\partial f}{\partial \rho} \frac{\partial \rho}{\partial \boldsymbol{\delta}_{\rho}} - \operatorname{diag}\left\{ \boldsymbol{\lambda}_{\rho} \right\} \boldsymbol{u}_{\rho} \right) = \\
=&\left(\frac{\partial}{\partial \boldsymbol{\delta}_{p_1}} \frac{1}{f} \right) \frac{\partial f}{\partial \rho} \frac{\partial \rho}{\partial \boldsymbol{\delta}_{\rho}} + \frac{1}{f} \left(\frac{\partial}{\partial \boldsymbol{\delta}_{p_1}} \frac{\partial f}{\partial \rho} \right) \frac{\partial \rho}{\partial \boldsymbol{\delta}_{\rho}} + \frac{1}{f} \frac{\partial f}{\partial \rho} \left(\frac{\partial}{\partial \boldsymbol{\delta}_{p_1}} \frac{\partial \rho}{\partial \boldsymbol{\delta}_{\rho}} \right)
\end{aligned}
$$

where we have calculated $\frac{\partial}{\partial \boldsymbol{\delta}_{p_1}} \frac{1}{f}$ in subsection 3.4.1 and where $\frac{\partial}{\partial \boldsymbol{\delta}_{p_1}} \frac{\partial \rho}{\partial \boldsymbol{\delta}_{\rho}} = 0$. The remaining term is:

$$
\begin{aligned}
&\frac{\partial}{\partial \boldsymbol{\delta}_{p_1}} \frac{\partial f}{\partial \rho} = \frac{\partial p_1}{\partial \boldsymbol{\delta}_{p_1}} \frac{\partial}{\partial p_1} \frac{\partial f}{\partial \rho} = \\
=&\frac{\partial p_1}{\partial \boldsymbol{\delta}_{p_1}} \begin{cases} -(1-p_2) \frac{\partial}{\partial \rho} g(y_1, y_2 | \mu_1, \sigma_1, \mu_2, \sigma_2, \rho) & \text{if } y_1 \neq 0, y_2 \neq 0 \\ -(1-p_2) \frac{\partial}{\partial \rho} g(y_1, y_2 | \mu_1, \sigma_1, \mu_2, \sigma_2, \rho) & \text{if } y_1 \neq 0, y_2 = 0 \\ -(1-p_2) \frac{\partial}{\partial \rho} g(y_1, y_2 | \mu_1, \sigma_1, \mu_2, \sigma_2, \rho) & \text{if } y_1 = 0, y_2 \neq 0 \\ -(1-p_2) \frac{\partial}{\partial \rho} g(y_1, y_2 | \mu_1, \sigma_1, \mu_2, \sigma_2, \rho) & \text{if } y_1 = 0, y_2 = 0 \end{cases}
\end{aligned}
$$

3.4.8 | 2nd derivative with respect to $\boldsymbol{\delta}_{\mu_1}$

$$
\begin{aligned}
&\frac{\partial^2 l}{\partial \boldsymbol{\delta}_{\mu_1}^2} = \\
=&\frac{\partial}{\partial \boldsymbol{\delta}_{\mu_1}} \left(\frac{\partial l}{\partial \boldsymbol{\delta}_{\mu_1}} \right) = \\
=&\frac{\partial}{\partial \boldsymbol{\delta}_{\mu_1}} \left(\frac{1}{f} \frac{\partial f}{\partial \mu_1} \frac{\partial \mu_1}{\partial \boldsymbol{\delta}_{\mu_1}} - \operatorname{diag}\left\{ \boldsymbol{\lambda}_{\mu_1} \right\} \boldsymbol{u}_{\mu_1} \right) = \\
=&\left(\frac{\partial}{\partial \boldsymbol{\delta}_{\mu_1}} \frac{1}{f} \right) \frac{\partial f}{\partial \mu_1} \frac{\partial \mu_1}{\partial \boldsymbol{\delta}_{\mu_1}} + \frac{1}{f} \left(\frac{\partial}{\partial \boldsymbol{\delta}_{\mu_1}} \frac{\partial f}{\partial \mu_1} \right) \frac{\partial \mu_1}{\partial \boldsymbol{\delta}_{\mu_1}} + \frac{1}{f} \frac{\partial f}{\partial \mu_1} \left(\frac{\partial}{\partial \boldsymbol{\delta}_{\mu_1}} \frac{\partial \mu_1}{\partial \boldsymbol{\delta}_{\mu_1}} \right) - \operatorname{diag}\left\{ \boldsymbol{\lambda}_{\mu_1} \right\}
\end{aligned}
$$

where

$$
\begin{aligned}
&\frac{\partial}{\partial \boldsymbol{\delta}_{\mu_1}} \frac{\partial \mu_1}{\partial \boldsymbol{\delta}_{\mu_1}} = \\
=&\frac{\partial}{\partial \boldsymbol{\delta}_{\mu_1}} \exp\left\{ \boldsymbol{\delta}_{\mu_1}^\top \boldsymbol{x}_{\mu_1} \right\} \boldsymbol{x}_{\mu_1} \\
=&\exp\left\{ \boldsymbol{\delta}_{\mu_1}^\top \boldsymbol{x}_{\mu_1} \right\} \boldsymbol{x}_{\mu_1} \boldsymbol{x}_{\mu_1}^\top
\end{aligned}
$$

and

$$
\frac{\partial}{\partial \boldsymbol{\delta}_{\mu_1}} \frac{1}{f} = \frac{-1}{f^2} \left(\frac{\partial f}{\partial \boldsymbol{\delta}_{\mu_1}} \right)
$$

where $\frac{\partial f}{\partial \boldsymbol{\delta}_{\mu_1}}$ was already calculated in subsection 3.3.3, and

$$
\begin{aligned}
&\frac{\partial}{\partial \boldsymbol{\delta}_{\mu_1}} \frac{\partial f}{\partial \mu_1} = \\
=&\frac{\partial \mu_1}{\partial \boldsymbol{\delta}_{\mu_1}} \frac{\partial}{\partial \mu_1} \frac{\partial f}{\partial \mu_1} = \\
=&\frac{\partial \mu_1}{\partial \boldsymbol{\delta}_{\mu_1}} \begin{cases} (1-p_1)(1-p_2)\frac{\partial^2}{\partial \mu_1^2} g(y_1, y_2|\mu_1, \sigma_1, \mu_2, \sigma_2, \rho) & \text{if } y_1 \neq 0, y_2 \neq 0 \\ (1-p_1)(1-p_2)\frac{\partial^2}{\partial \mu_1^2} g(y_1, y_2|\mu_1, \sigma_1, \mu_2, \sigma_2, \rho) + (1-p_1)p_2 \frac{\partial^2}{\partial \mu_1^2} f_{\mathrm{NB}}(y_1; \mu_1, \sigma_1) & \text{if } y_1 \neq 0, y_2 = 0 \\ (1-p_1)(1-p_2)\frac{\partial^2}{\partial \mu_1^2} g(y_1, y_2|\mu_1, \sigma_1, \mu_2, \sigma_2, \rho) & \text{if } y_1 = 0, y_2 \neq 0 \\ (1-p_1)(1-p_2)\frac{\partial^2}{\partial \mu_1^2} g(y_1, y_2|\mu_1, \sigma_1, \mu_2, \sigma_2, \rho) + (1-p_1)p_2 \frac{\partial^2}{\partial \mu_1^2} f_{\mathrm{NB}}(y_1; \mu_1, \sigma_1) & \text{if } y_1 = 0, y_2 = 0 \end{cases}
\end{aligned}
$$

where

$$
\begin{aligned}
&\frac{\partial^2}{\partial \mu_1^2} g(y_1, y_2|\mu_1, \sigma_1, \mu_2, \sigma_2, \rho) = \\
=&\frac{\partial}{\partial \mu_1} \left(\frac{\partial}{\partial \mu_1} g(y_1, y_2|\mu_1, \sigma_1, \mu_2, \sigma_2, \rho) \right) = \\
=&\frac{\partial}{\partial \mu_1} \left(\frac{\partial F_{\mathrm{NB}}(y_1; \mu_1, \sigma_1)}{\partial \mu_1} \frac{\partial g(y_1, y_2|\mu_1, \sigma_1, \mu_2, \sigma_2, \rho)}{\partial F_{\mathrm{NB}}(y_1; \mu_1, \sigma_1)} + \frac{\partial F_{\mathrm{NB}}(y_1-1; \mu_1, \sigma_1)}{\partial \mu_1} \frac{\partial g(y_1, y_2|\mu_1, \sigma_1, \mu_2, \sigma_2, \rho)}{\partial F_{\mathrm{NB}}(y_1-1; \mu_1, \sigma_1)} \right) = \\
=&\left(\frac{\partial^2 F_{\mathrm{NB}}(y_1; \mu_1, \sigma_1)}{\partial \mu_1^2} \right) \frac{\partial g(y_1, y_2|\mu_1, \sigma_1, \mu_2, \sigma_2, \rho)}{\partial F_{\mathrm{NB}}(y_1; \mu_1, \sigma_1)} + \\
&+ \frac{\partial F_{\mathrm{NB}}(y_1; \mu_1, \sigma_1)}{\partial \mu_1} \left(\frac{\partial}{\partial \mu_1} \frac{\partial g(y_1, y_2|\mu_1, \sigma_1, \mu_2, \sigma_2, \rho)}{\partial F_{\mathrm{NB}}(y_1; \mu_1, \sigma_1)} \right) + \\
&+ \left(\frac{\partial^2 F_{\mathrm{NB}}(y_1-1; \mu_1, \sigma_1)}{\partial \mu_1^2} \right) \frac{\partial g(y_1, y_2|\mu_1, \sigma_1, \mu_2, \sigma_2, \rho)}{\partial F_{\mathrm{NB}}(y_1-1; \mu_1, \sigma_1)} + \\
&+ \frac{\partial F_{\mathrm{NB}}(y_1-1; \mu_1, \sigma_1)}{\partial \mu_1} \left(\frac{\partial}{\partial \mu_1} \frac{\partial g(y_1, y_2|\mu_1, \sigma_1, \mu_2, \sigma_2, \rho)}{\partial F_{\mathrm{NB}}(y_1-1; \mu_1, \sigma_1)} \right)
\end{aligned}
$$

where

$$
\begin{aligned}
\frac{\partial^2 F_{\mathrm{NB}}(y_1; \mu_1, \sigma_1)}{\partial \mu_1^2} &= \sum_{k=0}^{y_1} \frac{\partial^2 f_{\mathrm{NB}}(y_1; \mu_1, \sigma_1)}{\partial \mu_1^2} = \\
&= \sum_{k=0}^{y_1} \frac{\Gamma(k + \frac{1}{\sigma_1})}{\Gamma(1+k)\Gamma(\frac{1}{\sigma_1})} \left(\frac{\frac{1}{\sigma_1}}{\frac{1}{\sigma_1} + \mu_1} \right)^{\frac{1}{\sigma_1}} \left(\frac{\mu_1}{\frac{1}{\sigma_1} + \mu_1} \right)^k \times \\
&\qquad \left[\frac{\frac{1}{\sigma_1} \left[k^2 \frac{1}{\sigma_1} + \mu_1(1 + \frac{1}{\sigma_1}) - k(\frac{1}{\sigma_1} + 2\mu_1(1 + \frac{1}{\sigma_1})) \right]}{\mu_1^2(\mu_1 + \frac{1}{\sigma_1})^2} \right]
\end{aligned}
$$

and

$$
\begin{aligned}
&\frac{\partial}{\partial \mu_1} \frac{\partial g(y_1, y_2|\mu_1, \sigma_1, \mu_2, \sigma_2, \rho)}{\partial F_{\mathrm{NB}}(y_1; \mu_1, \sigma_1)} = \\
=&\frac{\partial F_{\mathrm{NB}}(y_1; \mu_1, \sigma_1)}{\partial \mu_1} \left(\frac{\partial^2 g(y_1, y_2|\mu_1, \sigma_1, \mu_2, \sigma_2, \rho)}{\partial F_{\mathrm{NB}}^2(y_1; \mu_1, \sigma_1)} \right) + \\
+&\frac{\partial F_{\mathrm{NB}}(y_1-1; \mu_1, \sigma_1)}{\partial \mu_1} \left(\frac{\partial^2 g(y_1, y_2|\mu_1, \sigma_1, \mu_2, \sigma_2, \rho)}{\partial F_{\mathrm{NB}}(y_1-1; \mu_1, \sigma_1) \partial F_{\mathrm{NB}}(y_1; \mu_1, \sigma_1)} \right)
\end{aligned}
$$

where

$$\begin{aligned}
&\frac{\partial^2 g(y_1, y_2|\mu_1, \sigma_1, \mu_2, \sigma_2, \rho)}{\partial F^2_{\text{NB}}(y_1; \mu_1, \sigma_1)} = \\
=&\frac{\rho}{\phi(\Phi^{-1}(F_{\text{NB}}(y_1; \mu_1, \sigma_1)))\sqrt{1-[\rho]^2}} \times \\
&\times \left[\phi\left(\frac{\Phi^{-1}(F_{\text{NB}}(y_2; \mu_2, \sigma) - \Phi^{-1}(F_{\text{NB}}(y_1; \mu_1, \sigma_1))\rho}{\sqrt{1-[\rho]^2}}\right) + \right.\\
&\left. - \phi\left(\frac{\Phi^{-1}(F_{\text{NB}}(y_2 - 1; \mu_2, \sigma_2)) - \Phi^{-1}(F_{\text{NB}}(y_1; \mu_1, \sigma_1))\rho}{\sqrt{1-[\rho]^2}}\right)\right]
\end{aligned}$$

and

$$\frac{\partial^2 g(y_1, y_2|\mu_1, \sigma_1, \mu_2, \sigma_2, \rho)}{\partial F_{\text{NB}}(y_1 - 1; \mu_1, \sigma_1)\partial F_{\text{NB}}(y_1; \mu_1, \sigma_1)} = 0$$

similarly,

$$\begin{aligned}
&\frac{\partial}{\partial \mu_1}\frac{\partial g(y_1, y_2|\mu_1, \sigma_1, \mu_2, \sigma_2, \rho)}{\partial F_{\text{NB}}(y_1 - 1; \mu_1, \sigma_1)} = \\
=&\frac{\partial F_{\text{NB}}(y_1; \mu_1, \sigma_1)}{\partial \mu_1}\left(\frac{\partial^2 g(y_1, y_2|\mu_1, \sigma_1, \mu_2, \sigma_2, \rho)}{\partial F_{\text{NB}}(y_1; \mu_1, \sigma_1)\partial F_{\text{NB}}(y_1 - 1; \mu_1, \sigma_1)}\right) + \\
+&\frac{\partial F_{\text{NB}}(y_1 - 1; \mu_1, \sigma_1)}{\partial \mu_1}\left(\frac{\partial^2 g(y_1, y_2|\mu_1, \sigma_1, \mu_2, \sigma_2, \rho)}{\partial F^2_{\text{NB}}(y_1 - 1; \mu_1, \sigma_1)}\right)
\end{aligned}$$

where

$$\begin{aligned}
&\frac{\partial^2 g(y_1, y_2|\mu_1, \sigma_1, \mu_2, \sigma_2, \rho)}{\partial F^2_{\text{NB}}(y_1 - 1; \mu_1, \sigma_1)} = \\
=&\frac{\rho}{\phi\left(\Phi^{-1}(F_{\text{NB}}(y_1 - 1; \mu_1, \sigma_1))\right)\sqrt{1-[\rho]^2}} \times \\
&\times \left[\phi\left(\frac{\Phi^{-1}(F_{\text{NB}}(y_2; \mu_2, \sigma_2)) - \Phi^{-1}(F_{\text{NB}}(y_1 - 1; \mu_1, \sigma_1))\rho}{\sqrt{1-[\rho]^2}}\right) + \right.\\
&\left. - \phi\left(\frac{\Phi^{-1}(F_{\text{NB}}(y_2 - 1; \mu_2, \sigma_2)) - \Phi^{-1}(F_{\text{NB}}(y_1 - 1; \mu_1, \sigma_1))\rho}{\sqrt{1-[\rho]^2}}\right)\right]
\end{aligned}$$

3.4.9 | Mixed derivative with respect to $\boldsymbol{\delta}_{\mu_1}$ and $\boldsymbol{\delta}_{\mu_2}$

$$\begin{aligned}
&\frac{\partial^2 l}{\partial \boldsymbol{\delta}_{\mu_2}\partial \boldsymbol{\delta}_{\mu_1}} = \\
=&\frac{\partial}{\partial \boldsymbol{\delta}_{\mu_2}}\left(\frac{\partial l}{\partial \boldsymbol{\delta}_{\mu_1}}\right) = \\
=&\frac{\partial}{\partial \boldsymbol{\delta}_{\mu_2}}\left(\frac{1}{f}\frac{\partial f}{\partial \mu_1}\frac{\partial \mu_1}{\partial \boldsymbol{\delta}_{\mu_1}} - \text{diag}\left\{\boldsymbol{\lambda}_{\mu_1}\right\}\boldsymbol{u}_{\mu_1}\right) = \\
=&\left(\frac{\partial}{\partial \boldsymbol{\delta}_{\mu_2}}\frac{1}{f}\right)\frac{\partial f}{\partial \mu_1}\frac{\partial \mu_1}{\partial \boldsymbol{\delta}_{\mu_1}} + \frac{1}{f}\left(\frac{\partial}{\partial \boldsymbol{\delta}_{\mu_2}}\frac{\partial f}{\partial \mu_1}\right)\frac{\partial \mu_1}{\partial \boldsymbol{\delta}_{\mu_1}} + \frac{1}{f}\frac{\partial f}{\partial \mu_1}\left(\frac{\partial}{\partial \boldsymbol{\delta}_{\mu_2}}\frac{\partial \mu_1}{\partial \boldsymbol{\delta}_{\mu_1}}\right)
\end{aligned}$$

where

$$\begin{aligned}
&\frac{\partial}{\partial \boldsymbol{\delta}_{\mu_2}}\frac{\partial \mu_1}{\partial \boldsymbol{\delta}_{\mu_1}} = \\
=&\frac{\partial}{\partial \boldsymbol{\delta}_{\mu_2}}\exp\left\{\boldsymbol{\delta}^\top_{\mu_1}\boldsymbol{x}_{\mu_1}\right\}\boldsymbol{x}_{\mu_1} \\
=&0
\end{aligned}$$

and

$$\frac{\partial}{\partial \boldsymbol{\delta}_{\mu_2}} \frac{1}{f} = \frac{-1}{f^2} \left(\frac{\partial f}{\partial \boldsymbol{\delta}_{\mu_2}} \right)$$

where $\frac{\partial f}{\partial \boldsymbol{\delta}_{\mu_2}}$ was already calculated in subsection 3.3.4, and

$$\begin{aligned}
&\frac{\partial}{\partial \boldsymbol{\delta}_{\mu_2}} \frac{\partial f}{\partial \mu_1} = \\
=&\frac{\partial \mu_2}{\partial \boldsymbol{\delta}_{\mu_2}} \frac{\partial}{\partial \mu_2} \frac{\partial f}{\partial \mu_1} = \\
=&\frac{\partial \mu_2}{\partial \boldsymbol{\delta}_{\mu_2}} \begin{cases} (1-p_1)(1-p_2)\frac{\partial^2}{\partial \mu_2 \partial \mu_1} g(y_1, y_2|\mu_1, \sigma_1, \mu_2, \sigma_2, \rho) & \text{if ...} \\ (1-p_1)(1-p_2)\frac{\partial^2}{\partial \mu_2 \partial \mu_1} g(y_1, y_2|\mu_1, \sigma_1, \mu_2, \sigma_2, \rho) + (1-p_1)p_2 \frac{\partial^2}{\partial \mu_2 \partial \mu_1} f_{\mathrm{NB}}(y_1; \mu_1, \sigma_1) & \text{if ...} \\ (1-p_1)(1-p_2)\frac{\partial^2}{\partial \mu_2 \partial \mu_1} g(y_1, y_2|\mu_1, \sigma_1, \mu_2, \sigma_2, \rho) & \text{if ...} \\ (1-p_1)(1-p_2)\frac{\partial^2}{\partial \mu_2 \partial \mu_1} g(y_1, y_2|\mu_1, \sigma_1, \mu_2, \sigma_2, \rho) + (1-p_1)p_2 \frac{\partial^2}{\partial \mu_2 \partial \mu_1} f_{\mathrm{NB}}(y_1; \mu_1, \sigma_1) & \text{if ...} \end{cases}
\end{aligned}$$

where

$$\frac{\partial^2}{\partial \mu_2 \partial \mu_1} f_{\mathrm{NB}}(y_1; \mu_1, \sigma_1) = 0$$

and

$$\begin{aligned}
&\frac{\partial^2}{\partial \mu_2 \partial \mu_1} g(y_1, y_2|\mu_1, \sigma_1, \mu_2, \sigma_2, \rho) = \\
=&\frac{\partial}{\partial \mu_2} \left(\frac{\partial}{\partial \mu_1} g(y_1, y_2|\mu_1, \sigma_1, \mu_2, \sigma_2, \rho) \right) = \\
=&\frac{\partial}{\partial \mu_2} \left(\frac{\partial F_{\mathrm{NB}}(y_1; \mu_1, \sigma_1)}{\partial \mu_1} \frac{\partial g(y_1, y_2|\mu_1, \sigma_1, \mu_2, \sigma_2, \rho)}{\partial F_{\mathrm{NB}}(y_1; \mu_1, \sigma_1)} + \frac{\partial F_{\mathrm{NB}}(y_1-1; \mu_1, \sigma_1)}{\partial \mu_1} \frac{\partial g(y_1, y_2|\mu_1, \sigma_1, \mu_2, \sigma_2, \rho)}{\partial F_{\mathrm{NB}}(y_1-1; \mu_1, \sigma_1)} \right) = \\
=&\left(\frac{\partial}{\partial \mu_2} \frac{\partial F_{\mathrm{NB}}(y_1; \mu_1, \sigma_1)}{\partial \mu_1} \right) \frac{\partial g(y_1, y_2|\mu_1, \sigma_1, \mu_2, \sigma_2, \rho)}{\partial F_{\mathrm{NB}}(y_1; \mu_1, \sigma_1)} + \\
&+ \frac{\partial F_{\mathrm{NB}}(y_1; \mu_1, \sigma_1)}{\partial \mu_1} \left(\frac{\partial}{\partial \mu_2} \frac{\partial g(y_1, y_2|\mu_1, \sigma_1, \mu_2, \sigma_2, \rho)}{\partial F_{\mathrm{NB}}(y_1; \mu_1, \sigma_1)} \right) + \\
&+ \left(\frac{\partial}{\partial \mu_2} \frac{\partial F_{\mathrm{NB}}(y_1-1; \mu_1, \sigma_1)}{\partial \mu_1} \right) \frac{\partial g(y_1, y_2|\mu_1, \sigma_1, \mu_2, \sigma_2, \rho)}{\partial F_{\mathrm{NB}}(y_1-1; \mu_1, \sigma_1)} + \\
&+ \frac{\partial F_{\mathrm{NB}}(y_1-1; \mu_1, \sigma_1)}{\partial \mu_1} \left(\frac{\partial}{\partial \mu_2} \frac{\partial g(y_1, y_2|\mu_1, \sigma_1, \mu_2, \sigma_2, \rho)}{\partial F_{\mathrm{NB}}(y_1-1; \mu_1, \sigma_1)} \right) = \\
=&\frac{\partial F_{\mathrm{NB}}(y_1; \mu_1, \sigma_1)}{\partial \mu_1} \left(\frac{\partial}{\partial \mu_2} \frac{\partial g(y_1, y_2|\mu_1, \sigma_1, \mu_2, \sigma_2, \rho)}{\partial F_{\mathrm{NB}}(y_1; \mu_1, \sigma_1)} \right) + \\
+&\frac{\partial F_{\mathrm{NB}}(y_1-1; \mu_1, \sigma_1)}{\partial \mu_1} \left(\frac{\partial}{\partial \mu_2} \frac{\partial g(y_1, y_2|\mu_1, \sigma_1, \mu_2, \sigma_2, \rho)}{\partial F_{\mathrm{NB}}(y_1-1; \mu_1, \sigma_1)} \right)
\end{aligned}$$

where

$$\begin{aligned}
&\frac{\partial}{\partial \mu_2} \frac{\partial g(y_1, y_2|\mu_1, \sigma_1, \mu_2, \sigma_2, \rho)}{\partial F_{\mathrm{NB}}(y_1; \mu_1, \sigma_1)} = \\
=&\frac{\partial F_{\mathrm{NB}}(y_2; \mu_2, \sigma_2)}{\partial \mu_2} \left(\frac{\partial^2 g(y_1, y_2|\mu_1, \sigma_1, \mu_2, \sigma_2, \rho)}{\partial F_{\mathrm{NB}}(y_2; \mu_2, \sigma_2) \partial F_{\mathrm{NB}}(y_1; \mu_1, \sigma_1)} \right) + \\
&+ \frac{\partial F_{\mathrm{NB}}(y_2-1; \mu_2, \sigma_2)}{\partial \mu_2} \left(\frac{\partial^2 g(y_1, y_2|\mu_1, \sigma_1, \mu_2, \sigma_2, \rho)}{\partial F_{\mathrm{NB}}(y_2-1; \mu_2, \sigma_2) \partial F_{\mathrm{NB}}(y_1; \mu_1, \sigma_1)} \right)
\end{aligned}$$

and

$$\frac{\partial}{\partial \mu_2} \frac{\partial g(y_1, y_2 | \mu_1, \sigma_1, \mu_2, \sigma_2, \rho)}{\partial F_{\text{NB}}(y_1 - 1; \mu_1, \sigma_1)} =$$
$$= \frac{\partial F_{\text{NB}}(y_2; \mu_2, \sigma_2)}{\partial \mu_2} \left(\frac{\partial^2 g(y_1, y_2 | \mu_1, \sigma_1, \mu_2, \sigma_2, \rho)}{\partial F_{\text{NB}}(y_2; \mu_2, \sigma_2) \partial F_{\text{NB}}(y_1 - 1; \mu_1, \sigma_1)} \right) +$$
$$+ \frac{\partial F_{\text{NB}}(y_2 - 1; \mu_2, \sigma_2)}{\partial \mu_2} \left(\frac{\partial^2 g(y_1, y_2 | \mu_1, \sigma_1, \mu_2, \sigma_2, \rho)}{\partial F_{\text{NB}}(y_2 - 1; \mu_2, \sigma_2) \partial F_{\text{NB}}(y_1 - 1; \mu_1, \sigma_1)} \right)$$

where

$$\frac{\partial^2 g(y_1, y_2 | \mu_1, \sigma_1, \mu_2, \sigma_2, \rho)}{\partial F_{\text{NB}}(y_2; \mu_2, \sigma_2) \partial F_{\text{NB}}(y_1; \mu_1, \sigma_1)} =$$
$$= \phi \left(\frac{\Phi^{-1}(F_{\text{NB}}(y_2; \mu_2, \sigma_2)) - \Phi^{-1}(F_{\text{NB}}(y_1; \mu_1, \sigma_1))\rho}{\sqrt{1 - [\rho]^2}} \right) \frac{1}{\phi(\Phi^{-1}(F_{\text{NB}}(y_2; \mu_2, \sigma_2))) \sqrt{1 - [\rho]^2}}$$

and

$$\frac{\partial^2 g(y_1, y_2 | \mu_1, \sigma_1, \mu_2, \sigma_2, \rho)}{\partial F_{\text{NB}}(y_2 - 1; \mu_2, \sigma_2) \partial F_{\text{NB}}(y_1; \mu_1, \sigma_1)} =$$
$$= - \phi \left(\frac{\Phi^{-1}(F_{\text{NB}}(y_1 - 1; \mu_1, \sigma_1)) - \Phi^{-1}(F_{\text{NB}}(y_2; \mu_2, \sigma_2))\rho}{\sqrt{1 - [\rho]^2}} \right) \frac{1}{\phi(\Phi^{-1}(F_{\text{NB}}(y_1 - 1; \mu_1, \sigma_1))) \sqrt{1 - [\rho]^2}}$$

and

$$\frac{\partial^2 g(y_1, y_2 | \mu_1, \sigma_1, \mu_2, \sigma_2, \rho)}{\partial F_{\text{NB}}(y_2; \mu_2, \sigma_2) \partial F_{\text{NB}}(y_1 - 1; \mu_1, \sigma_1)} =$$
$$= - \phi \left(\frac{\Phi^{-1}(F_{\text{NB}}(y_2; \mu_2, \sigma_2)) - \Phi^{-1}(F_{\text{NB}}(y_1 - 1; \mu_1, \sigma_1))\rho}{\sqrt{1 - [\rho]^2}} \right) \frac{1}{\phi \left(\Phi^{-1}(F_{\text{NB}}(y_2; \mu_2, \sigma_2)) \right) \sqrt{1 - [\rho]^2}}$$

and

$$\frac{\partial^2 g(y_1, y_2 | \mu_1, \sigma_1, \mu_2, \sigma_2, \rho)}{\partial F_{\text{NB}}(y_2 - 1; \mu_2, \sigma_2) \partial F_{\text{NB}}(y_1 - 1; \mu_1, \sigma_1)} =$$
$$= \phi \left(\frac{\Phi^{-1}(F_{\text{NB}}(y_2 - 1; \mu_2, \sigma_2)) - \Phi^{-1}(F_{\text{NB}}(y_1 - 1; \mu_1, \sigma_1))\rho}{\sqrt{1 - [\rho]^2}} \right) \frac{1}{\phi \left(\Phi^{-1}(F_{\text{NB}}(y_2 - 1; \mu_2, \sigma_2)) \right) \sqrt{1 - [\rho]^2}}$$

3.4.10 | 2nd derivative with respect to $\boldsymbol{\delta}_{\sigma_1}$

$$\frac{\partial^2 l}{\partial \boldsymbol{\delta}_{\sigma_1}^2} =$$
$$= \frac{\partial}{\partial \boldsymbol{\delta}_{\sigma_1}} \left(\frac{\partial l}{\partial \boldsymbol{\delta}_{\sigma_1}} \right) =$$
$$= \frac{\partial}{\partial \boldsymbol{\delta}_{\sigma_1}} \left(\frac{1}{f} \frac{\partial f}{\partial \sigma_1} \frac{\partial \sigma_1}{\partial \boldsymbol{\delta}_{\sigma_1}} - \text{diag} \left\{ \boldsymbol{\lambda}_{\sigma_1} \right\} \boldsymbol{u}_{\sigma_1} \right) =$$
$$= \left(\frac{\partial}{\partial \boldsymbol{\delta}_{\sigma_1}} \frac{1}{f} \right) \frac{\partial f}{\partial \sigma_1} \frac{\partial \sigma_1}{\partial \boldsymbol{\delta}_{\sigma_1}} + \frac{1}{f} \left(\frac{\partial}{\partial \boldsymbol{\delta}_{\sigma_1}} \frac{\partial f}{\partial \sigma_1} \right) \frac{\partial \sigma_1}{\partial \boldsymbol{\delta}_{\sigma_1}} + \frac{1}{f} \frac{\partial f}{\partial \sigma_1} \left(\frac{\partial}{\partial \boldsymbol{\delta}_{\sigma_1}} \frac{\partial \sigma_1}{\partial \boldsymbol{\delta}_{\sigma_1}} \right) - \text{diag} \left\{ \boldsymbol{\lambda}_{\sigma_1} \right\}$$

where

$$\frac{\partial}{\partial \boldsymbol{\delta}_{\sigma_1}} \frac{\partial \sigma_1}{\partial \boldsymbol{\delta}_{\sigma_1}} =$$
$$= \frac{\partial}{\partial \boldsymbol{\delta}_{\sigma_1}} \exp \left\{ \boldsymbol{\delta}_{\sigma_1}^\top \boldsymbol{x}_{\sigma_1} \right\} \boldsymbol{x}_{\sigma_1}$$
$$= \exp \left\{ \boldsymbol{\delta}_{\sigma_1}^\top \boldsymbol{x}_{\sigma_1} \right\} \boldsymbol{x}_{\sigma_1} \boldsymbol{x}_{\sigma_1}^\top$$

and

$$\frac{\partial}{\partial \boldsymbol{\delta}_{\sigma_1}} \frac{1}{f} = \frac{-1}{f^2} \left(\frac{\partial f}{\partial \boldsymbol{\delta}_{\sigma_1}} \right)$$

where $\frac{\partial f}{\partial \boldsymbol{\delta}_{\sigma_1}}$ was already calculated in subsection 3.3.5, and

$$\begin{aligned}
&\frac{\partial}{\partial \boldsymbol{\delta}_{\sigma_1}} \frac{\partial f}{\partial \sigma_1} = \\
&= \frac{\partial \sigma_1}{\partial \boldsymbol{\delta}_{\sigma_1}} \frac{\partial}{\partial \sigma_1} \frac{\partial f}{\partial \sigma_1} = \\
&= \frac{\partial \sigma_1}{\partial \boldsymbol{\delta}_{\sigma_1}} \begin{cases} (1-p_1)(1-p_2)\frac{\partial^2}{\partial \sigma_1^2} g(y_1, y_2|\mu_1, \sigma_1, \mu_2, \sigma_2, \rho) & \text{if } y_1 \neq 0, y_2 \neq 0 \\ (1-p_1)(1-p_2)\frac{\partial^2}{\partial \sigma_1^2} g(y_1, y_2|\mu_1, \sigma_1, \mu_2, \sigma_2, \rho) + (1-p_1)p_2 \frac{\partial^2}{\partial \sigma_1^2} f_{\text{NB}}(y_1; \mu_1, \sigma_1) & \text{if } y_1 \neq 0, y_2 = 0 \\ (1-p_1)(1-p_2)\frac{\partial^2}{\partial \sigma_1^2} g(y_1, y_2|\mu_1, \sigma_1, \mu_2, \sigma_2, \rho) & \text{if } y_1 = 0, y_2 \neq 0 \\ (1-p_1)(1-p_2)\frac{\partial^2}{\partial \sigma_1^2} g(y_1, y_2|\mu_1, \sigma_1, \mu_2, \sigma_2, \rho) + (1-p_1)p_2 \frac{\partial^2}{\partial \sigma_1^2} f_{\text{NB}}(y_1; \mu_1, \sigma_1) & \text{if } y_1 = 0, y_2 = 0 \end{cases}
\end{aligned}$$

where

$$\begin{aligned}
&\frac{\partial^2}{\partial \sigma_1^2} F_{\text{NB}}(y_1; \mu_1, \sigma_1) = \sum_{k=0}^{y_1} \frac{\partial^2}{\partial \sigma_1^2} f_{\text{NB}}(y_1; \mu_1, \sigma_1) = \\
&= \sum_{k=0}^{y_1} \frac{\partial}{\partial \sigma_1} \frac{-1}{\sigma_1^2} \frac{\Gamma(k + \frac{1}{\sigma_1})}{\Gamma(1+k)\Gamma(\frac{1}{\sigma_1})(\frac{1}{\sigma_1} + \mu_1)} \left(\frac{\frac{1}{\sigma_1}}{\frac{1}{\sigma_1} + \mu_1} \right)^{\frac{1}{\sigma_1}} \left(\frac{}{\frac{1}{\sigma_1} + \mu_1} \right)^{k} \\
&\times \left(-k + \mu_1 + \left[\frac{1}{\sigma_1} + \mu_1 \right] \left[\log \left(\frac{\frac{1}{\sigma_1}}{\frac{1}{\sigma_1} + \mu_1} \right) + \psi(k + \frac{1}{\sigma_1}) - \psi(\frac{1}{\sigma_1}) \right] \right)
\end{aligned}$$

and

$$\begin{aligned}
&\frac{\partial^2}{\partial \sigma_1^2} g(y_1, y_2|\mu_1, \sigma_1, \mu_2, \sigma_2, \rho) = \\
&= \frac{\partial}{\partial \sigma_1} \left(\frac{\partial g(y_1, y_2|\mu_1, \sigma_1, \mu_2, \sigma_2, \rho)}{\partial \sigma_1} \right) = \\
&= \frac{\partial}{\partial \sigma_1} \left(\frac{\partial g(y_1, y_2|\mu_1, \sigma_1, \mu_2, \sigma_2, \rho)}{\partial F_{\text{NB}}(y_1; \mu_1, \sigma_1)} \frac{\partial F_{\text{NB}}(y_1; \mu_1, \sigma_1)}{\partial \sigma_1} + \frac{\partial g(y_1, y_2|\mu_1, \sigma_1, \mu_2, \sigma_2, \rho)}{\partial F_{\text{NB}}(y_1 - 1; \mu_1, \sigma_1)} \frac{\partial F_{\text{NB}}(y_1 - 1; \mu_1, \sigma_1)}{\partial \sigma_1} \right) = \\
&= \left(\frac{\partial}{\partial \sigma_1} \frac{\partial g(y_1, y_2|\mu_1, \sigma_1, \mu_2, \sigma_2, \rho)}{\partial F_{\text{NB}}(y_1; \mu_1, \sigma_1)} \right) \frac{\partial F_{\text{NB}}(y_1; \mu_1, \sigma_1)}{\partial \sigma_1} + \\
&+ \frac{\partial g(y_1, y_2|\mu_1, \sigma_1, \mu_2, \sigma_2, \rho)}{\partial F_{\text{NB}}(y_1; \mu_1, \sigma_1)} \left(\frac{\partial^2 F_{\text{NB}}(y_1; \mu_1, \sigma_1)}{\partial \sigma_1^2} \right) + \\
&+ \left(\frac{\partial}{\partial \sigma_1} \frac{\partial g(y_1, y_2|\mu_1, \sigma_1, \mu_2, \sigma_2, \rho)}{\partial F_{\text{NB}}(y_1 - 1; \mu_1, \sigma_1)} \right) \frac{\partial F_{\text{NB}}(y_1 - 1; \mu_1, \sigma_1)}{\partial \sigma_1} + \\
&+ \frac{\partial g(y_1, y_2|\mu_1, \sigma_1, \mu_2, \sigma_2, \rho)}{\partial F_{\text{NB}}(y_1 - 1; \mu_1, \sigma_1)} \left(\frac{\partial^2 F_{\text{NB}}(y_1 - 1; \mu_1, \sigma_1)}{\partial \sigma_1^2} \right)
\end{aligned}$$

where

$$\begin{aligned}
&\frac{\partial}{\partial \sigma_1} \frac{\partial g(y_1, y_2|\mu_1, \sigma_1, \mu_2, \sigma_2, \rho)}{\partial F_{\text{NB}}(y_1; \mu_1, \sigma_1)} = \\
&= \frac{\partial F_{\text{NB}}(y_1; \mu_1, \sigma_1)}{\partial \sigma_1} \left(\frac{\partial^2 g(y_1, y_2|\mu_1, \sigma_1, \mu_2, \sigma_2, \rho)}{\partial F_{\text{NB}}^2(y_1; \mu_1, \sigma_1)} \right) + \\
&+ \frac{\partial F_{\text{NB}}(y_1 - 1; \mu_1, \sigma_1)}{\partial \sigma_1} \left(\frac{\partial^2 g(y_1, y_2|\mu_1, \sigma_1, \mu_2, \sigma_2, \rho)}{\partial F_{\text{NB}}(y_1 - 1; \mu_1, \sigma_1) \partial F_{\text{NB}}(y_1; \mu_1, \sigma_1)} \right)
\end{aligned}$$

and

$$\frac{\partial}{\partial \sigma_1}\frac{\partial g(y_1, y_2|\mu_1, \sigma_1, \mu_2, \sigma_2, \rho)}{\partial F_{\text{NB}}(y_1-1;\mu_1,\sigma_1)} =$$
$$=\frac{\partial F_{\text{NB}}(y_1;\mu_1,\sigma_1)}{\partial \sigma_1}\left(\frac{\partial^2 g(y_1, y_2|\mu_1, \sigma_1, \mu_2, \sigma_2, \rho)}{\partial F_{\text{NB}}(y_1;\mu_1,\sigma_1)\partial F_{\text{NB}}(y_1-1;\mu_1,\sigma_1)}\right)+$$
$$+\frac{\partial F_{\text{NB}}(y_1-1;\mu_1,\sigma_1)}{\partial \sigma_1}\left(\frac{\partial^2 g(y_1, y_2|\mu_1, \sigma_1, \mu_2, \sigma_2, \rho)}{\partial F^2_{\text{NB}}(y_1-1;\mu_1,\sigma_1)}\right)$$

and all those quantities have been calculated previously in the document.

3.4.11 | Mixed derivative with respect to $\boldsymbol{\delta}_{\sigma_1}$ and $\boldsymbol{\delta}_{\sigma_2}$

$$\frac{\partial^2 l}{\partial \boldsymbol{\delta}_{\sigma_2}\partial \boldsymbol{\delta}_{\sigma_1}} =$$
$$=\frac{\partial}{\partial \boldsymbol{\delta}_{\sigma_2}}\left(\frac{\partial l}{\partial \boldsymbol{\delta}_{\sigma_1}}\right)=$$
$$=\frac{\partial}{\partial \boldsymbol{\delta}_{\sigma_2}}\left(\frac{1}{f}\frac{\partial f}{\partial \sigma_1}\frac{\partial \sigma_1}{\partial \boldsymbol{\delta}_{\sigma_1}} - \text{diag}\left\{\boldsymbol{\lambda}_{\sigma_1}\right\}\boldsymbol{u}_{\sigma_1}\right)=$$
$$=\left(\frac{\partial}{\partial \boldsymbol{\delta}_{\sigma_2}}\frac{1}{f}\right)\frac{\partial f}{\partial \sigma_1}\frac{\partial \sigma_1}{\partial \boldsymbol{\delta}_{\sigma_1}}+\frac{1}{f}\left(\frac{\partial}{\partial \boldsymbol{\delta}_{\sigma_2}}\frac{\partial f}{\partial \sigma_1}\right)\frac{\partial \sigma_1}{\partial \boldsymbol{\delta}_{\sigma_1}}+\frac{1}{f}\frac{\partial f}{\partial \sigma_1}\left(\frac{\partial}{\partial \boldsymbol{\delta}_{\sigma_2}}\frac{\partial \sigma_1}{\partial \boldsymbol{\delta}_{\sigma_1}}\right)$$

where

$$\frac{\partial}{\partial \boldsymbol{\delta}_{\sigma_2}}\frac{\partial \sigma_1}{\partial \boldsymbol{\delta}_{\sigma_1}} = 0$$

and

$$\frac{\partial}{\partial \boldsymbol{\delta}_{\sigma_2}}\frac{1}{f} = \frac{-1}{f^2_{\text{NB,NB}}}\left(\frac{\partial f}{\partial \boldsymbol{\delta}_{\sigma_2}}\right)$$

where $\frac{\partial f}{\partial \boldsymbol{\delta}_{\sigma_2}}$ was calculated in 3.3.6, and

$$\frac{\partial}{\partial \boldsymbol{\delta}_{\sigma_2}}\frac{\partial f}{\partial \sigma_1} =$$
$$=\frac{\partial \sigma_2}{\partial \boldsymbol{\delta}_{\sigma_2}}\frac{\partial}{\partial \sigma_2}\frac{\partial f}{\partial \sigma_1} =$$
$$=\frac{\partial \sigma_2}{\partial \boldsymbol{\delta}_{\sigma_2}}\begin{cases}(1-p_1)(1-p_2)\frac{\partial^2}{\partial \sigma_2 \sigma_1}g(y_1, y_2|\mu_1, \sigma_1, \mu_2, \sigma_2, \rho) & \text{if ...}\\ (1-p_1)(1-p_2)\frac{\partial^2}{\partial \sigma_2 \sigma_1}g(y_1, y_2|\mu_1, \sigma_1, \mu_2, \sigma_2, \rho)+(1-p_1)p_2\frac{\partial^2}{\partial \sigma_2 \sigma_1}f_{\text{NB}}(y_1;\mu_1,\sigma_1) & \text{if ...}\\ (1-p_1)(1-p_2)\frac{\partial^2}{\partial \sigma_2 \sigma_1}g(y_1, y_2|\mu_1, \sigma_1, \mu_2, \sigma_2, \rho) & \text{if ...}\\ (1-p_1)(1-p_2)\frac{\partial^2}{\partial \sigma_2 \sigma_1}g(y_1, y_2|\mu_1, \sigma_1, \mu_2, \sigma_2, \rho)+(1-p_1)p_2\frac{\partial^2}{\partial \sigma_2 \sigma_1}f_{\text{NB}}(y_1;\mu_1,\sigma_1) & \text{if ...}\end{cases}$$

where $(1-p_1)p_2\frac{\partial^2}{\partial \sigma_2 \sigma_1}f_{\text{NB}}(y_1;\mu_1,\sigma_1) = 0$, and

$$\frac{\partial^2}{\partial\sigma_2\sigma_1}g(y_1,y_2|\mu_1,\sigma_1,\mu_2,\sigma_2,\rho)=$$
$$=\frac{\partial}{\partial\sigma_2}\left(\frac{\partial}{\partial\sigma_1}g(y_1,y_2|\mu_1,\sigma_1,\mu_2,\sigma_2,\rho)\right)=$$
$$=\frac{\partial}{\partial\sigma_2}\left(\frac{\partial g(y_1,y_2|\mu_1,\sigma_1,\mu_2,\sigma_2,\rho)}{F_{\mathrm{NB}}(y_1;\mu_1,\sigma_1)}\frac{\partial F_{\mathrm{NB}}(y_1;\mu_1,\sigma_1)}{\partial\sigma_1}+\frac{\partial g(y_1,y_2|\mu_1,\sigma_1,\mu_2,\sigma_2,\rho)}{\partial F_{\mathrm{NB}}(y_1-1;\mu_1,\sigma_1)}\frac{\partial F_{\mathrm{NB}}(y_1-1;\mu_1,\sigma_1)}{\partial\sigma_1}\right)=$$
$$=\left(\frac{\partial}{\partial\sigma_2}\frac{\partial g(y_1,y_2|\mu_1,\sigma_1,\mu_2,\sigma_2,\rho)}{F_{\mathrm{NB}}(y_1;\mu_1,\sigma_1)}\right)\frac{\partial F_{\mathrm{NB}}(y_1;\mu_1,\sigma_1)}{\partial\sigma_1}+$$
$$+\frac{\partial g(y_1,y_2|\mu_1,\sigma_1,\mu_2,\sigma_2,\rho)}{F_{\mathrm{NB}}(y_1;\mu_1,\sigma_1)}\left(\frac{\partial}{\partial\sigma_2}\frac{\partial F_{\mathrm{NB}}(y_1;\mu_1,\sigma_1)}{\partial\sigma_1}\right)+$$
$$+\left(\frac{\partial}{\partial\sigma_2}\frac{\partial g(y_1,y_2|\mu_1,\sigma_1,\mu_2,\sigma_2,\rho)}{\partial F_{\mathrm{NB}}(y_1-1;\mu_1,\sigma_1)}\right)\frac{\partial F_{\mathrm{NB}}(y_1-1;\mu_1,\sigma_1)}{\partial\sigma_1}+$$
$$+\frac{\partial g(y_1,y_2|\mu_1,\sigma_1,\mu_2,\sigma_2,\rho)}{\partial F_{\mathrm{NB}}(y_1-1;\mu_1,\sigma_1)}\left(\frac{\partial}{\partial\sigma_2}\frac{\partial F_{\mathrm{NB}}(y_1-1;\mu_1,\sigma_1)}{\partial\sigma_1}\right)=$$
$$=\left(\frac{\partial}{\partial\sigma_2}\frac{\partial g(y_1,y_2|\mu_1,\sigma_1,\mu_2,\sigma_2,\rho)}{F_{\mathrm{NB}}(y_1;\mu_1,\sigma_1)}\right)\frac{\partial F_{\mathrm{NB}}(y_1;\mu_1,\sigma_1)}{\partial\sigma_1}+$$
$$+\left(\frac{\partial}{\partial\sigma_2}\frac{\partial g(y_1,y_2|\mu_1,\sigma_1,\mu_2,\sigma_2,\rho)}{\partial F_{\mathrm{NB}}(y_1-1;\mu_1,\sigma_1)}\right)\frac{\partial F_{\mathrm{NB}}(y_1-1;\mu_1,\sigma_1)}{\partial\sigma_1}$$

where

$$\frac{\partial}{\partial\sigma_2}\frac{\partial g(y_1,y_2|\mu_1,\sigma_1,\mu_2,\sigma_2,\rho)}{\partial F_{\mathrm{NB}}(y_1;\mu_1,\sigma_1)}=$$
$$=\frac{\partial F_{\mathrm{NB}}(y_2;\mu_2,\sigma_2)}{\partial\sigma_2}\left(\frac{\partial}{\partial F_{\mathrm{NB}}(y_2;\mu_2,\sigma_2)}\frac{\partial g(y_1,y_2|\mu_1,\sigma_1,\mu_2,\sigma_2,\rho)}{\partial F_{\mathrm{NB}}(y_1;\mu_1,\sigma_1)}\right)+$$
$$+\frac{\partial F_{\mathrm{NB}}(y_2-1;\mu_2,\sigma_2)}{\partial\sigma_2}\left(\frac{\partial}{\partial F_{\mathrm{NB}}(y_2-1;\mu_2,\sigma_2)}\frac{\partial g(y_1,y_2|\mu_1,\sigma_1,\mu_2,\sigma_2,\rho)}{\partial F_{\mathrm{NB}}(y_1;\mu_1,\sigma_1)}\right)$$

and

$$\frac{\partial}{\partial\sigma_2}\frac{\partial g(y_1,y_2|\mu_1,\sigma_1,\mu_2,\sigma_2,\rho)}{\partial F_{\mathrm{NB}}(y_1-1;\mu_1,\sigma_1)}=$$
$$=\frac{\partial F_{\mathrm{NB}}(y_2;\mu_2,\sigma_2)}{\partial\sigma_2}\left(\frac{\partial}{\partial F_{\mathrm{NB}}(y_2;\mu_2,\sigma_2)}\frac{\partial g(y_1,y_2|\mu_1,\sigma_1,\mu_2,\sigma_2,\rho)}{\partial F_{\mathrm{NB}}(y_1-1;\mu_1,\sigma_1)}\right)+$$
$$+\frac{\partial F_{\mathrm{NB}}(y_2-1;\mu_2,\sigma_2)}{\partial\sigma_2}\left(\frac{\partial}{\partial F_{\mathrm{NB}}(y_2-1;\mu_2,\sigma_2)}\frac{\partial g(y_1,y_2|\mu_1,\sigma_1,\mu_2,\sigma_2,\rho)}{\partial F_{\mathrm{NB}}(y_1-1;\mu_1,\sigma_1)}\right)$$

These quantities have all been calculated previously.

3.4.12 | Mixed derivative with respect to $\boldsymbol{\delta}_{\sigma_1}$ and $\boldsymbol{\delta}_{\mu_1}$

$$\frac{\partial^2 l}{\partial\boldsymbol{\delta}_{\sigma_1}\partial\boldsymbol{\delta}_{\mu_1}}=$$
$$=\frac{\partial}{\partial\boldsymbol{\delta}_{\sigma_1}}\left(\frac{\partial l}{\partial\boldsymbol{\delta}_{\mu_1}}\right)=$$
$$=\frac{\partial}{\partial\boldsymbol{\delta}_{\sigma_1}}\left(\frac{1}{f}\frac{\partial f}{\partial\mu_1}\frac{\partial\mu_1}{\partial\boldsymbol{\delta}_{\mu_1}}-\mathrm{diag}\left\{\boldsymbol{\lambda}_{\mu_1}\right\}\boldsymbol{u}_{\mu_1}\right)=$$
$$=\left(\frac{\partial}{\partial\boldsymbol{\delta}_{\sigma_1}}\frac{1}{f}\right)\frac{\partial f}{\partial\,\mu_1}\frac{\partial\mu_1}{\partial\boldsymbol{\delta}_{\mu_1}}+\frac{1}{f}\left(\frac{\partial}{\partial\boldsymbol{\delta}_{\sigma_1}}\frac{\partial f}{\partial\mu_1}\right)\frac{\partial\mu_1}{\partial\boldsymbol{\delta}_{\mu_1}}+\frac{1}{f}\frac{\partial f}{\partial\mu_1}\left(\frac{\partial}{\partial\boldsymbol{\delta}_{\sigma_1}}\frac{\partial\mu_1}{\partial\boldsymbol{\delta}_{\mu_1}}\right)$$

where

$$\frac{\partial}{\partial\boldsymbol{\delta}_{\sigma_1}}\frac{\partial\mu_1}{\partial\boldsymbol{\delta}_{\mu_1}}=0$$

and

$$\frac{\partial}{\partial \boldsymbol{\delta}_{\sigma_1}} \frac{1}{f} = \frac{-1}{f^2} \left(\frac{\partial f}{\partial \boldsymbol{\delta}_{\sigma_1}} \right)$$

where $\frac{\partial f}{\partial \boldsymbol{\delta}_{\sigma_1}}$ was calculated in 3.3.5, and

$$\begin{aligned}
&\frac{\partial}{\partial \boldsymbol{\delta}_{\sigma_1}} \frac{\partial f}{\partial \mu_1} = \\
=&\frac{\partial \sigma_1}{\partial \boldsymbol{\delta}_{\sigma_1}} \left(\frac{\partial}{\partial \sigma_1} \frac{\partial f}{\partial \mu_1} \right) = \\
=&\frac{\partial \sigma_1}{\partial \boldsymbol{\delta}_{\sigma_1}} \begin{cases}
(1-p_1)(1-p_2)\frac{\partial^2}{\partial \sigma_1 \partial \mu_1} g(y_1, y_2 | \mu_1, \sigma_1, \mu_2, \sigma_2, \rho) & \text{if ...} \\
(1-p_1)(1-p_2)\frac{\partial^2}{\partial \sigma_1 \partial \mu_1} g(y_1, y_2 | \mu_1, \sigma_1, \mu_2, \sigma_2, \rho) + (1-p_1)p_2 \frac{\partial^2}{\partial \sigma_1 \partial \mu_1} f_{\mathrm{NB}}(y_1; \mu_1, \sigma_1) & \text{if ...} \\
(1-p_1)(1-p_2)\frac{\partial^2}{\partial \sigma_1 \partial \mu_1} g(y_1, y_2 | \mu_1, \sigma_1, \mu_2, \sigma_2, \rho) & \text{if ...} \\
(1-p_1)(1-p_2)\frac{\partial^2}{\partial \sigma_1 \partial \mu_1} g(y_1, y_2 | \mu_1, \sigma_1, \mu_2, \sigma_2, \rho) + (1-p_1)p_2 \frac{\partial^2}{\partial \sigma_1 \partial \mu_1} f_{\mathrm{NB}}(y_1; \mu_1, \sigma_1) & \text{if ...}
\end{cases}
\end{aligned}$$

where

$$\begin{aligned}
&\frac{\partial^2}{\partial \sigma_1 \partial \mu_1} f_{\mathrm{NB}}(y_1; \mu_1, \sigma_1) = \\
=&\frac{\partial}{\partial \sigma_1} \left(\frac{\partial f_{\mathrm{NB}}(y_1; \mu_1, \sigma_1)}{\partial \mu_1} \right) = \\
=&\frac{\partial}{\partial \sigma_1} \left(\frac{\Gamma(y_1 + \frac{1}{\sigma_1})}{\Gamma(1+y_1)\Gamma(\frac{1}{\sigma_1})} \left(\frac{\frac{1}{\sigma_1}}{\frac{1}{\sigma_1} + \mu_1} \right)^{\frac{1}{\sigma_1}} \left(\frac{\mu_1}{\frac{1}{\sigma_1} + \mu_1} \right)^{y_1} \times \left[\frac{y_1}{\mu_1} - \frac{\frac{1}{\sigma_1} + y_1}{\frac{1}{\sigma_1} + \mu_1} \right] \right)
\end{aligned}$$

and

$$\begin{aligned}
&\frac{\partial^2}{\partial \sigma_1 \partial \mu_1} g(y_1, y_2 | \mu_1, \sigma_1, \mu_2, \sigma_2, \rho) = \\
=&\frac{\partial}{\partial \sigma_1} \left(\frac{\partial}{\partial \mu_1} g(y_1, y_2 | \mu_1, \sigma_1, \mu_2, \sigma_2, \rho) \right) \\
=&\frac{\partial}{\partial \sigma_1} \left(\frac{\partial g(y_1, y_2 | \mu_1, \sigma_1, \mu_2, \sigma_2, \rho)}{F_{\mathrm{NB}}(y_1; \mu_1, \sigma_1)} \frac{\partial F_{\mathrm{NB}}(y_1; \mu_1, \sigma_1)}{\partial \mu_1} + \right. \\
&\left. + \frac{\partial g(y_1, y_2 | \mu_1, \sigma_1, \mu_2, \sigma_2, \rho)}{\partial F_{\mathrm{NB}}(y_1 - 1; \mu_1, \sigma_1)} \frac{\partial F_{\mathrm{NB}}(y_1 - 1; \mu_1, \sigma_1)}{\partial \mu_1} \right) = \\
=&\left(\frac{\partial}{\partial \sigma_1} \frac{\partial g(y_1, y_2 | \mu_1, \sigma_1, \mu_2, \sigma_2, \rho)}{F_{\mathrm{NB}}(y_1; \mu_1, \sigma_1)} \right) \frac{\partial F_{\mathrm{NB}}(y_1; \mu_1, \sigma_1)}{\partial \mu_1} + \\
&+ \frac{\partial g(y_1, y_2 | \mu_1, \sigma_1, \mu_2, \sigma_2, \rho)}{F_{\mathrm{NB}}(y_1; \mu_1, \sigma_1)} \left(\frac{\partial}{\partial \sigma_1} \frac{\partial F_{\mathrm{NB}}(y_1; \mu_1, \sigma_1)}{\partial \mu_1} \right) + \\
&+ \left(\frac{\partial}{\partial \sigma_1} \frac{\partial g(y_1, y_2 | \mu_1, \sigma_1, \mu_2, \sigma_2, \rho)}{F_{\mathrm{NB}}(y_1 - 1; \mu_1, \sigma_1)} \right) \frac{\partial F_{\mathrm{NB}}(y_1 - 1; \mu_1, \sigma_1)}{\partial \mu_1} + \\
&+ \frac{\partial g(y_1, y_2 | \mu_1, \sigma_1, \mu_2, \sigma_2, \rho)}{F_{\mathrm{NB}}(y_1 - 1; \mu_1, \sigma_1)} \left(\frac{\partial}{\partial \sigma_1} \frac{\partial F_{\mathrm{NB}}(y_1 - 1; \mu_1, \sigma_1)}{\partial \mu_1} \right)
\end{aligned}$$

where $\frac{\partial}{\partial \sigma_1} \frac{\partial g(y_1, y_2 | \mu_1, \sigma_1, \mu_2, \sigma_2, \rho)}{F_{\mathrm{NB}}(y_1; \mu_1, \sigma_1)}$ and $\frac{\partial}{\partial \sigma_1} \frac{\partial g(y_1, y_2 | \mu_1, \sigma_1, \mu_2, \sigma_2, \rho)}{F_{\mathrm{NB}}(y_1 - 1; \mu_1, \sigma_1)}$ were calculated in 3.4.10, and

$$\frac{\partial}{\partial \sigma_1} \frac{\partial F_{\mathrm{NB}}(y_1; \mu_1, \sigma_1)}{\partial \mu_1} = \sum_{k=0}^{y_1} \frac{\partial}{\partial \sigma_1} \frac{\partial f_{\mathrm{NB}}(k; \mu_1, \sigma_1)}{\partial \mu_1}$$

and we calculated the summand above previously in this section.

3.4.13 | Mixed derivative with respect to δ_{σ_1} and δ_{μ_2}

$$
\begin{aligned}
&\frac{\partial^2 l}{\partial \boldsymbol{\delta}_{\sigma_1} \partial \boldsymbol{\delta}_{\mu_2}} = \\
=&\frac{\partial}{\partial \boldsymbol{\delta}_{\sigma_1}} \left(\frac{\partial l}{\partial \boldsymbol{\delta}_{\mu_2}} \right) = \\
=&\frac{\partial}{\partial \boldsymbol{\delta}_{\sigma_1}} \left(\frac{1}{f} \frac{\partial f}{\partial \mu_2} \frac{\partial \mu_2}{\partial \boldsymbol{\delta}_{\mu_2}} - \operatorname{diag}\left\{ \boldsymbol{\lambda}_{\mu_2} \right\} \boldsymbol{u}_{\mu_2} \right) = \\
=&\left(\frac{\partial}{\partial \boldsymbol{\delta}_{\sigma_1}} \frac{1}{f} \right) \frac{\partial f}{\partial \mu_2} \frac{\partial \mu_2}{\partial \boldsymbol{\delta}_{\mu_2}} + \frac{1}{f} \left(\frac{\partial}{\partial \boldsymbol{\delta}_{\sigma_1}} \frac{\partial f}{\partial \mu_2} \right) \frac{\partial \mu_2}{\partial \boldsymbol{\delta}_{\mu_2}} + \frac{1}{f} \frac{\partial f}{\partial \mu_2} \left(\frac{\partial}{\partial \boldsymbol{\delta}_{\sigma_1}} \frac{\partial \mu_2}{\partial \boldsymbol{\delta}_{\mu_2}} \right)
\end{aligned}
$$

where

$$
\frac{\partial}{\partial \boldsymbol{\delta}_{\sigma_1}} \frac{\partial \mu_2}{\partial \boldsymbol{\delta}_{\mu_2}} = 0
$$

and

$$
\frac{\partial}{\partial \boldsymbol{\delta}_{\sigma_1}} \frac{1}{f} = \frac{-1}{f^2} \left(\frac{\partial f}{\partial \boldsymbol{\delta}_{\sigma_1}} \right)
$$

where $\frac{\partial f}{\partial \boldsymbol{\delta}_{\sigma_1}}$ was calculated in 3.3.5, and

$$
\begin{aligned}
&\frac{\partial}{\partial \boldsymbol{\delta}_{\sigma_1}} \frac{\partial f}{\partial \mu_2} = \\
=&\frac{\partial \sigma_1}{\partial \boldsymbol{\delta}_{\sigma_1}} \left(\frac{\partial}{\partial \sigma_1} \frac{\partial f}{\partial \mu_2} \right) = \\
=&\frac{\partial \sigma_1}{\partial \boldsymbol{\delta}_{\sigma_1}} \begin{cases}
(1-p_1)(1-p_2) \frac{\partial^2}{\partial \sigma_1 \partial \mu_2} g(y_1, y_2 | \mu_1, \sigma_1, \mu_2, \sigma_2, \rho) & \text{if ...} \\
(1-p_1)(1-p_2) \frac{\partial^2}{\partial \sigma_1 \partial \mu_2} g(y_1, y_2 | \mu_1, \sigma_1, \mu_2, \sigma_2, \rho) & \text{if ...} \\
(1-p_1)(1-p_2) \frac{\partial^2}{\partial \sigma_1 \partial \mu_2} g(y_1, y_2 | \mu_1, \sigma_1, \mu_2, \sigma_2, \rho) + p_1(1-p_2) \frac{\partial^2}{\partial \sigma_1 \partial \mu_2} f_{\text{NB}}(y_2; \mu_2, \sigma_2) & \text{if ...} \\
(1-p_1)(1-p_2) \frac{\partial^2}{\partial \sigma_1 \partial \mu_2} g(y_1, y_2 | \mu_1, \sigma_1, \mu_2, \sigma_2, \rho) + p_1(1-p_2) \frac{\partial^2}{\partial \sigma_1 \partial \mu_2} f_{\text{NB}}(y_2; \mu_2, \sigma_2) & \text{if ...}
\end{cases}
\end{aligned}
$$

where

$$
\frac{\partial^2}{\partial \sigma_1 \partial \mu_2} f_{\text{NB}}(y_2; \mu_2, \sigma_2) = 0
$$

and

$$
\begin{aligned}
&\frac{\partial^2}{\partial\sigma_1\partial\mu_2} g(y_1, y_2|\mu_1, \sigma_1, \mu_2, \sigma_2, \rho) = \\
=&\frac{\partial}{\partial\sigma_1}\left(\frac{\partial}{\partial\mu_2} g(y_1, y_2|\mu_1, \sigma_1, \mu_2, \sigma_2, \rho)\right) = \\
=&\frac{\partial}{\partial\sigma_1}\left(\frac{\partial g(y_1, y_2|\mu_1, \sigma_1, \mu_2, \sigma_2, \rho)}{F_{\text{NB}}(y_2; \mu_2, \sigma_2)} \frac{\partial F_{\text{NB}}(y_2; \mu_2, \sigma_2)}{\partial\mu_2} + \right.\\
+&\left.\frac{\partial g(y_1, y_2|\mu_1, \sigma_1, \mu_2, \sigma_2, \rho)}{\partial F_{\text{NB}}(y_2 - 1; \mu_2, \sigma_2)} \frac{\partial F_{\text{NB}}(y_2 - 1; \mu_2, \sigma_2)}{\partial\mu_2}\right) = \\
=&\left(\frac{\partial}{\partial\sigma_1}\frac{\partial g(y_1, y_2|\mu_1, \sigma_1, \mu_2, \sigma_2, \rho)}{F_{\text{NB}}(y_2; \mu_2, \sigma_2)}\right) \frac{\partial F_{\text{NB}}(y_2; \mu_2, \sigma_2)}{\partial\mu_2} + \\
+&\frac{\partial g(y_1, y_2|\mu_1, \sigma_1, \mu_2, \sigma_2, \rho)}{F_{\text{NB}}(y_2; \mu_2, \sigma_2)}\left(\frac{\partial}{\partial\sigma_1}\frac{\partial F_{\text{NB}}(y_2; \mu_2, \sigma_1)}{\partial\mu_2}\right) + \\
+&\left(\frac{\partial}{\partial\sigma_1}\frac{\partial g(y_1, y_2|\mu_1, \sigma_1, \mu_2, \sigma_2, \rho)}{F_{\text{NB}}(y_2 - 1; \mu_2, \sigma_2)}\right) \frac{\partial F_{\text{NB}}(y_2 - 1; \mu_2, \sigma_2)}{\partial\mu_2} + \\
+&\frac{\partial g(y_1, y_2|\mu_1, \sigma_1, \mu_2, \sigma_2, \rho)}{F_{\text{NB}}(y_2 - 1; \mu_2, \sigma_2)}\left(\frac{\partial}{\partial\sigma_1}\frac{\partial F_{\text{NB}}(y_2 - 1; \mu_2, \sigma_2)}{\partial\mu_2}\right) = \\
=&\left(\frac{\partial}{\partial\sigma_1}\frac{\partial g(y_1, y_2|\mu_1, \sigma_1, \mu_2, \sigma_2, \rho)}{F_{\text{NB}}(y_2; \mu_2, \sigma_2)}\right) \frac{\partial F_{\text{NB}}(y_2; \mu_2, \sigma_2)}{\partial\mu_2} + \\
+&\left(\frac{\partial}{\partial\sigma_1}\frac{\partial g(y_1, y_2|\mu_1, \sigma_1, \mu_2, \sigma_2, \rho)}{F_{\text{NB}}(y_2 - 1; \mu_2, \sigma_2)}\right) \frac{\partial F_{\text{NB}}(y_2 - 1; \mu_2, \sigma_2)}{\partial\mu_2}
\end{aligned}
$$

where

$$
\begin{aligned}
&\frac{\partial}{\partial\sigma_1}\frac{\partial g(y_1, y_2|\mu_1, \sigma_1, \mu_2, \sigma_2, \rho)}{F_{\text{NB}}(y_2; \mu_2, \sigma_2)} = \\
=&\frac{F_{\text{NB}}(y_1; \mu_1, \sigma_1)}{\partial\sigma_1}\frac{\partial^2 g(y_1, y_2|\mu_1, \sigma_1, \mu_2, \sigma_2, \rho)}{F_{\text{NB}}(y_1; \mu_1, \sigma_1)F_{\text{NB}}(y_2; \mu_2, \sigma_2)} + \\
+&\frac{F_{\text{NB}}(y_1 - 1; \mu_1, \sigma_1)}{\partial\sigma_1}\frac{\partial^2 g(y_1, y_2|\mu_1, \sigma_1, \mu_2, \sigma_2, \rho)}{F_{\text{NB}}(y_1 - 1; \mu_1, \sigma_1)F_{\text{NB}}(y_2; \mu_2, \sigma_2)}
\end{aligned}
$$

and

$$
\begin{aligned}
&\frac{\partial}{\partial\sigma_1}\frac{\partial g(y_1, y_2|\mu_1, \sigma_1, \mu_2, \sigma_2, \rho)}{F_{\text{NB}}(y_2 - 1; \mu_2, \sigma_2)} = \\
=&\frac{F_{\text{NB}}(y_1; \mu_1, \sigma_1)}{\partial\sigma_1}\frac{\partial^2 g(y_1, y_2|\mu_1, \sigma_1, \mu_2, \sigma_2, \rho)}{\partial F_{\text{NB}}(y_1; \mu_1, \sigma_1)\partial F_{\text{NB}}(y_2 - 1; \mu_2, \sigma_2)} + \\
+&\frac{F_{\text{NB}}(y_1 - 1; \mu_1, \sigma_1)}{\partial\sigma_1}\frac{\partial^2 g(y_1, y_2|\mu_1, \sigma_1, \mu_2, \sigma_2, \rho)}{\partial F_{\text{NB}}(y_1 - 1; \mu_1, \sigma_1)\partial F_{\text{NB}}(y_2 - 1; \mu_2, \sigma_2)}
\end{aligned}
$$

and these quantities have been calculated previously.

3.4.14 | Mixed derivative with respect to $\boldsymbol{\delta}_{\sigma_2}$ and $\boldsymbol{\delta}_{\mu_1}$

$$
\begin{aligned}
&\frac{\partial^2 l}{\partial\boldsymbol{\delta}_{\sigma_2}\partial\boldsymbol{\delta}_{\mu_1}} = \\
=&\frac{\partial}{\partial\boldsymbol{\delta}_{\sigma_2}}\left(\frac{\partial l}{\partial\boldsymbol{\delta}_{\mu_1}}\right) = \\
=&\frac{\partial}{\partial\boldsymbol{\delta}_{\sigma_2}}\left(\frac{1}{f}\frac{\partial f}{\partial\mu_1}\frac{\partial\mu_1}{\partial\boldsymbol{\delta}_{\mu_1}} - \text{diag}\left\{\boldsymbol{\lambda}_{\mu_1}\right\}\boldsymbol{u}_{\mu_1}\right) = \\
=&\left(\frac{\partial}{\partial\boldsymbol{\delta}_{\sigma_2}}\frac{1}{f}\right)\frac{\partial f}{\partial\,\mu_1}\frac{\partial\mu_1}{\partial\boldsymbol{\delta}_{\mu_1}} + \frac{1}{f}\left(\frac{\partial}{\partial\boldsymbol{\delta}_{\sigma_2}}\frac{\partial f}{\partial\mu_1}\right)\frac{\partial\mu_1}{\partial\boldsymbol{\delta}_{\mu_1}} + \frac{1}{f}\frac{\partial f}{\partial\mu_1}\left(\frac{\partial}{\partial\boldsymbol{\delta}_{\sigma_2}}\frac{\partial\mu_1}{\partial\boldsymbol{\delta}_{\mu_1}}\right)
\end{aligned}
$$

where

$$\frac{\partial}{\partial \boldsymbol{\delta}_{\sigma_2}} \frac{\partial \mu_1}{\partial \boldsymbol{\delta}_{\mu_1}} = 0$$

and

$$\frac{\partial}{\partial \boldsymbol{\delta}_{\sigma_2}} \frac{1}{f} = \frac{-1}{f^2} \left(\frac{\partial f}{\partial \boldsymbol{\delta}_{\sigma_2}} \right)$$

where $\frac{\partial f}{\partial \boldsymbol{\delta}_{\sigma_2}}$ was calculated in 3.3.6, and

$$\begin{aligned}
&\frac{\partial}{\partial \boldsymbol{\delta}_{\sigma_2}} \frac{\partial f}{\partial \mu_1} = \\
&= \frac{\partial \sigma_2}{\partial \boldsymbol{\delta}_{\sigma_2}} \left(\frac{\partial}{\partial \sigma_2} \frac{\partial f}{\partial \mu_1} \right) = \\
&= \frac{\partial \sigma_2}{\partial \boldsymbol{\delta}_{\sigma_2}} \begin{cases} (1-p_1)(1-p_2) \frac{\partial^2}{\partial \sigma_2 \partial \mu_1} g(y_1, y_2 | \mu_1, \sigma_1, \mu_2, \sigma_2, \rho) & \text{if ...} \\ (1-p_1)(1-p_2) \frac{\partial^2}{\partial \sigma_2 \partial \mu_1} g(y_1, y_2 | \mu_1, \sigma_1, \mu_2, \sigma_2, \rho) + (1-p_1) p_2 \frac{\partial^2}{\partial \sigma_2 \partial \mu_1} f_{\text{NB}}(y_1; \mu_1, \sigma_1) & \text{if ...} \\ (1-p_1)(1-p_2) \frac{\partial^2}{\partial \sigma_2 \partial \mu_1} g(y_1, y_2 | \mu_1, \sigma_1, \mu_2, \sigma_2, \rho) & \text{if ...} \\ (1-p_1)(1-p_2) \frac{\partial^2}{\partial \sigma_2 \partial \mu_1} g(y_1, y_2 | \mu_1, \sigma_1, \mu_2, \sigma_2, \rho) + (1-p_1) p_2 \frac{\partial^2}{\partial \sigma_2 \partial \mu_1} f_{\text{NB}}(y_1; \mu_1, \sigma_1) & \text{if ...} \end{cases}
\end{aligned}$$

where

$$\frac{\partial^2}{\partial \sigma_2 \partial \mu_1} f_{\text{NB}}(y_1; \mu_1, \sigma_1) = 0$$

and

$$\begin{aligned}
&\frac{\partial^2}{\partial \sigma_2 \partial \mu_1} g(y_1, y_2 | \mu_1, \sigma_1, \mu_2, \sigma_2, \rho) = \\
&= \frac{\partial}{\partial \sigma_2} \left(\frac{\partial}{\partial \mu_1} g(y_1, y_2 | \mu_1, \sigma_1, \mu_2, \sigma_2, \rho) \right) = \\
&= \frac{\partial}{\partial \sigma_2} \left(\frac{\partial g(y_1, y_2 | \mu_1, \sigma_1, \mu_2, \sigma_2, \rho)}{F_{\text{NB}}(y_1; \mu_1, \sigma_1)} \frac{\partial F_{\text{NB}}(y_1; \mu_1, \sigma_1)}{\partial \mu_1} + \right. \\
&\left. + \frac{\partial g(y_1, y_2 | \mu_1, \sigma_1, \mu_2, \sigma_2, \rho)}{\partial F_{\text{NB}}(y_1 - 1; \mu_1, \sigma_1)} \frac{\partial F_{\text{NB}}(y_1 - 1; \mu_1, \sigma_1)}{\partial \mu_1} \right) = \\
&= \left(\frac{\partial}{\partial \sigma_2} \frac{\partial g(y_1, y_2 | \mu_1, \sigma_1, \mu_2, \sigma_2, \rho)}{F_{\text{NB}}(y_1; \mu_1, \sigma_1)} \right) \frac{\partial F_{\text{NB}}(y_1; \mu_1, \sigma_1)}{\partial \mu_1} + \\
&+ \frac{\partial g(y_1, y_2 | \mu_1, \sigma_1, \mu_2, \sigma_2, \rho)}{F_{\text{NB}}(y_1; \mu_1, \sigma_1)} \left(\frac{\partial}{\partial \sigma_2} \frac{\partial F_{\text{NB}}(y_1; \mu_1, \sigma_1)}{\partial \mu_1} \right) + \\
&+ \left(\frac{\partial}{\partial \sigma_2} \frac{\partial g(y_1, y_2 | \mu_1, \sigma_1, \mu_2, \sigma_2, \rho)}{F_{\text{NB}}(y_1 - 1; \mu_1, \sigma_1)} \right) \frac{\partial F_{\text{NB}}(y_1 - 1; \mu_1, \sigma_1)}{\partial \mu_1} + \\
&+ \frac{\partial g(y_1, y_2 | \mu_1, \sigma_1, \mu_2, \sigma_2, \rho)}{F_{\text{NB}}(y_1 - 1; \mu_1, \sigma_1)} \left(\frac{\partial}{\partial \sigma_2} \frac{\partial F_{\text{NB}}(y_1 - 1; \mu_1, \sigma_1)}{\partial \mu_1} \right) = \\
&= \left(\frac{\partial}{\partial \sigma_2} \frac{\partial g(y_1, y_2 | \mu_1, \sigma_1, \mu_2, \sigma_2, \rho)}{F_{\text{NB}}(y_1; \mu_1, \sigma_1)} \right) \frac{\partial F_{\text{NB}}(y_1; \mu_1, \sigma_1)}{\partial \mu_1} + \\
&+ \left(\frac{\partial}{\partial \sigma_2} \frac{\partial g(y_1, y_2 | \mu_1, \sigma_1, \mu_2, \sigma_2, \rho)}{F_{\text{NB}}(y_1 - 1; \mu_1, \sigma_1)} \right) \frac{\partial F_{\text{NB}}(y_1 - 1; \mu_1, \sigma_1)}{\partial \mu_1}
\end{aligned}$$

where

$$\begin{aligned}
&\frac{\partial}{\partial \sigma_2} \frac{\partial g(y_1, y_2 | \mu_1, \sigma_1, \mu_2, \sigma_2, \rho)}{F_{\text{NB}}(y_1; \mu_1, \sigma_1)} = \\
&= \frac{F_{\text{NB}}(y_2; \mu_2, \sigma_2)}{\partial \sigma_2} \frac{\partial^2 g(y_1, y_2 | \mu_1, \sigma_1, \mu_2, \sigma_2, \rho)}{F_{\text{NB}}(y_2; \mu_2, \sigma_2) F_{\text{NB}}(y_1; \mu_1, \sigma_1)} + \\
&+ \frac{F_{\text{NB}}(y_2 - 1; \mu_2, \sigma_2)}{\partial \sigma_2} \frac{\partial^2 g(y_1, y_2 | \mu_1, \sigma_1, \mu_2, \sigma_2, \rho)}{F_{\text{NB}}(y_2 - 1; \mu_2, \sigma_2) F_{\text{NB}}(y_1; \mu_1, \sigma_1)}
\end{aligned}$$

and

$$\frac{\partial}{\partial\sigma_2}\frac{\partial g(y_1,y_2|\mu_1,\sigma_1,\mu_2,\sigma_2,\rho)}{F_{\mathrm{NB}}(y_1-1;\mu_1,\sigma_1)}=$$
$$=\frac{F_{\mathrm{NB}}(y_2;\mu_2,\sigma_2)}{\partial\sigma_2}\frac{\partial^2 g(y_1,y_2|\mu_1,\sigma_1,\mu_2,\sigma_2,\rho)}{\partial F_{\mathrm{NB}}(y_2;\mu_2,\sigma_2)\partial F_{\mathrm{NB}}(y_1-1;\mu_1,\sigma_1)}+$$
$$+\frac{F_{\mathrm{NB}}(y_2-1;\mu_2,\sigma_2)}{\partial\sigma_2}\frac{\partial^2 g(y_1,y_2|\mu_1,\sigma_1,\mu_2,\sigma_2,\rho)}{\partial F_{\mathrm{NB}}(y_2-1;\mu_2,\sigma_2)\partial F_{\mathrm{NB}}(y_1-1;\mu_1,\sigma_1)}$$

and these quantities have been calculated previously.

3.4.15 | Second derivative with respect to $\boldsymbol{\delta}_\rho$

$$\frac{\partial^2 l}{\partial\boldsymbol{\delta}_\rho^2}=$$
$$=\frac{\partial}{\partial\boldsymbol{\delta}_\rho}\left(\frac{\partial l}{\partial\boldsymbol{\delta}_\rho}\right)=$$
$$=\frac{\partial}{\partial\boldsymbol{\delta}_\rho}\left(\frac{1}{f}\frac{\partial f}{\partial\rho}\frac{\partial\rho}{\partial\boldsymbol{\delta}_\rho}-\mathrm{diag}\left\{\boldsymbol{\lambda}_\rho\right\}\boldsymbol{u}_\rho\right)=$$
$$=\left(\frac{\partial}{\partial\boldsymbol{\delta}_\rho}\frac{1}{f}\right)\frac{\partial f}{\partial\rho}\frac{\partial\rho}{\partial\boldsymbol{\delta}_\rho}+\frac{1}{f}\left(\frac{\partial}{\partial\boldsymbol{\delta}_\rho}\frac{\partial f}{\partial\rho}\right)\frac{\partial\rho}{\partial\boldsymbol{\delta}_\rho}+\frac{1}{f}\frac{\partial f}{\partial\rho}\left(\frac{\partial}{\partial\boldsymbol{\delta}_\rho}\frac{\partial\rho}{\partial\boldsymbol{\delta}_\rho}\right)-\mathrm{diag}\left\{\boldsymbol{\lambda}_\rho\right\}$$

where

$$\frac{\partial}{\partial\boldsymbol{\delta}_\rho}\frac{1}{f}=\frac{-1}{f^2}\left(\frac{\partial f}{\partial\boldsymbol{\delta}_\rho}\right)$$

where $\frac{\partial f}{\partial\boldsymbol{\delta}_\rho}$ was calculated in 3.3.7, and

$$\frac{\partial}{\partial\boldsymbol{\delta}_\rho}\left(\frac{\partial\rho}{\partial\boldsymbol{\delta}_\rho}\right)=$$
$$=\frac{\partial}{\partial\boldsymbol{\delta}_\rho}\left(\cosh^2\left\{\boldsymbol{\delta}_\rho^\top\boldsymbol{x}_\rho\right\}\boldsymbol{x}_\rho\right)=$$
$$=-2\mathrm{sech}^2\left\{\boldsymbol{\delta}_\rho^\top\boldsymbol{x}_\rho\right\}\tanh\left\{\boldsymbol{\delta}_\rho^\top\boldsymbol{x}_\rho\right\}\boldsymbol{x}_\rho\boldsymbol{x}_\rho^\top$$

and

$$\frac{\partial}{\partial\boldsymbol{\delta}_\rho}\frac{\partial f}{\partial\rho}=$$
$$=\frac{\partial\rho}{\partial\boldsymbol{\delta}_\rho}\left(\frac{\partial}{\partial\rho}\frac{\partial f}{\partial\rho}\right)=$$
$$=\frac{\partial\rho}{\partial\boldsymbol{\delta}_\rho}\left((1-p_1)(1-p_2)\frac{\partial^2}{\partial\rho^2}g(y_1,y_2|\mu_1,\sigma_1,\mu_2,\sigma_2,\rho)\right)$$

where

$$\frac{\partial^2}{\partial\rho^2}g(y_1,y_2|\mu_1,\sigma_1,\mu_2,\sigma_2,\rho)=$$
$$=\frac{\partial}{\partial\rho}\left(\frac{\partial}{\partial\rho}g(y_1,y_2|\mu_1,\sigma_1,\mu_2,\sigma_2,\rho)\right)=$$
$$=\frac{\partial}{\partial\rho}\int_{\Phi^{-1}(F_{\mathrm{NB}}(y_1-1;\mu_1,\sigma_1))}^{\Phi^{-1}(F_{\mathrm{NB}}(y_1;\mu_1,\sigma_1))}\int_{\Phi^{-1}(F_{\mathrm{NB}}(y_2-1;\mu_2,\sigma_2))}^{\Phi^{-1}(F_{\mathrm{NB}}(y_2;\mu_2,\sigma_2))}\left[\frac{\rho}{1-[\rho]^2}+\frac{(\rho t_1-t_2)(\rho t_2-t_1)}{(1-[\rho]^2)^2}\right]\phi_2(t_1,t_2|\rho)dt_2dt_1=$$
$$=\int_{\Phi^{-1}(F_{\mathrm{NB}}(y_1-1;\mu_1,\sigma_1))}^{\Phi^{-1}(F_{\mathrm{NB}}(y_1;\mu_1,\sigma_1))}\int_{\Phi^{-1}(F_{\mathrm{NB}}(y_2-1;\mu_2,\sigma_2))}^{\Phi^{-1}(F_{\mathrm{NB}}(y_2;\mu_2,\sigma_2))}\frac{\partial}{\partial\rho}\left[\frac{\rho}{1-[\rho]^2}+\frac{(\rho t_1-t_2)(\rho t_2-t_1)}{(1-[\rho]^2)^2}\right]\times$$
$$\times\frac{1}{2\pi\sqrt{1-[\rho]^2}}\exp\left\{\frac{-\left(t_1^2+t_2^2-2[\rho]t_1t_2\right)}{2\left(1-[\rho]^2\right)}\right\}dt_2dt_1$$

3.4.16 Mixed derivative with respect to δ_ρ and δ_{μ_1}

$$\begin{aligned}
&\frac{\partial^2 l}{\partial \boldsymbol{\delta}_\rho \partial \boldsymbol{\delta}_{\mu_1}} = \\
&= \frac{\partial}{\partial \boldsymbol{\delta}_\rho}\left(\frac{\partial l}{\partial \boldsymbol{\delta}_{\mu_1}}\right) = \\
&= \frac{\partial}{\partial \boldsymbol{\delta}_\rho}\left(\frac{1}{f}\frac{\partial f}{\partial \mu_1}\frac{\partial \mu_1}{\partial \boldsymbol{\delta}_{\mu_1}} - \operatorname{diag}\left\{\boldsymbol{\lambda}_{\mu_1}\right\}\boldsymbol{u}_{\mu_1}\right) = \\
&= \left(\frac{\partial}{\partial \boldsymbol{\delta}_\rho}\frac{1}{f}\right)\frac{\partial f}{\partial \mu_1}\frac{\partial \mu_1}{\partial \boldsymbol{\delta}_{\mu_1}} + \frac{1}{f}\left(\frac{\partial}{\partial \boldsymbol{\delta}_\rho}\frac{\partial f}{\partial \mu_1}\right)\frac{\partial \mu_1}{\partial \boldsymbol{\delta}_{\mu_1}} + \frac{1}{f}\frac{\partial f}{\partial \mu_1}\left(\frac{\partial}{\partial \boldsymbol{\delta}_\rho}\frac{\partial \mu_1}{\partial \boldsymbol{\delta}_{\mu_1}}\right)
\end{aligned}$$

where

$$\frac{\partial}{\partial \boldsymbol{\delta}_\rho}\frac{\partial \mu_1}{\partial \boldsymbol{\delta}_{\mu_1}} = 0$$

and

$$\frac{\partial}{\partial \boldsymbol{\delta}_\rho}\frac{1}{f} = \frac{-1}{f^2}\left(\frac{\partial f}{\partial \boldsymbol{\delta}_\rho}\right)$$

where $\frac{\partial f}{\partial \boldsymbol{\delta}_\rho}$ was calculated in 3.3.7, and

$$\begin{aligned}
&\frac{\partial}{\partial \boldsymbol{\delta}_\rho}\frac{\partial f}{\partial \mu_1} = \frac{\partial \rho}{\partial \boldsymbol{\delta}_\rho}\left(\frac{\partial}{\partial \rho}\frac{\partial f}{\partial \mu_1}\right) = \\
&= \frac{\partial \rho}{\partial \boldsymbol{\delta}_\rho}\begin{cases}
(1-p_1)(1-p_2)\frac{\partial^2}{\partial \rho \partial \mu_1} g(y_1, y_2 | \mu_1, \sigma_1, \mu_2, \sigma_2, \rho) & \text{if ...}\\
(1-p_1)(1-p_2)\frac{\partial^2}{\partial \rho \partial \mu_1} g(y_1, y_2 | \mu_1, \sigma_1, \mu_2, \sigma_2, \rho) + (1-p_1)p_2\frac{\partial^2}{\partial \rho \partial \mu_1} f_{\mathrm{NB}}(y_1; \mu_1, \sigma_1) & \text{if ...}\\
(1-p_1)(1-p_2)\frac{\partial^2}{\partial \rho \partial \mu_1} g(y_1, y_2 | \mu_1, \sigma_1, \mu_2, \sigma_2, \rho) & \text{if ...}\\
(1-p_1)(1-p_2)\frac{\partial^2}{\partial \rho \partial \mu_1} g(y_1, y_2 | \mu_1, \sigma_1, \mu_2, \sigma_2, \rho) + (1-p_1)p_2\frac{\partial^2}{\partial \rho \partial \mu_1} f_{\mathrm{NB}}(y_1; \mu_1, \sigma_1) & \text{if ...}
\end{cases}
\end{aligned}$$

where $\frac{\partial^2}{\partial \rho \partial \mu_1} f_{\mathrm{NB}}(y_1; \mu_1, \sigma_1) = 0$ and

$$\begin{aligned}
&\frac{\partial^2}{\partial \rho \partial \mu_1} g(y_1, y_2 | \mu_1, \sigma_1, \mu_2, \sigma_2, \rho) = \\
&= \frac{\partial}{\partial \rho}\left(\frac{\partial}{\partial \mu_1} g(y_1, y_2 | \mu_1, \sigma_1, \mu_2, \sigma_2, \rho)\right) = \\
&= \frac{\partial}{\partial \rho}\left(\frac{\partial g(y_1, y_2 | \mu_1, \sigma_1, \mu_2, \sigma_2, \rho)}{F_{\mathrm{NB}}(y_1; \mu_1, \sigma_1)}\frac{\partial F_{\mathrm{NB}}(y_1; \mu_1, \sigma_1)}{\partial \mu_1} + \frac{\partial g(y_1, y_2 | \mu_1, \sigma_1, \mu_2, \sigma_2, \rho)}{\partial F_{\mathrm{NB}}(y_1 - 1; \mu_1, \sigma_1)}\frac{\partial F_{\mathrm{NB}}(y_1 - 1; \mu_1, \sigma_1)}{\partial \mu_1}\right) = \\
&= \left(\frac{\partial}{\partial \rho}\frac{\partial g(y_1, y_2 | \mu_1, \sigma_1, \mu_2, \sigma_2, \rho)}{F_{\mathrm{NB}}(y_1; \mu_1, \sigma_1)}\right)\frac{\partial F_{\mathrm{NB}}(y_1; \mu_1, \sigma_1)}{\partial \mu_1} + \\
&+ \frac{\partial g(y_1, y_2 | \mu_1, \sigma_1, \mu_2, \sigma_2, \rho)}{F_{\mathrm{NB}}(y_1; \mu_1, \sigma_1)}\left(\frac{\partial}{\partial \rho}\frac{\partial F_{\mathrm{NB}}(y_1; \mu_1, \sigma_1)}{\partial \mu_1}\right) + \\
&+ \left(\frac{\partial}{\partial \rho}\frac{\partial g(y_1, y_2 | \mu_1, \sigma_1, \mu_2, \sigma_2, \rho)}{F_{\mathrm{NB}}(y_1 - 1; \mu_1, \sigma_1)}\right)\frac{\partial F_{\mathrm{NB}}(y_1 - 1; \mu_1, \sigma_1)}{\partial \mu_1} + \\
&+ \frac{\partial g(y_1, y_2 | \mu_1, \sigma_1, \mu_2, \sigma_2, \rho)}{F_{\mathrm{NB}}(y_1 - 1; \mu_1, \sigma_1)}\left(\frac{\partial}{\partial \rho}\frac{\partial F_{\mathrm{NB}}(y_1 - 1; \mu_1, \sigma_1)}{\partial \mu_1}\right) = \\
&= \left(\frac{\partial}{\partial \rho}\frac{\partial g(y_1, y_2 | \mu_1, \sigma_1, \mu_2, \sigma_2, \rho)}{F_{\mathrm{NB}}(y_1; \mu_1, \sigma_1)}\right)\frac{\partial F_{\mathrm{NB}}(y_1; \mu_1, \sigma_1)}{\partial \mu_1} + \\
&+ \left(\frac{\partial}{\partial \rho}\frac{\partial g(y_1, y_2 | \mu_1, \sigma_1, \mu_2, \sigma_2, \rho)}{F_{\mathrm{NB}}(y_1 - 1; \mu_1, \sigma_1)}\right)\frac{\partial F_{\mathrm{NB}}(y_1 - 1; \mu_1, \sigma_1)}{\partial \mu_1}
\end{aligned}$$

where

$$\frac{\partial}{\partial\rho}\frac{\partial g(y_1,y_2|\mu_1,\sigma_1,\mu_2,\sigma_2,\rho)}{F_{\text{NB}}(y_1;\mu_1,\sigma_1)}=$$
$$=\frac{\partial}{\partial\rho}\left[\Phi\left(\frac{\Phi^{-1}\left\{F_{\text{NB}}(y_2;\mu_2,\sigma_2)\right\}-\Phi^{-1}\left\{F_{\text{NB}}(y_1;\mu_1,\sigma_1)\right\}\rho}{\sqrt{1-\rho^2}}\right)+\right.$$
$$\left.-\Phi\left(\frac{\Phi^{-1}\left\{F_{\text{NB}}(y_2-1;\mu_2,\sigma_2)\right\}-\Phi^{-1}\left\{F_{\text{NB}}(y_1;\mu_1,\sigma_1)\right\}\rho}{\sqrt{1-\rho^2}}\right)\right]=$$
$$=\frac{1}{2\pi}\int_{\Phi^{-1}(F_{\text{NB}}(y_2-1;\mu_2,\sigma_2))}^{\Phi^{-1}(F_{\text{NB}}(y_2;\mu_2,\sigma_2))}\left[\frac{\rho}{1-[\rho]^2}+\frac{(\rho\left[\Phi^{-1}(F_{\text{NB}}(y_1;\mu_1,\sigma_1))\right]-t_2)(\rho t_2-\left[\Phi^{-1}(F_{\text{NB}}(y_1;\mu_1,\sigma_1))\right])}{(1-[\rho]^2)^2}\right]$$
$$\times\,\text{dnorm}\left(t_2,\ \text{mean}=\rho\Phi^{-1}(F_{\text{NB}}(y_1;\mu_1,\sigma_1)),\text{sd}=\sqrt{1-[\rho]^2}\right)\,dt_2$$

and

$$\frac{\partial}{\partial\rho}\frac{\partial g(y_1,y_2|\mu_1,\sigma_1,\mu_2,\sigma_2,\rho)}{F_{\text{NB}}(y_1-1;\mu_1,\sigma_1)}=$$
$$=\frac{\partial}{\partial\rho}\left[-\Phi\left(\frac{\Phi^{-1}\left\{F_{\text{NB}}(y_2;\mu_2,\sigma_2)\right\}-\Phi^{-1}\left\{F_{\text{NB}}(y_1-1;\mu_1,\sigma_1)\right\}\rho}{\sqrt{1-\rho^2}}\right)+\right.$$
$$\left.+\Phi\left(\frac{\Phi^{-1}\left\{F_{\text{NB}}(y_2-1;\mu_2,\sigma_2)\right\}-\Phi^{-1}\left\{F_{\text{NB}}(y_1-1;\mu_1,\sigma_1)\right\}\rho}{\sqrt{1-\rho^2}}\right)\right]=$$
$$=\int_{\Phi^{-1}(F_{\text{NB}}(y_1-1;\mu_1,\sigma_1))}^{\Phi^{-1}(F_{\text{NB}}(y_1;\mu_1,\sigma_1))}\left[\frac{\rho}{1-[\rho]^2}+\frac{(\rho\left[\Phi^{-1}(F_{\text{NB}}(y_2-1;\mu_2,\sigma_2))\right]-t_2)(\rho t_2-\left[\Phi^{-1}(F_{\text{NB}}(y_2-1;\mu_2,\sigma_2))\right])}{(1-[\rho]^2)^2}\right]$$
$$\times\frac{-1}{2\pi}\text{dnorm}\left(t_2,\ \text{mean}=\rho\Phi^{-1}(F_{\text{NB}}(y_2-1;\mu_2,\sigma_2)),\text{sd}=\sqrt{1-[\rho]^2}\right)\,dt_2$$

3.4.17 | Mixed derivative with respect to $\boldsymbol{\delta}_\rho$ and $\boldsymbol{\delta}_{\sigma_1}$

$$\frac{\partial^2 l}{\partial\boldsymbol{\delta}_\rho\partial\boldsymbol{\delta}_{\sigma_1}}=$$
$$=\frac{\partial}{\partial\boldsymbol{\delta}_\rho}\left(\frac{\partial l}{\partial\boldsymbol{\delta}_{\sigma_1}}\right)=$$
$$=\frac{\partial}{\partial\boldsymbol{\delta}_\rho}\left(\frac{1}{f}\frac{\partial f}{\partial\sigma_1}\frac{\partial\sigma_1}{\partial\boldsymbol{\delta}_{\sigma_1}}-\text{diag}\left\{\boldsymbol{\lambda}_{\sigma_1}\right\}\boldsymbol{u}_{\sigma_1}\right)=$$
$$=\left(\frac{\partial}{\partial\boldsymbol{\delta}_\rho}\frac{1}{f}\right)\frac{\partial f}{\partial\,\sigma_1}\frac{\partial\sigma_1}{\partial\boldsymbol{\delta}_{\sigma_1}}+\frac{1}{f}\left(\frac{\partial}{\partial\boldsymbol{\delta}_\rho}\frac{\partial f}{\partial\sigma_1}\right)\frac{\partial\sigma_1}{\partial\boldsymbol{\delta}_{\sigma_1}}+\frac{1}{f}\frac{\partial f}{\partial\sigma_1}\left(\frac{\partial}{\partial\boldsymbol{\delta}_\rho}\frac{\partial\sigma_1}{\partial\boldsymbol{\delta}_{\sigma_1}}\right)$$

where

$$\frac{\partial}{\partial\boldsymbol{\delta}_\rho}\frac{\partial\sigma_1}{\partial\boldsymbol{\delta}_{\sigma_1}}=0$$

and

$$\frac{\partial}{\partial\boldsymbol{\delta}_\rho}\frac{1}{f}=\frac{-1}{f^2}\left(\frac{\partial f}{\partial\boldsymbol{\delta}_\rho}\right)$$

where $\frac{\partial f}{\partial\boldsymbol{\delta}_\rho}$ was calculated in 3.3.7, and

$$\frac{\partial}{\partial\boldsymbol{\delta}_\rho}\frac{\partial f}{\partial\sigma_1}=\frac{\partial\rho}{\partial\boldsymbol{\delta}_\rho}\left(\frac{\partial}{\partial\rho}\frac{\partial f}{\partial\sigma_1}\right)=$$
$$=\frac{\partial\rho}{\partial\boldsymbol{\delta}_\rho}\begin{cases}(1-p_1)(1-p_2)\frac{\partial^2}{\partial\rho\partial\sigma_1}g(y_1,y_2|\mu_1,\sigma_1,\mu_2,\sigma_2,\rho) & \text{if ...}\\(1-p_1)(1-p_2)\frac{\partial^2}{\partial\rho\partial\sigma_1}g(y_1,y_2|\mu_1,\sigma_1,\mu_2,\sigma_2,\rho)+(1-p_1)p_2\frac{\partial^2}{\partial\rho\partial\sigma_1}f_{\text{NB}}(y_1;\mu_1,\sigma_1) & \text{if ...}\\(1-p_1)(1-p_2)\frac{\partial^2}{\partial\rho\partial\sigma_1}g(y_1,y_2|\mu_1,\sigma_1,\mu_2,\sigma_2,\rho) & \text{if ...}\\(1-p_1)(1-p_2)\frac{\partial^2}{\partial\rho\partial\sigma_1}g(y_1,y_2|\mu_1,\sigma_1,\mu_2,\sigma_2,\rho)+(1-p_1)p_2\frac{\partial^2}{\partial\rho\partial\sigma_1}f_{\text{NB}}(y_1;\mu_1,\sigma_1) & \text{if ...}\end{cases}$$

where $\frac{\partial^2}{\partial\rho\partial\sigma_1} f_{\mathrm{NB}}(y_1;\mu_1,\sigma_1) = 0$ and

$$
\begin{aligned}
&\frac{\partial^2}{\partial\rho\partial\sigma_1} g(y_1, y_2|\mu_1,\sigma_1,\mu_2,\sigma_2,\rho) = \\
=&\frac{\partial}{\partial\rho}\left(\frac{\partial}{\partial\sigma_1} g(y_1, y_2|\mu_1,\sigma_1,\mu_2,\sigma_2,\rho)\right) = \\
=&\frac{\partial}{\partial\rho}\left(\frac{\partial g(y_1, y_2|\mu_1,\sigma_1,\mu_2,\sigma_2,\rho)}{F_{\mathrm{NB}}(y_1;\mu_1,\sigma_1)}\frac{\partial F_{\mathrm{NB}}(y_1;\mu_1,\sigma_1)}{\partial\sigma_1} + \frac{\partial g(y_1, y_2|\mu_1,\sigma_1,\mu_2,\sigma_2,\rho)}{\partial F_{\mathrm{NB}}(y_1-1;\mu_1,\sigma_1)}\frac{\partial F_{\mathrm{NB}}(y_1-1;\mu_1,\sigma_1)}{\partial\sigma_1}\right) = \\
=&\left(\frac{\partial}{\partial\rho}\frac{\partial g(y_1, y_2|\mu_1,\sigma_1,\mu_2,\sigma_2,\rho)}{F_{\mathrm{NB}}(y_1;\mu_1,\sigma_1)}\right)\frac{\partial F_{\mathrm{NB}}(y_1;\mu_1,\sigma_1)}{\partial\sigma_1} + \\
+&\frac{\partial g(y_1, y_2|\mu_1,\sigma_1,\mu_2,\sigma_2,\rho)}{F_{\mathrm{NB}}(y_1;\mu_1,\sigma_1)}\left(\frac{\partial}{\partial\rho}\frac{\partial F_{\mathrm{NB}}(y_1;\mu_1,\sigma_1)}{\partial\sigma_1}\right) + \\
+&\left(\frac{\partial}{\partial\rho}\frac{\partial g(y_1, y_2|\mu_1,\sigma_1,\mu_2,\sigma_2,\rho)}{F_{\mathrm{NB}}(y_1-1;\mu_1,\sigma_1)}\right)\frac{\partial F_{\mathrm{NB}}(y_1-1;\mu_1,\sigma_1)}{\partial\sigma_1} + \\
+&\frac{\partial g(y_1, y_2|\mu_1,\sigma_1,\mu_2,\sigma_2,\rho)}{F_{\mathrm{NB}}(y_1-1;\mu_1,\sigma_1)}\left(\frac{\partial}{\partial\rho}\frac{\partial F_{\mathrm{NB}}(y_1-1;\mu_1,\sigma_1)}{\partial\sigma_1}\right) = \\
=&\left(\frac{\partial}{\partial\rho}\frac{\partial g(y_1, y_2|\mu_1,\sigma_1,\mu_2,\sigma_2,\rho)}{F_{\mathrm{NB}}(y_1;\mu_1,\sigma_1)}\right)\frac{\partial F_{\mathrm{NB}}(y_1;\mu_1,\sigma_1)}{\partial\sigma_1} + \\
+&\left(\frac{\partial}{\partial\rho}\frac{\partial g(y_1, y_2|\mu_1,\sigma_1,\mu_2,\sigma_2,\rho)}{F_{\mathrm{NB}}(y_1-1;\mu_1,\sigma_1)}\right)\frac{\partial F_{\mathrm{NB}}(y_1-1;\mu_1,\sigma_1)}{\partial\sigma_1}
\end{aligned}
$$

where $\frac{\partial F_{\mathrm{NB}}(y_1;\mu_1,\sigma_1)}{\partial\sigma_1}$ and $\frac{\partial F_{\mathrm{NB}}(y_1-1;\mu_1,\sigma_1)}{\partial\sigma_1}$ were calculated in 3.4.16.

REFERENCES

1. Kullback S, Leibler RA. On Information and Sufficiency. *The Annals of Mathematical Statistics.* 1951;22(1):7986. doi: 10.1214/aoms/1177729694
2. Nelsen RB. *An Introduction to Copulas*. Springer Series in StatisticsNew York, NY: Springer. 2 ed., 2006.
3. Sklar MJ. Fonctions de repartition a n dimensions et leurs marges. 1959.
4. Demarta S, McNeil AJ. The t Copula and Related Copulas. *International Statistical Review.* 2007;73(1):111129. doi: 10.1111/j.1751-5823.2005.tb00254.x
5. Ma Z, Hanson TE, Ho YY. Flexible bivariate correlated count data regression. *Statistics in Medicine.* 2020;39(25):34763490. doi: 10.1002/sim.8676
6. Ma Z, Davis SW, Ho YY. Flexible Copula Model for Integrating Correlated Multi-Omics Data from Single-Cell Experiments. *Biometrics.* 2022;79(2):15591572. doi: 10.1111/biom.13701
7. Wu N, Yin F, Ou-Yang L, Zhu Z, Xie W. Joint learning of multiple gene networks from single-cell gene expression data. *Computational and Structural Biotechnology Journal.* 2020;18:25832595. doi: 10.1016/j.csbj.2020.09.004
8. Sun T, Song D, Li WV, Li JJ. scDesign2: a transparent simulator that generates high-fidelity single-cell gene expression count data with gene correlations captured. *Genome Biology.* 2021;22(1). doi: 10.1186/s13059-021-02367-2
9. Meyer C. The Bivariate Normal Copula. *Communications in Statistics - Theory and Methods.* 2013;42(13):24022422. doi: 10.1080/03610926.2011.611316

List of Figures

S1 Plate scenario: Power of scCOSMiX to detect differential co-expression in datasets with 5, 10, 15 patients. Data is simulated using parameter values specified in Equation (6) in the main manuscript based on the plate-based dataset GSE108989. The null hypothesis being tested is that there is no differential co-expression ($H_0 : \tau_1 = 0$). The number of patients is set to $m \in \{5, 10, 15\}$, τ_0 is set to 0, and τ_1 is varied between 0 and 0.4. For each combination of τ_1 and m, $B = 500$ datasets are simulated, and power is calculated as the proportion of times in which the null hypothesis is rejected. . . . 30

S2 Plate scenario: Q-Q plots for the distribution of p-values for differential co-expression, based on datasets generated using scDesign3 with the plate-based dataset GSE108989 as a template. For each of the $B = 100$ simulated replicates, 1,225 gene pairs are simulated under the null hypothesis. Each method is then applied to each replicate dataset, and p-values are computed for all 1,225 gene pairs. The black curves show the empirical distribution of p-values, averaged across replicates for each method. The dashed line represents the expected Unif(0, 1) distribution under the null. . . . 31

S3 Plate scenario: Precision-recall curves for differential co-expression, evaluated on data simulated based on datasets generated using scDesign3 with the plate-based dataset GSE108989 as a template. $B = 100$ datasets were simulated using scDesign3 based on a subset of 1,225 gene pairs from the droplet template dataset. 140 of the gene pairs were randomly selected and manually given a differential co-expression of 0.20 while the remaining 1,085 gene pairs have no differential co-expression. . . . 32

S4 Plate scenario: Heatmaps of gene-gene correlation estimates for selected genes in CD4 and CD8 cells, with an additional heatmap showing co-expression changes between the two lineages. . . . 33

S5 Comparison of $\log(f(y_1, y_2 ; \boldsymbol{\theta}))$ and $\log(f^*(y_1, y_2 ; \boldsymbol{\theta}^*))$ for $\boldsymbol{\theta}$ and $\boldsymbol{\theta}^*$ from Table S6. Plotting domain of y_1, y_2 has been truncated for visual clarity. Note that in this low over-dispersion case, the non-zero-inflated model cannot capture the shape of the true distribution. . . . 34

S6 Comparison of $\log(f(y_1, y_2 ; \boldsymbol{\theta}))$ and $\log(f^*(y_1, y_2 ; \boldsymbol{\theta}^*))$ for $\boldsymbol{\theta}$ and $\boldsymbol{\theta}^*$ from Table S7. Plotting domain of y_1, y_2 has been truncated for visual clarity. Note that in this high over-dispersion case, the non-zero-inflated model is better able to capture the shape of the true distribution. . . . 35

S7 KL divergence between scCOSMiX model and the non-zero-inflated version from Equation 1 in Section 2.2 of the main manuscript, computed across a grid of (σ, μ) values. For each (σ, μ) pair on the grid, we set the truth to $\boldsymbol{\theta} = (\mu, \mu, \sigma, \sigma, 0.5, 0.3, 0.3)$ and we compute the KL divergence. This plot demonstrates that the trend observed in Figures S5 and S6, of larger σ corresponding to smaller KL divergence, extends across the range of μ and σ. 36

S8 Bias of the pseudotrue parameter ρ^* across a grid of (σ, μ) values. The ρ^* corresponding to each (σ, μ) is obtained during the KL divergence computations from Figure S7– where the truth was set to $\boldsymbol{\theta} = (\mu, \mu, \sigma, \sigma, 0.5, 0.3, 0.3)$. We observe a similar trend as was found in Tables S6 and S7: that larger σ corresponds closer proximity of ρ^* to true ρ. Meaning for true distributions with more over-dispersion, the non-zero-inflated model does not attenuate the dependence parameter as drastically as it does for distributions with less over-dispersion. . . . 37

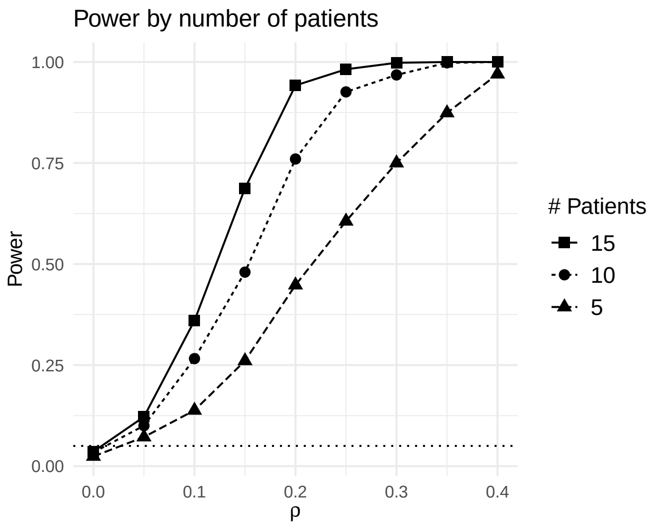

FIGURE S1 Plate scenario: Power of scCOSMiX to detect differential co-expression in datasets with $5, 10, 15$ patients. Data is simulated using parameter values specified in Equation (6) in the main manuscript based on the plate-based dataset GSE108989. The null hypothesis being tested is that there is no differential co-expression ($H_0 : \tau_1 = 0$). The number of patients is set to $m \in \{5, 10, 15\}$, τ_0 is set to 0, and τ_1 is varied between 0 and 0.4. For each combination of τ_1 and m, $B = 500$ datasets are simulated, and power is calculated as the proportion of times in which the null hypothesis is rejected.

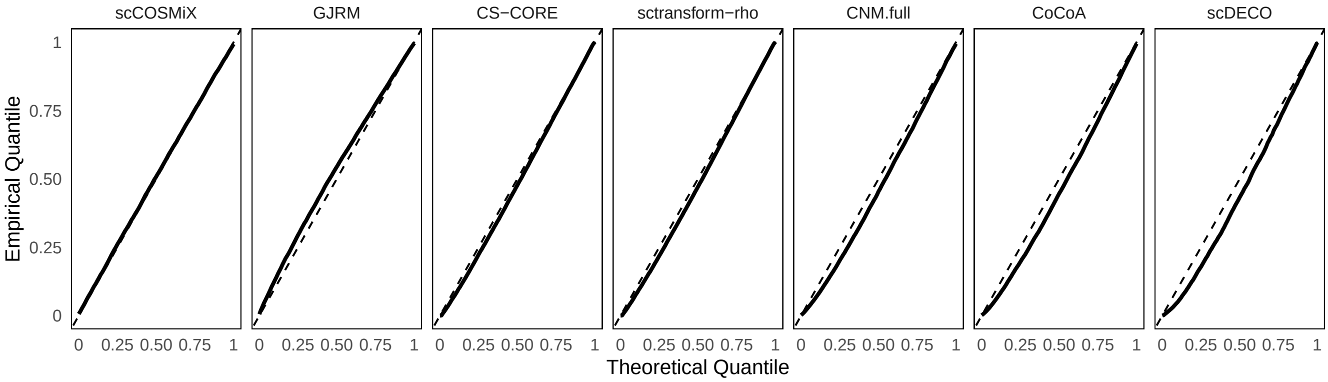

FIGURE S2 Plate scenario: Q-Q plots for the distribution of p-values for differential co-expression, based on datasets generated using scDesign3 with the plate-based dataset GSE108989 as a template. For each of the $B = 100$ simulated replicates, 1,225 gene pairs are simulated under the null hypothesis. Each method is then applied to each replicate dataset, and p-values are computed for all 1,225 gene pairs. The black curves show the empirical distribution of p-values, averaged across replicates for each method. The dashed line represents the expected Unif(0, 1) distribution under the null.

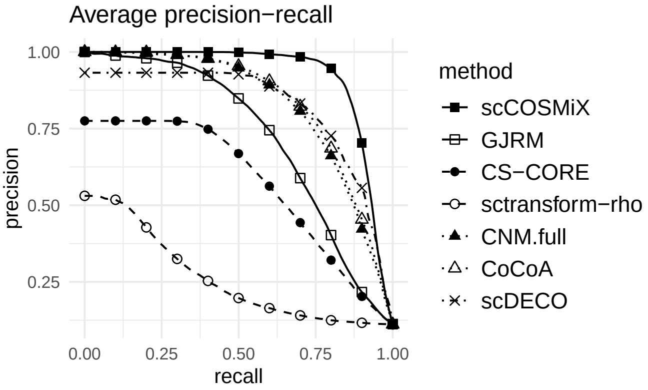

FIGURE S3 Plate scenario: Precision-recall curves for differential co-expression, evaluated on data simulated based on datasets generated using scDesign3 with the plate-based dataset GSE108989 as a template. $B = 100$ datasets were simulated using scDesign3 based on a subset of 1,225 gene pairs from the droplet template dataset. 140 of the gene pairs were randomly selected and manually given a differential co-expression of 0.20 while the remaining 1,085 gene pairs have no differential co-expression.

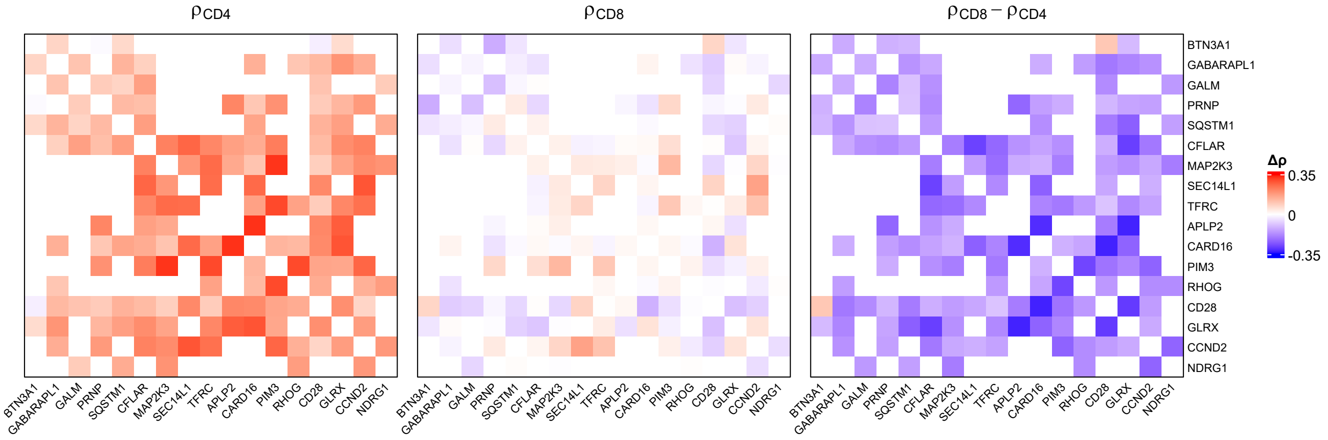

FIGURE S4 Plate scenario: Heatmaps of gene-gene correlation estimates for selected genes in CD4 and CD8 cells, with an additional heatmap showing co-expression changes between the two lineages.

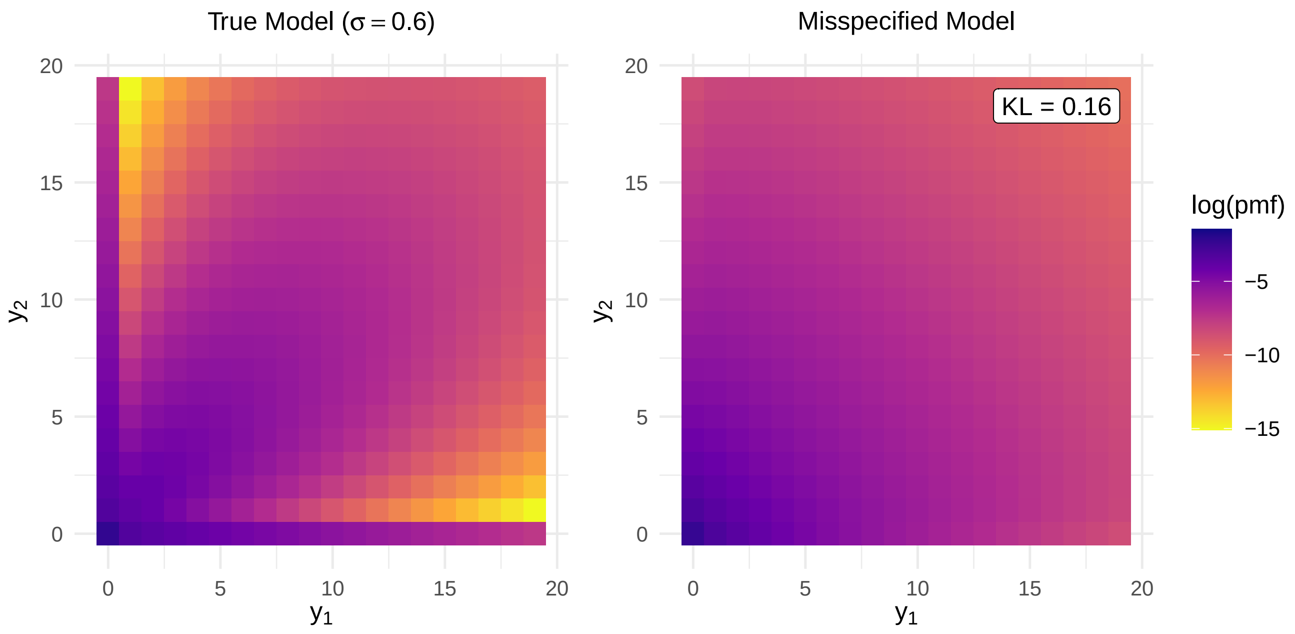

FIGURE S5 Comparison of $\log(f(y_1, y_2 \,;\, \boldsymbol{\theta}))$ and $\log(f^*(y_1, y_2 \,;\boldsymbol{\theta}^*))$ for $\boldsymbol{\theta}$ and $\boldsymbol{\theta}^*$ from Table S6. Plotting domain of y_1, y_2 has been truncated for visual clarity. Note that in this low over-dispersion case, the non-zero-inflated model cannot capture the shape of the true distribution.

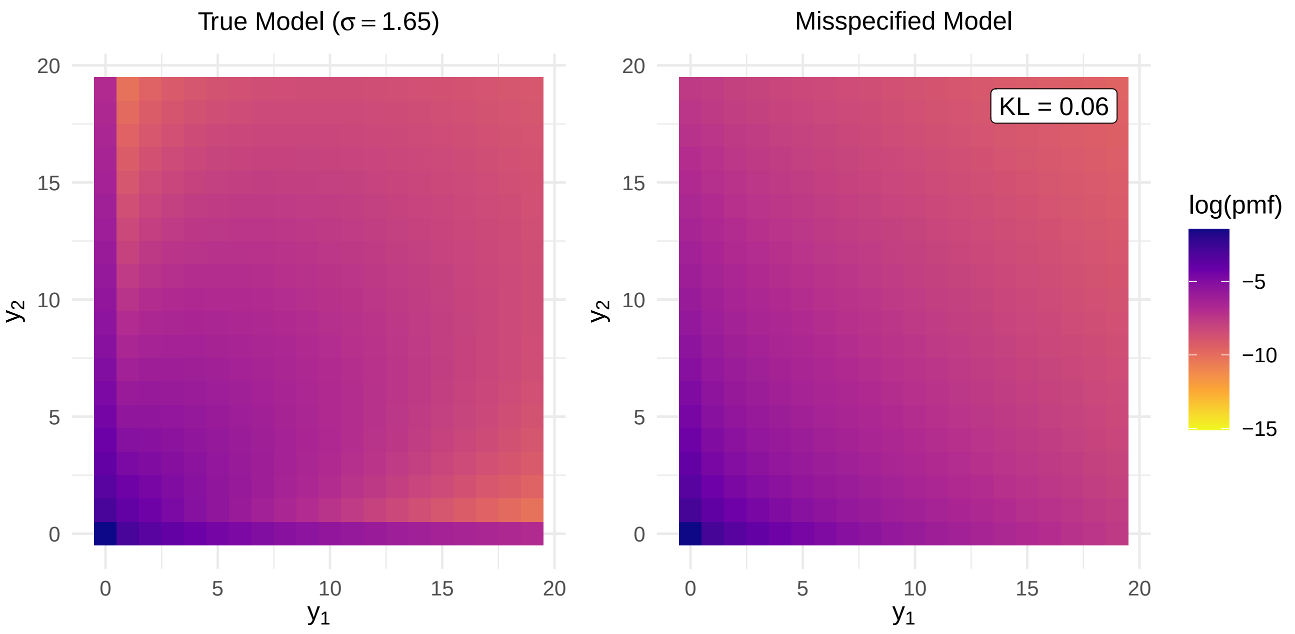

FIGURE S6 Comparison of $\log(f(y_1, y_2\ ;\ \boldsymbol{\theta}))$ and $\log(f^*(y_1, y_2\ ; \boldsymbol{\theta}^*))$ for $\boldsymbol{\theta}$ and $\boldsymbol{\theta}^*$ from Table S7. Plotting domain of y_1, y_2 has been truncated for visual clarity. Note that in this high over-dispersion case, the non-zero-inflated model is better able to capture the shape of the true distribution.

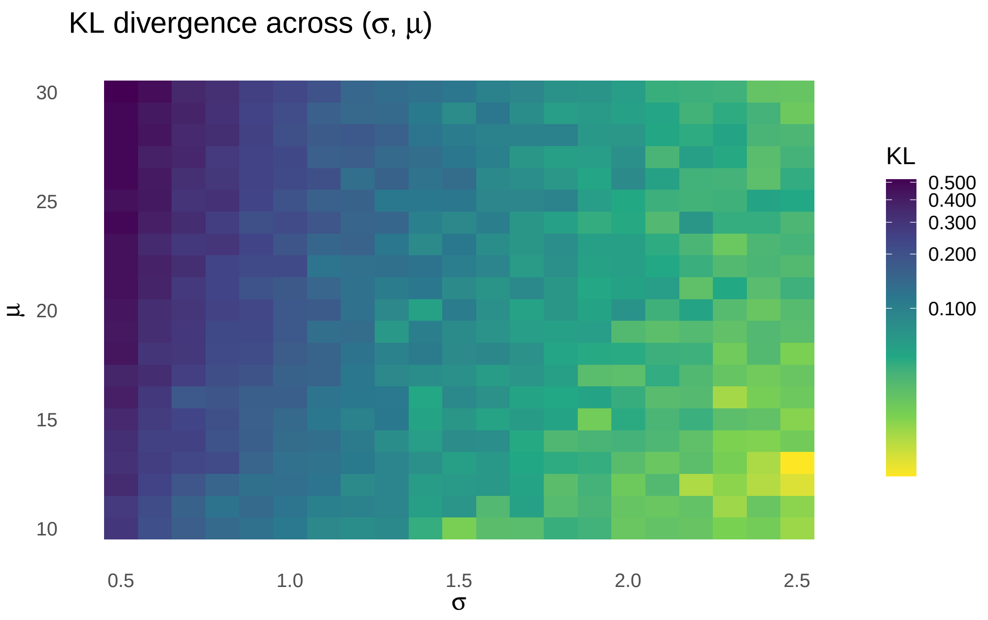

FIGURE S7 KL divergence between scCOSMiX model and the non-zero-inflated version from Equation 1 in Section 2.2 of the main manuscript, computed across a grid of (σ, μ) values. For each (σ, μ) pair on the grid, we set the truth to $\boldsymbol{\theta} = (\mu, \mu, \sigma, \sigma, 0.5, 0.3, 0.3)$ and we compute the KL divergence. This plot demonstrates that the trend observed in Figures S5 and S6, of larger σ corresponding to smaller KL divergence, extends across the range of μ and σ.

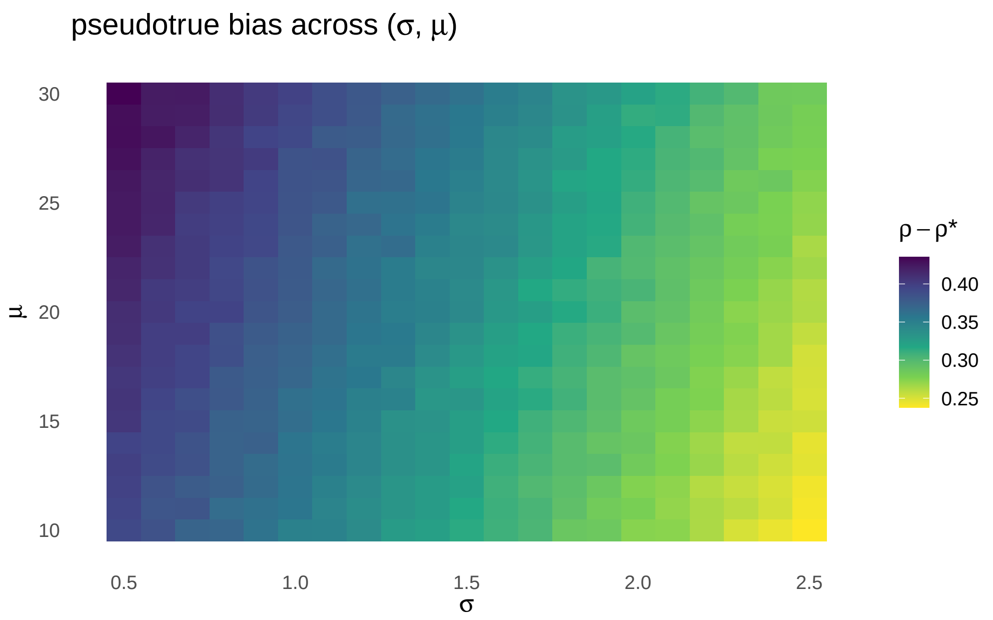

FIGURE S8 Bias of the pseudotrue parameter ρ^* across a grid of (σ, μ) values. The ρ^* corresponding to each (σ, μ) is obtained during the KL divergence computations from Figure S7– where the truth was set to $\boldsymbol{\theta} = (\mu, \mu, \sigma, \sigma, 0.5, 0.3, 0.3)$. We observe a similar trend as was found in Tables S6 and S7: that larger σ corresponds closer proximity of ρ^* to true ρ. Meaning for true distributions with more over-dispersion, the non-zero-inflated model does not attenuate the dependence parameter as drastically as it does for distributions with less over-dispersion.

List of Tables

S1 Plate scenario: Estimation performance for scCOSMiX, evaluated on datasets simulated using parameter values specified in Equation (6) in the main manuscript based on the plate-based dataset GSE108989. Number of patients is set to $m \in \{5, 10, 15\}$, and for each value of m, $B = 500$ datasets are simulated, and estimates and confidence intervals are obtained for each parameter. 39

S2 Plate scenario: Robustness performance for scCOSMiX, evaluated on datasets simulated with parameter values specified in Equation (6) in the main manuscript based on the plate-based dataset GSE108989, with $p_1 = p_2 = 0$. Number of patients is set to $m \in \{5, 10, 15\}$, and for each value of m, $B = 500$ datasets are simulated, and estimates and confidence intervals are obtained for each non-zero-inflation parameter. 40

S3 Droplet scenario: Robustness performance for scCOSMiX, evaluated on datasets simulated with parameter values specified in Equation (5) in the main manuscript based on the droplet-based dataset GSE266919, with $p_2 = 0$. Number of patients is set to $m \in \{5, 10, 15\}$, and for each value of m, $B = 500$ datasets are simulated, and estimates and confidence intervals are obtained for each non-p_2 parameter. 41

S4 Plate scenario: Robustness performance for scCOSMiX, evaluated on datasets simulated with parameter values specified in Equation (6) in the main manuscript based on the plate-based dataset GSE108989, with $p_2 = 0$. Number of patients is set to $m \in \{5, 10, 15\}$, and for each value of m, $B = 500$ datasets are simulated, and estimates and confidence intervals are obtained for each non-p_2 parameter. 42

S5 Plate scenario: Top table of co-expression differences between CD8 and CD4 cells from plate-based CRC dataset GSE108989. 43

S6 True $\boldsymbol{\theta}$ and corresponding pseudotrue $\boldsymbol{\theta}^*$ for low over-dispersion case. The shrunken ρ^* is typical when the true model has low over-dispersion. KL divergence for this case is 0.16. 44

S7 True $\boldsymbol{\theta}$ and corresponding pseudotrue $\boldsymbol{\theta}^*$ for high over-dispersion case. Note that the ρ^* is not shrunken as drastically as in the previous case. KL divergence for this case is = 0.06. 45

TABLE S1 Plate scenario: Estimation performance for scCOSMiX, evaluated on datasets simulated using parameter values specified in Equation (6) in the main manuscript based on the plate-based dataset GSE108989. Number of patients is set to $m \in \{5, 10, 15\}$, and for each value of m, $B = 500$ datasets are simulated, and estimates and confidence intervals are obtained for each parameter.

	**5 Patients**				**10 Patients**				**15 Patients**			
**Parameter**	Coverage	MSE	MBE	CI Width	Coverage	MSE	MBE	CI Width	Coverage	MSE	MBE	CI Width
β_{01}	0.902	0.013	0.090	0.446	0.908	0.007	0.068	0.321	0.950	0.004	0.052	0.266
β_{11}	0.930	0.032	0.142	0.721	0.940	0.016	0.101	0.514	0.954	0.011	0.084	0.422
β_{02}	0.898	0.015	0.099	0.472	0.942	0.008	0.071	0.348	0.942	0.005	0.056	0.281
β_{12}	0.942	0.035	0.146	0.751	0.942	0.017	0.103	0.533	0.944	0.011	0.083	0.431
α_{01}	0.954	0.005	0.056	0.278	0.954	0.003	0.040	0.196	0.944	0.002	0.033	0.160
α_{11}	0.944	0.024	0.121	0.593	0.938	0.012	0.089	0.424	0.960	0.007	0.067	0.347
α_{02}	0.954	0.007	0.069	0.347	0.922	0.005	0.055	0.245	0.954	0.003	0.042	0.200
α_{12}	0.932	0.030	0.135	0.655	0.956	0.014	0.093	0.464	0.954	0.010	0.080	0.379
τ_0	0.964	0.003	0.046	0.263	0.976	0.002	0.033	0.181	0.954	0.001	0.028	0.147
τ_1	0.964	0.009	0.077	0.443	0.944	0.005	0.057	0.332	0.962	0.003	0.045	0.246
p_{01}	0.974	0.000	0.011	0.065	0.966	0.000	0.008	0.044	0.964	0.000	0.007	0.035
p_{11}	0.938	0.002	0.036	0.206	0.930	0.001	0.027	0.131	0.942	0.001	0.019	0.104
p_{02}	0.946	0.001	0.019	0.099	0.946	0.000	0.014	0.068	0.960	0.000	0.011	0.056
p_{12}	0.930	0.002	0.038	0.189	0.940	0.001	0.025	0.127	0.948	0.001	0.021	0.103

TABLE S2 Plate scenario: Robustness performance for scCOSMiX, evaluated on datasets simulated with parameter values specified in Equation (6) in the main manuscript based on the plate-based dataset GSE108989, with $p_1 = p_2 = 0$. Number of patients is set to $m \in \{5, 10, 15\}$, and for each value of m, $B = 500$ datasets are simulated, and estimates and confidence intervals are obtained for each non-zero-inflation parameter.

	**5 Patients**				**10 Patients**				**15 Patients**			
**Parameter**	Coverage	MSE	MBE	CI Width	Coverage	MSE	MBE	CI Width	Coverage	MSE	MBE	CI Width
β_{01}	0.890	0.013	0.093	0.436	0.920	0.007	0.066	0.317	0.936	0.004	0.051	0.266
β_{11}	0.944	0.031	0.140	0.694	0.938	0.016	0.100	0.492	0.942	0.009	0.073	0.403
β_{02}	0.912	0.014	0.097	0.463	0.912	0.008	0.069	0.332	0.942	0.005	0.056	0.280
β_{12}	0.920	0.031	0.143	0.692	0.940	0.017	0.104	0.495	0.954	0.011	0.083	0.414
α_{01}	0.938	0.004	0.050	0.240	0.954	0.002	0.034	0.169	0.948	0.001	0.029	0.139
α_{11}	0.926	0.015	0.095	0.437	0.926	0.008	0.067	0.313	0.944	0.005	0.054	0.258
α_{02}	0.940	0.005	0.055	0.263	0.920	0.003	0.044	0.190	0.954	0.002	0.032	0.156
α_{12}	0.922	0.014	0.094	0.449	0.928	0.008	0.069	0.327	0.924	0.006	0.057	0.273
τ_0	0.956	0.003	0.042	0.228	0.940	0.002	0.031	0.155	0.962	0.001	0.022	0.200
τ_1	0.952	0.006	0.062	0.406	0.958	0.003	0.043	0.224	0.961	0.002	0.032	0.256

TABLE S3 Droplet scenario: Robustness performance for scCOSMiX, evaluated on datasets simulated with parameter values specified in Equation (5) in the main manuscript based on the droplet-based dataset GSE266919, with $p_2 = 0$. Number of patients is set to $m \in \{5, 10, 15\}$, and for each value of m, $B = 500$ datasets are simulated, and estimates and confidence intervals are obtained for each non-p_2 parameter.

	**5 Patients**				**10 Patients**				**15 Patients**			
**Parameter**	Coverage	MSE	MBE	CI Width	Coverage	MSE	MBE	CI Width	Coverage	MSE	MBE	CI Width
β_{01}	0.910	0.011	0.083	0.393	0.912	0.006	0.063	0.291	0.929	0.004	0.049	0.240
β_{11}	0.920	0.021	0.115	0.585	0.926	0.012	0.087	0.420	0.945	0.008	0.072	0.344
β_{02}	0.884	0.011	0.085	0.402	0.920	0.006	0.061	0.296	0.927	0.004	0.050	0.238
β_{12}	0.916	0.023	0.125	0.577	0.916	0.013	0.089	0.417	0.943	0.008	0.070	0.339
α_{01}	0.930	0.007	0.068	0.322	0.920	0.004	0.048	0.228	0.952	0.002	0.038	0.186
α_{11}	0.944	0.016	0.099	0.480	0.948	0.007	0.068	0.344	0.970	0.005	0.055	0.283
α_{02}	0.956	0.006	0.060	0.301	0.952	0.003	0.043	0.215	0.935	0.002	0.039	0.177
α_{12}	0.970	0.010	0.082	0.438	0.964	0.006	0.062	0.311	0.952	0.004	0.051	0.256
τ_0	0.960	0.003	0.043	0.326	0.954	0.001	0.031	0.157	0.970	0.001	0.024	0.127
τ_1	0.970	0.005	0.059	0.421	0.970	0.003	0.043	0.221	0.972	0.002	0.033	0.178
p_{01}	0.970	0.000	0.010	0.053	0.956	0.000	0.007	0.037	0.941	0.000	0.006	0.029
p_{11}	0.888	0.000	0.009	0.356	0.902	0.000	0.007	0.209	0.909	0.000	0.006	0.170

TABLE S4 Plate scenario: Robustness performance for scCOSMiX, evaluated on datasets simulated with parameter values specified in Equation (6) in the main manuscript based on the plate-based dataset GSE108989, with $p_2 = 0$. Number of patients is set to $m \in \{5, 10, 15\}$, and for each value of m, $B = 500$ datasets are simulated, and estimates and confidence intervals are obtained for each non-p_2 parameter.

	**5 Patients**				**10 Patients**				**15 Patients**			
**Parameter**	Coverage	MSE	MBE	CI Width	Coverage	MSE	MBE	CI Width	Coverage	MSE	MBE	CI Width
β_{01}	0.898	0.014	0.094	0.638	0.918	0.007	0.065	0.323	0.936	0.004	0.054	0.266
β_{11}	0.946	0.031	0.142	0.925	0.942	0.016	0.101	0.510	0.952	0.011	0.083	0.422
β_{02}	0.884	0.017	0.106	1.041	0.926	0.007	0.068	0.337	0.926	0.005	0.058	0.274
β_{12}	0.916	0.034	0.149	1.633	0.944	0.015	0.098	0.502	0.926	0.011	0.082	0.410
α_{01}	0.952	0.005	0.056	0.273	0.930	0.003	0.041	0.194	0.944	0.002	0.033	0.158
α_{11}	0.952	0.020	0.115	0.591	0.942	0.013	0.091	0.423	0.936	0.009	0.075	0.346
α_{02}	0.938	0.005	0.057	0.263	0.916	0.003	0.043	0.187	0.932	0.002	0.034	0.154
α_{12}	0.918	0.016	0.101	0.450	0.922	0.008	0.067	0.322	0.934	0.005	0.056	0.270
τ_0	0.964	0.003	0.044	0.687	0.950	0.002	0.032	0.162	0.958	0.001	0.026	0.130
τ_1	0.964	0.007	0.066	1.014	0.934	0.004	0.052	0.247	0.964	0.002	0.039	0.200
p_{01}	0.954	0.000	0.012	0.065	0.958	0.000	0.009	0.044	0.952	0.000	0.007	0.035
p_{11}	0.934	0.002	0.036	0.213	0.922	0.001	0.027	0.134	0.924	0.001	0.023	0.106

TABLE S5 Plate scenario: Top table of co-expression differences between CD8 and CD4 cells from plate-based CRC dataset GSE108989.

#	Gene 1	Gene 2	ρ (CD4)	ρ (CD8)	$\lvert\Delta\rho\rvert$
1	BATF	THEMIS	-0.361	0.103	0.464
2	ABLIM1	IL21R	-0.306	0.076	0.383
3	BATF	CLEC2B	-0.361	0.016	0.377
4	ABLIM1	LGALS1	-0.350	0.026	0.376
5	CLEC2B	THEMIS	0.433	0.059	0.374
6	BATF	PDE4DIP	-0.237	0.119	0.357
7	BATF	INPP4B	-0.208	0.146	0.354
8	BATF	IL21R	0.416	0.062	0.354
9	CLEC2B	GLRX	-0.259	0.092	0.351
10	EDARADD	LGALS1	0.238	-0.105	0.342
11	CARD16	THEMIS	-0.266	0.066	0.332
12	DDHD1	CD6	-0.270	0.059	0.330
13	CD28	BATF	0.232	-0.096	0.329
14	ITGB7	BATF	-0.278	0.050	0.327
15	BATF	GLRX	0.341	0.014	0.327
16	CARD16	CD28	0.209	-0.117	0.326
17	GLRX	APLP2	0.278	-0.047	0.325
18	ABLIM1	BATF	-0.385	-0.060	0.325
19	C16orf54	CD6	0.376	0.053	0.323
20	BATF	NCOA3	0.268	-0.054	0.322
21	BATF	PBXIP1	0.197	-0.124	0.321
22	BATF	C16orf54	-0.335	-0.015	0.320
23	STK17A	TYMP	-0.101	0.217	0.319
24	C16orf54	CARD16	-0.279	0.036	0.315
25	APLP2	CARD16	0.327	0.013	0.313

TABLE S6 True $\boldsymbol{\theta}$ and corresponding pseudotrue $\boldsymbol{\theta}^*$ for low over-dispersion case. The shrunken ρ^* is typical when the true model has low over-dispersion. KL divergence for this case is 0.16.

	$\boldsymbol{\theta}$	$\boldsymbol{\theta}^*$
μ_1	5.00	3.98
μ_2	5.00	3.98
σ_1	0.60	1.25
σ_2	0.60	1.25
ρ	0.75	0.36
p_1	0.20	–
p_2	0.20	–

TABLE S7 True $\boldsymbol{\theta}$ and corresponding pseudotrue $\boldsymbol{\theta}^*$ for high over-dispersion case. Note that the ρ^* is not shrunken as drastically as in the previous case. KL divergence for this case is = 0.06.

	$\boldsymbol{\theta}$	$\boldsymbol{\theta}^*$
μ_1	5.00	3.96
μ_2	5.00	3.96
σ_1	1.65	2.58
σ_2	1.65	2.58
ρ	0.75	0.47
p_1	0.20	–
p_2	0.20	–
