# Supplementary figures and images for: scCOSMIX: A Mixed‐Effects Framework for Differential Coexpression and Transcriptional Interactions Modeling in Single‐Cell RNA‐Seq
Source: Stat Med. 2025 Aug 7;44(18-19):e70213. doi: 10.1002/sim.70213 (PMC12330344; doi:10.1002/sim.70213)

Power by number of patients

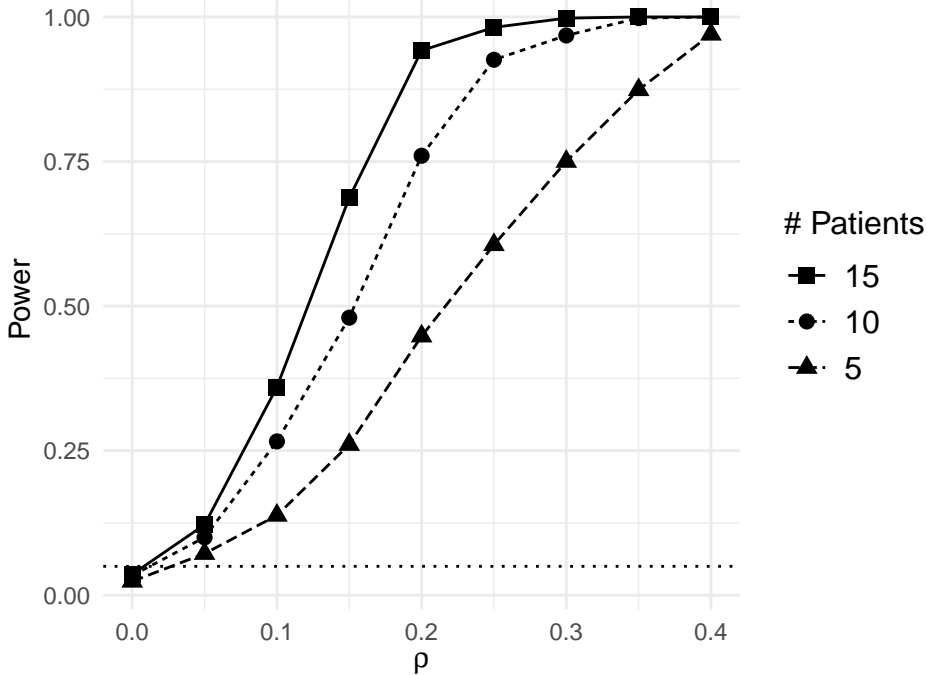

Supplement: Supplementary file 1 — Data S1. Additional supporting information, including additional plots and tables referenced in the text, a Kullback‐Leibler divergence study, and derivations of the gradient and hessian may be found in the online version of the article at the publisher's website. [file SIM-44-0-s001.zip › Bussing_FigS1.pdf]

## Average precision–recall

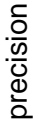

Supplement: Supplementary file 1 — Data S1. Additional supporting information, including additional plots and tables referenced in the text, a Kullback‐Leibler divergence study, and derivations of the gradient and hessian may be found in the online version of the article at the publisher's website. [file SIM-44-0-s001.zip › Bussing_FigS3.pdf]

$\rho_{CD4}$ 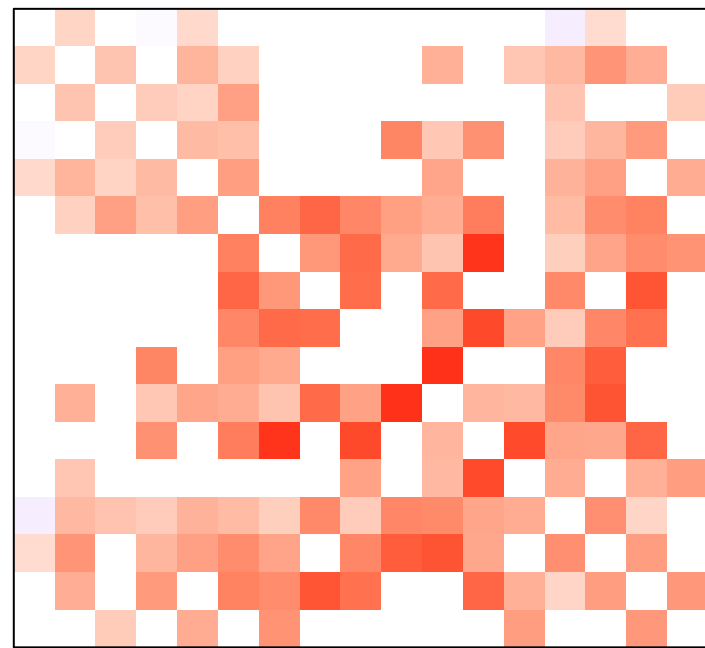 $\rho_{CD8}$ 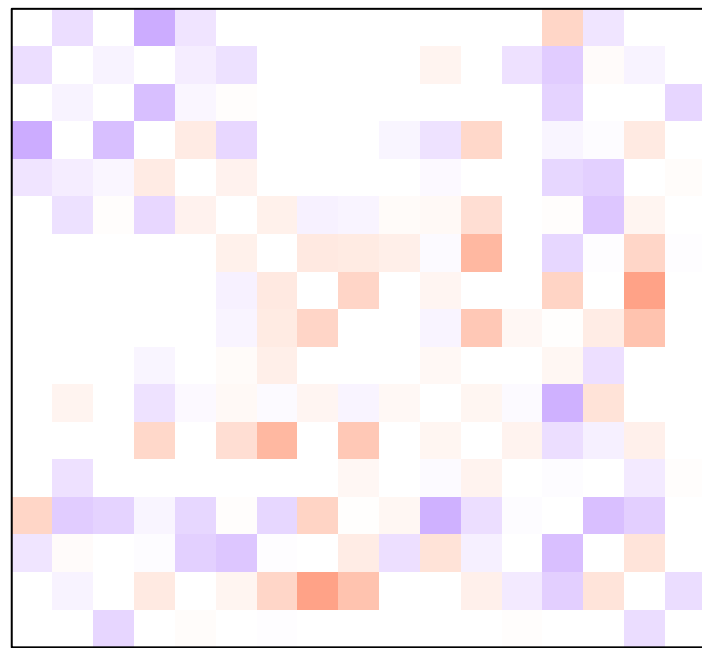 $\rho_{CD8} - \rho_{CD4}$ 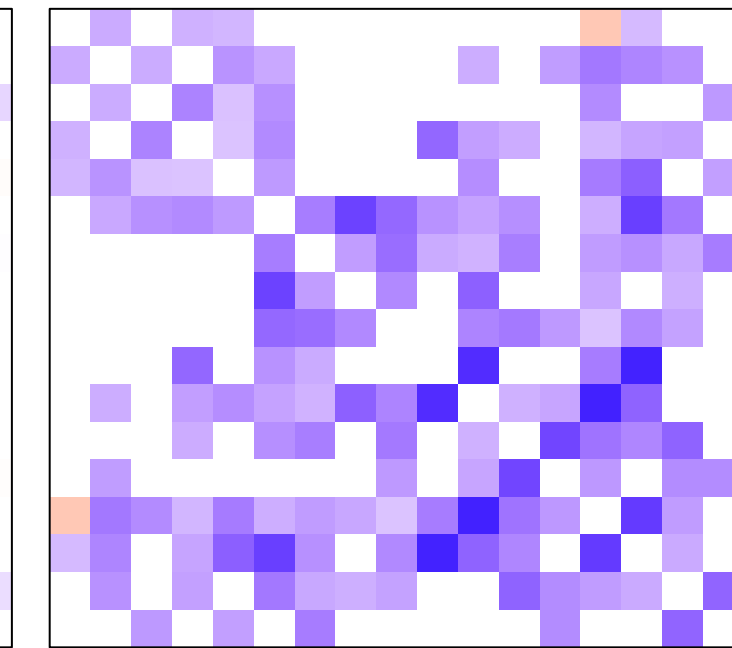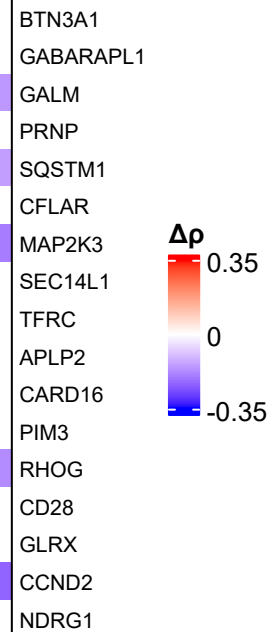

Supplement: Supplementary file 1 — Data S1. Additional supporting information, including additional plots and tables referenced in the text, a Kullback‐Leibler divergence study, and derivations of the gradient and hessian may be found in the online version of the article at the publisher's website. [file SIM-44-0-s001.zip › Bussing_FigS4.pdf]

True Model ( $\sigma = 0.6$ )

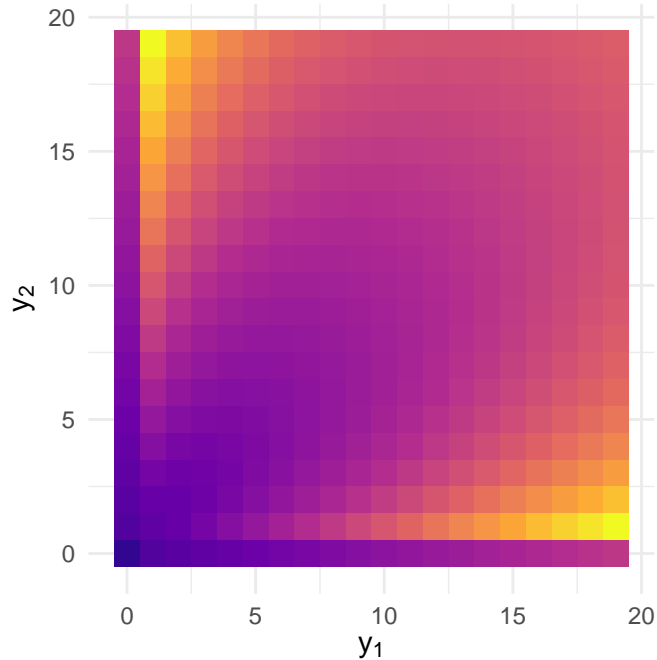

Misspecified Model

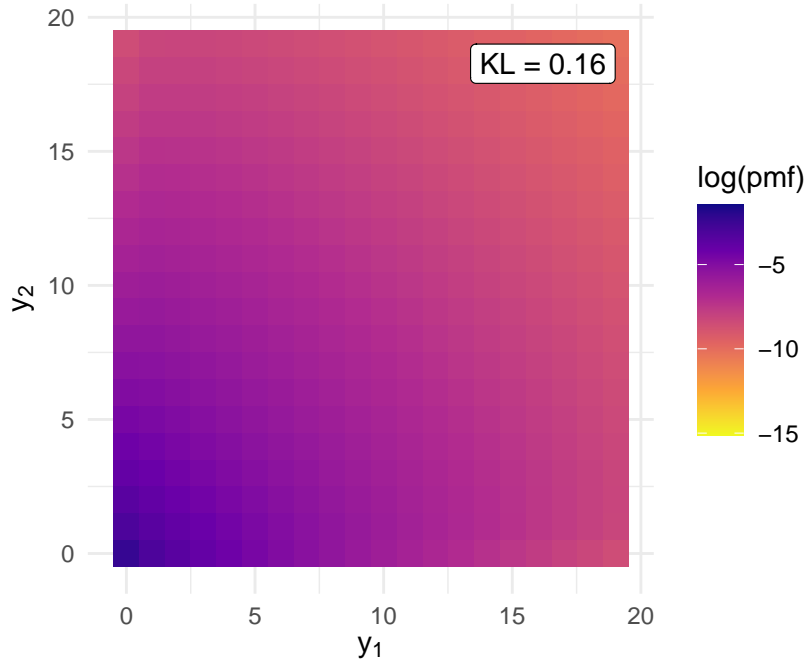

Supplement: Supplementary file 1 — Data S1. Additional supporting information, including additional plots and tables referenced in the text, a Kullback‐Leibler divergence study, and derivations of the gradient and hessian may be found in the online version of the article at the publisher's website. [file SIM-44-0-s001.zip › Bussing_FigS5.pdf]

True Model ( $\sigma = 1.65$ )

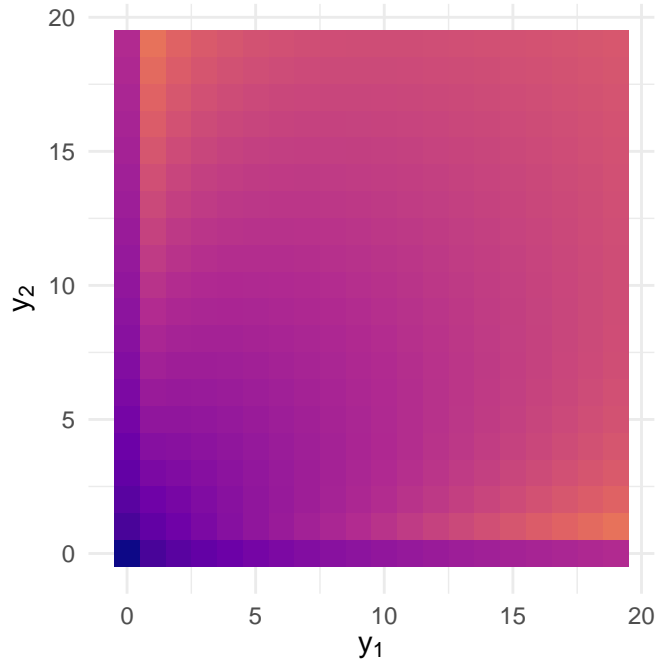

Misspecified Model

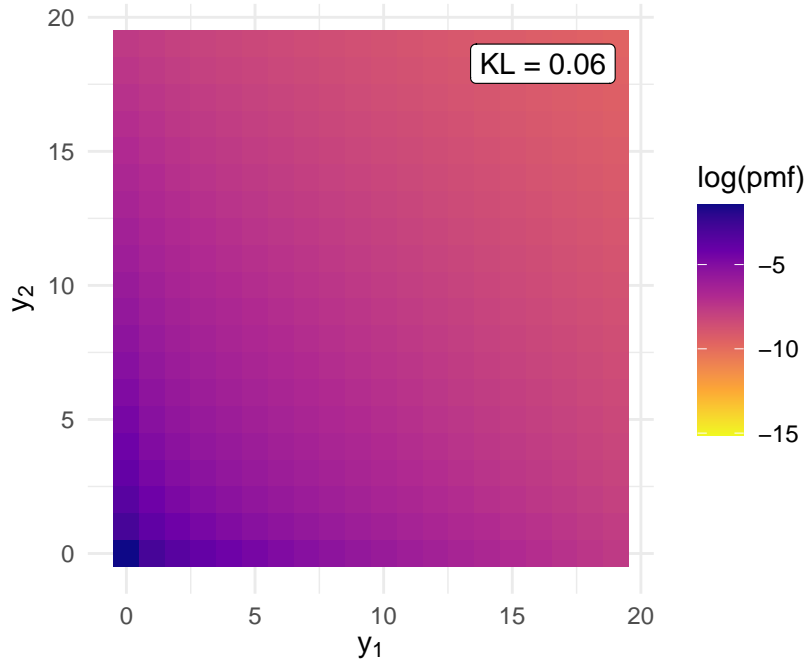

Supplement: Supplementary file 1 — Data S1. Additional supporting information, including additional plots and tables referenced in the text, a Kullback‐Leibler divergence study, and derivations of the gradient and hessian may be found in the online version of the article at the publisher's website. [file SIM-44-0-s001.zip › Bussing_FigS6.pdf]

KL divergence across  $(\sigma, \mu)$

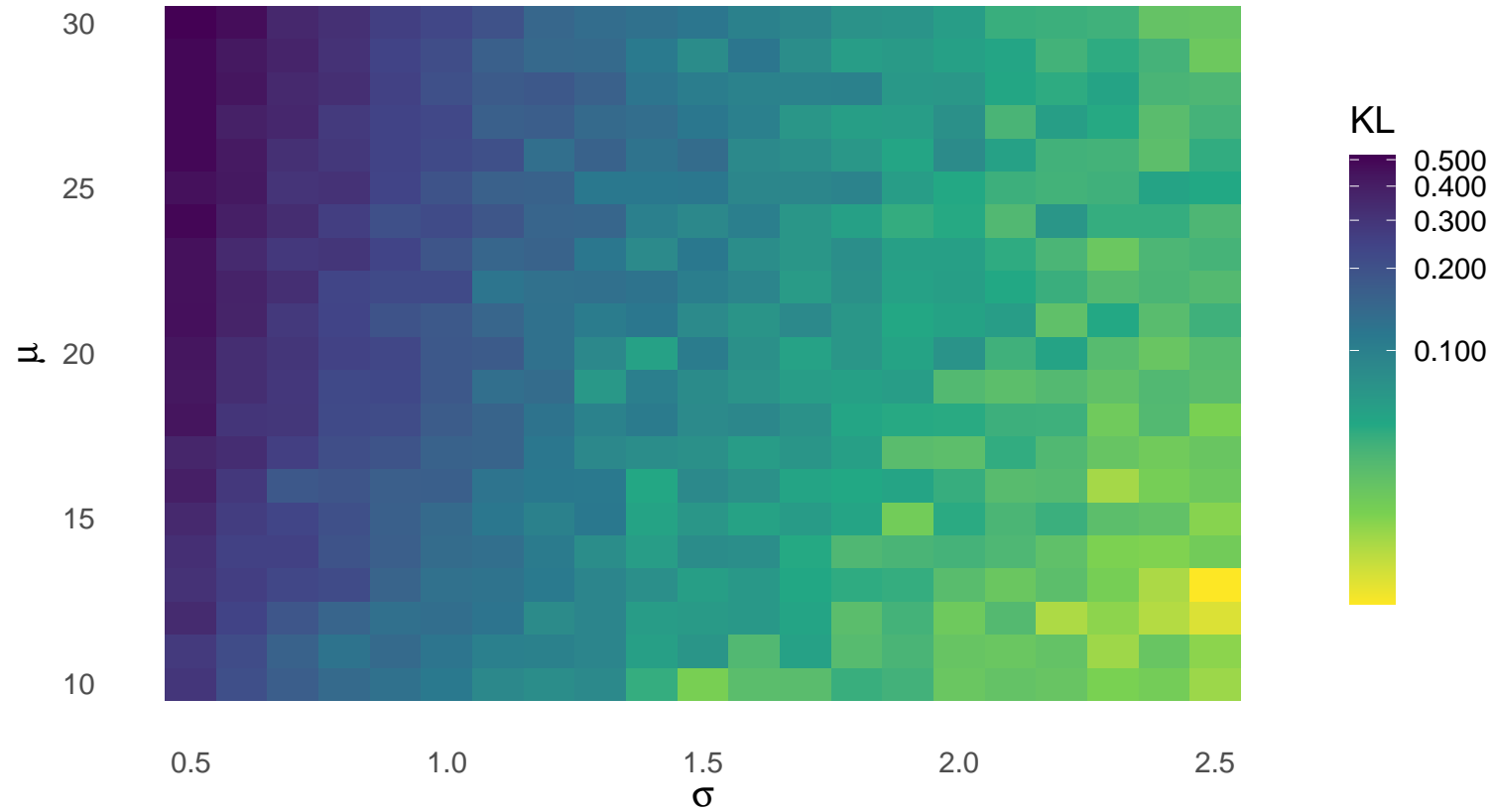

Supplement: Supplementary file 1 — Data S1. Additional supporting information, including additional plots and tables referenced in the text, a Kullback‐Leibler divergence study, and derivations of the gradient and hessian may be found in the online version of the article at the publisher's website. [file SIM-44-0-s001.zip › Bussing_FigS7.pdf]

pseudotrue bias across  $(\sigma, \mu)$

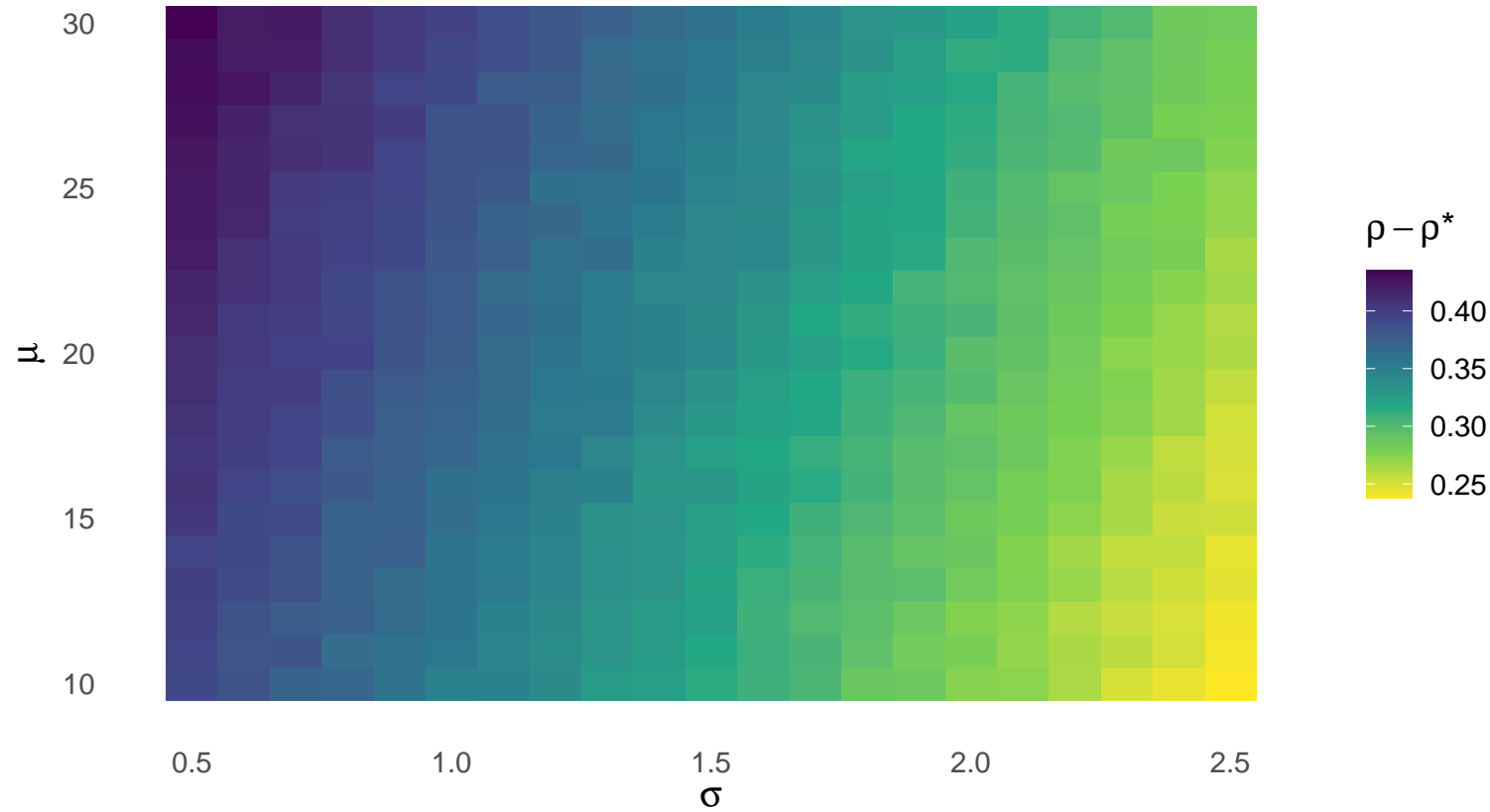

Supplement: Supplementary file 1 — Data S1. Additional supporting information, including additional plots and tables referenced in the text, a Kullback‐Leibler divergence study, and derivations of the gradient and hessian may be found in the online version of the article at the publisher's website. [file SIM-44-0-s001.zip › Bussing_FigS8.pdf]
